# The Effects of Heparin Binding and Arg596 Mutations on the Conformation of Thrombin–Antithrombin Michaelis Complex, Revealed by Enhanced Sampling Molecular Dynamics Simulations

**DOI:** 10.3390/ijms26209901

**Published:** 2025-10-11

**Authors:** Gábor Balogh, Zsuzsanna Bereczky

**Affiliations:** Division of Clinical Laboratory Science, Department of Laboratory Medicine, Faculty of Medicine, University of Debrecen, H-4032 Debrecen, Hungary

**Keywords:** antithrombin, thrombin, mutation, molecular dynamics, Michaelis complex, enhanced sampling molecular dynamics simulation

## Abstract

The inactivation of thrombin by antithrombin is highly enhanced by the presence of heparin chains forming “bridges” between the two proteins. X-ray structures for such ternary complexes have been published, but the molecular background of the lower efficiency of smaller heparinoids on thrombin inhibition remains poorly understood. Antithrombin-resistant prothrombin mutants (mutations affecting Arg596 in prothrombin) have been reported that cause severe thrombophilia. Our aim was to study the interactions in the antithrombin–thrombin Michaelis complex both in the presence and the absence of a heparinoid chain and in the presence of pentasaccharide by using molecular dynamics. We also intended to study the complexes of thrombin mutants as well as a known alternative antithrombin conformation at the “hinge” region built using docking. The binding between the proteins was investigated by Gaussian Accelerated Molecular Dynamics (GaMD). We compared the contribution of several amino acids at the binding “exosites” between AT and the wild type and mutant thrombins and between systems containing or not containing a heparinoid. In the docking-based simulations, several of the analyzed amino acid pairs no longer contributed to the interaction, suggesting that the open “hinge” conformation has limited biological relevance. We could identify multiple conformational types using clustering, revealing high flexibility in mutants and systems without heparinoid, probably indicating lower stability. We were also able to detect the allosteric effects of the ligands on the bound thrombin. In summary, we were able to obtain conformations using GaMD that can explain the better protein–protein interactions in the ternary complexes and the impaired AT binding of the thrombin Arg596 mutants at an atomic level.

## 1. Introduction

Thrombin (FIIa) is the central enzyme of the coagulation cascade [1,2]. Its inactive precursor form prothrombin (factor II) is proteolytically activated by active factor Xa, in the presence of factor Va, phospholipids and calcium ions on the surface of activated platelets [2]. Thrombin is the enzyme responsible for converting fibrinogen to fibrin, as well as the activation of XIa, Va, VIIIa and XIIIa factors [3].

Thrombin also affects the cellular component of coagulation, mainly by activating thrombocytes by cleaving their PAR-1 receptor [2]. In contrast, FIIa also exhibits anti-coagulant functions: in the presence of thrombomodulin, it activates protein C, which in turn converts factors Va and VIIIa into less active or inactive forms by proteolytic cleavage [3].

The most important physiological inhibitor is antithrombin (AT), a member of the serin protease inhibitor (serpin) superfamily [4]. This plasma glycoprotein inactivates thrombin and other coagulation factors (especially active factor X and IX) by an irreversible mechanism in which the enzyme remains covalently bound to the serpin after significant conformational changes in both proteins [5]. For the inhibition of thrombin by AT, the factor must first non-covalently bind to the reactive center loop (RCL) of the serpin for the formation of a Michaelis complex where the Arg393 of the RCL plays a critical role [6,7,8]. (For residues in antithrombin, we apply a numbering that corresponds to a mature, secreted protein and does not include a 32-amino-acid-long propeptide. Multiple X-ray diffraction structures of AT including 1TB6 [6] and 1E03 [9] use this numbering.) However, for efficient interaction, the involvement of several loops of thrombin is required with sites in AT outside the RCL. One of the critical exosite residues is Arg596 (Arg221a in the chymotrypsin numbering of the catalytic domain which is bound to a site in AT near amino acids Glu232 and Asn233). The γ-loop of thrombin (147–149 in chymotrypsin numbering) contains further amino acids involved in the binding [1,6,10]. (In the analysis of our simulation results, we apply the chymotrypsin numbering for thrombin, which is used for multiple X-ray diffraction structures as well, including chain H in the structure 1TB6 [6]).

In the presence of a heparinoid molecule that is long enough to form a bridge between thrombin and AT, the reaction rate of inhibition increases by multiple order of magnitudes [6,11]. Such molecules have an interaction site on both the catalytic domain of AT and on thrombin; the interaction site in the latter is referred to as exosite II [1,6]. Importantly, high-affinity pentasaccharides such as fondaparinux exist that can bind to the interaction site of AT, but such molecules are too small to interact with the heparin binding site of thrombin at the same time. Such molecules can drastically enhance the FXa and FIXa inhibition by AT, but have much smaller effects on AT–thrombin interaction [11,12]. Such ligands trigger a series of conformational changes in AT, but the relevance of this mechanism is limited in the case of thrombin [13].

**Figure 1:**
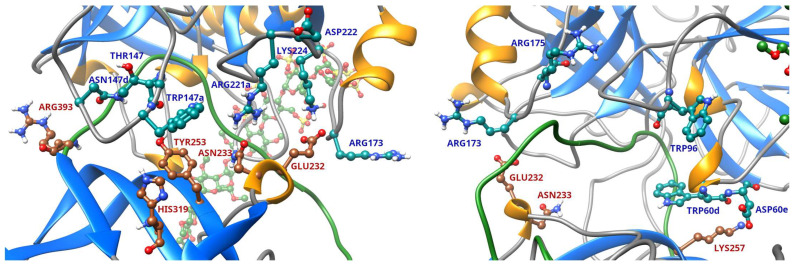
The most important amino acids involved in “exosite” interactions between thrombin and AT in the Michaelis complex in the 1TB6 X-ray structure. The interaction sites in this structure are shown from two different angles to make all important residues visible. The most important amino acids involved in the interaction are labeled (red in AT, blue in thrombin).

The details of AT–thrombin interaction in the Michaelis complex was investigated at an atomic level in X-ray diffraction studies [6,10]. The system investigated by Li et al. was a ternary complex in which a sulfated heparin-like molecule interacted with both proteins that stabilized the complex significantly (PDB: 1TB6) [6]. This ternary complex structure revealed the important details of AT–thrombin interaction in the Michaelis complex. The most important sites of interaction in this structure are depicted in Figure 1. However, no experimental structure is available for the Michaelis complex between thrombin and AT without a heparin chain facilitating the binding. Furthermore, it is also known that a specific pentasaccharide sequence—too small for forming a ternary complex—can also interact with AT with a very high affinity. The binding of such a molecule can enhance the inactivation rates for factors Xa and IXa through an allosteric mechanism by triggering a complex set of conformational changes in the serpin [11]. One of the most important steps of this process involves the “hinge” region, the N-terminal part of the RCL. In the X-ray diffraction structures of non-activated AT, the hinge region is found in closed position in the beta sheet “A”, but it can transit into an “open” conformation outside the sheet. However, these changes have only a modest effect on thrombin inhibition. This observation cannot be fully explained by the available structure. Furthermore, according to X-ray diffraction studies, binding of heparinoids results in a conformational change in the N-terminal end of the reactive center loop called the hinge region, resulting in its expulsion from its locked position of the beta sheet A [9,14]. Such a conformation is also observed in the structures of AT complexed with FXa and FIXa [15,16]. However, in the AT–thrombin–heparinoid complexes, this region has the “closed” conformation, as evidenced by the X-ray structures [6,10]. However, it is not known whether AT with “open” hinge region can interact with thrombin.

In the literature, several thrombin mutants have been described that impair the inactivation of thrombin by AT, including Prothrombin Yukuhashi (Arg221aLeu, p.Arg596Leu) [17], Belgrade (Arg221aGln, p.Arg596Gln) [18], Padua 2 (Arg221aTrp, p.Arg596Trp) [19]. For each mutant, the first number corresponds to the chymotrypsin numbering, while the second is the location of the amino acid in the sequence of human prothrombin in UniProt (accession number P00734). These mutations cause a severe form of thrombophilia in the affected patients. The mutations affect one amino acid located in the Na^+^ binding loop, which is part of an interaction exosite. Despite the fact that these mutant thrombins have lower than normal activity in vitro, the consequence is a predisposition for thrombosis due to their defective inhibition [20]. Tamura et al. have published detailed in vitro data on how several mutations in the same region of the protein affect the activity of the mutants and their inactivation by AT [20]. These observations further support the high importance of the exosites in the interactions.

Using protein docking and molecular dynamics (MD), it is possible to study proteins and protein complexes at an atomic level based on the existing experimental data, even when no X-ray structure of some potentially important conformations is available. Another advantage of MD-based techniques is that they allow sampling of multiple conformations of complexes in contrast to X-ray diffraction data which are typically limited to one or very few possible states of the system. Both thrombin and AT were subjects of several molecular-dynamics-based studies. For AT, the consequences of pentasaccharide binding [21,22], the effects of Asn135 glycosylation [23] and the conformation of the Arg393 residue in solution were investigated [8]. Our group has published multiple MD-based studies about the consequences of AT heparin binding site mutants [24,25].

Using molecular dynamics simulations, Xiao et al. investigated the allosteric behavior and Na^+^ ion binding of thrombin [26]. In an important study, Pol-Fachin et al. have studied the binding of heparin-like molecules to the three most important target factors, thrombin, factor Xa and factor IXa, using molecular dynamics simulations [27]. However, neither AT-factor Michaelis complexes, nor ternary complexes containing a heparinoid molecule forming a bridge between the two proteins, were modeled in this work.

In one of our previous papers, we have investigated AT–factor Xa and AT–factor IXa Michaelis complexes using Gaussian Accelerated Molecular Dynamics (GaMD) [28] simulations, an enhanced sampling simulation technique. We built models for both complexes that contained an AT conformation not activated by heparinoid ligands and with closed “hinge” conformations using protein docking [29]. In addition, we compared these results to simulations based on the available X-ray diffraction structures in which the hinge was in a fully open state [29]. Our simulations provided insights into the possible conformational types in AT–FXa and AT–FIXa complexes and the allosteric effects of heparin pentasaccharide binding on the interactions [29].

In the present paper, we performed molecular dynamics simulations on multiple AT–thrombin Michaelis complex models for better understanding the molecular basis of interaction between the two proteins. One of the main aims of our study was to build a model for the AT–thrombin Michaelis complex in the absence of heparinoids. The available X-ray diffraction structures contain a heparin-like molecule and therefore cannot provide a complete understanding of the interaction of the two proteins in the Michaelis complex when such a molecule is not present. In addition, we simulated a complex in which AT was complexed with the high-affinity pentasaccharide fondaparinux [30], which is too small for forming a ternary complex. With such a model, we could elucidate why the binding of such a molecule has insignificant effects on the AT–thrombin interaction, despite its ability to trigger ‘activating’ conformational changes for enhanced FXa and FIXa inactivation. Furthermore, we performed molecular docking to build a structure for an AT–thrombin complex where AT is in a conformation with a closed “hinge” region. Our GaMD molecular dynamics simulations of these structures with or without heparinoid ligands have revealed that several amino acids in thrombin known to be involved in the interactions with the “exosites” AT can no longer significantly contribute to the binding. This provides an explanation why the states with a closed “hinge” are the preferred form of AT in the Michaelis complex. Simulation of three thrombin mutants affecting amino acid 221a (in chymotrypsin numbering) was also an important aim of our study; these simulations provided insight into how these amino acid changes lead to impaired inactivation of thrombin by AT.

To our knowledge, this is the first study in which AT–thrombin complexes were simulated using molecular-dynamics-based techniques and also the first long molecular dynamics simulations involving prothrombin mutants causing AT resistance.

## 2. Results

### 2.1. Structure of the Complex of Thrombin with AT in an Open “Hinge” Region Conformation Obtained by Docking

**Figure 2:**
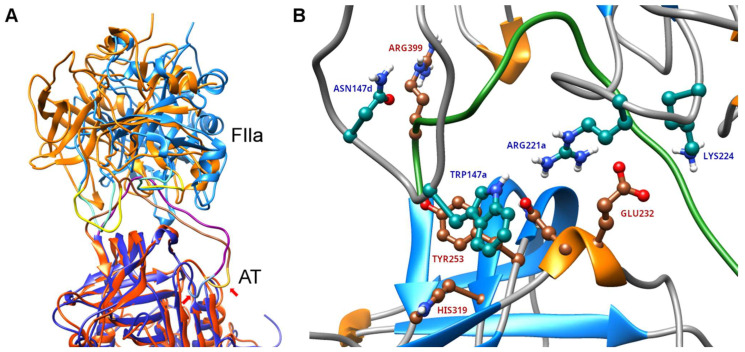
(**A**) The position of thrombin in the structure of the complex obtained by docking, compared to the 1TB6 X-ray structure. To compare the position of thrombin in the two complexes, we performed RMS alignment using the alpha-carbon atoms of AT. The proteins are colored blue in the X-ray structure and orange in the docked structure. The loops involved in “exosite” interactions are shown in light blue and yellow, respectively. The RCL of AT as well as the hinge region is also highlighted, showing the differences between the two conformational types of AT. The hinge region is additionally marked with small arrows. (**B**) The most important residues involved in interaction between the exosites of thrombin and AT in the structure obtained by docking. The RCL of AT is colored green. The most important amino acids involved in the interaction are labeled (red in AT, blue in thrombin).

We have used protein docking to build structures for AT–thrombin Michaelis complexes where AT is in a conformation with an open “hinge” region. To build this complex, we have obtained a suitable initial conformation of the reactive center loop (RCL) using a GaMD simulation of structure 1E03 which already has an open hinge region conformation. A comparison between the position of thrombin in the complex and its conformation in the X-ray structure is shown in Figure 2A. In Figure 2B, the most important amino acids involved in the interaction are shown as well.

We have used HADDOCK 2.4 for docking; the main advantage of this method is that it allows defining amino acids known to be involved in the interaction, making it useful for building complexes where this information is available (the used restraints are discussed in Methods Section 4.3). HADDOCK was able to build a complex structure with an open “hinge” conformation for further simulations. In this structure, the Arg393 residue in AT was positioned correctly compared to the catalytic Ser195 residue of thrombin; additionally, the Asp189 amino acid was located in the binding pocket for the positively charged side chain. The Arg221a amino acid of thrombin was also in its expected position at its binding site at the “exosite” of AT near the Asn233 residue. The important Trp147a amino acid of the γ-loop was located near Tyr253 in AT, at its expected interaction site. However, the loop was shifted significantly compared to its position in the X-ray diffraction structure; it is capable of such an “adaptation” due to its high conformational flexibility. The extent of conformational change required for the binding may suggest that the interactions between AT and the thrombin exosites may be less favorable in the “open hinge” state compared to the binding mode determined by X-ray diffraction.

### 2.2. Exosite Interactions in Protein Complexes Based on the X-Ray Diffraction Structure with a “Closed” Hinge Region in AT

The reactive center loop of AT is not sufficient for effective interaction with thrombin in the Michaelis complex [1,31]. Thrombin contains multiple amino acids in surface loops that form additional contacts with AT, referred to as “exosite” interactions. The most important residues are located in the γ-loop of thrombin (146–149 in chymotrypsin numbering) and a binding site for sodium ions (221–224). Further regions that contribute to the binding include the 60-loop (in chymotrypsin numbering) [6].

**Figure 3:**
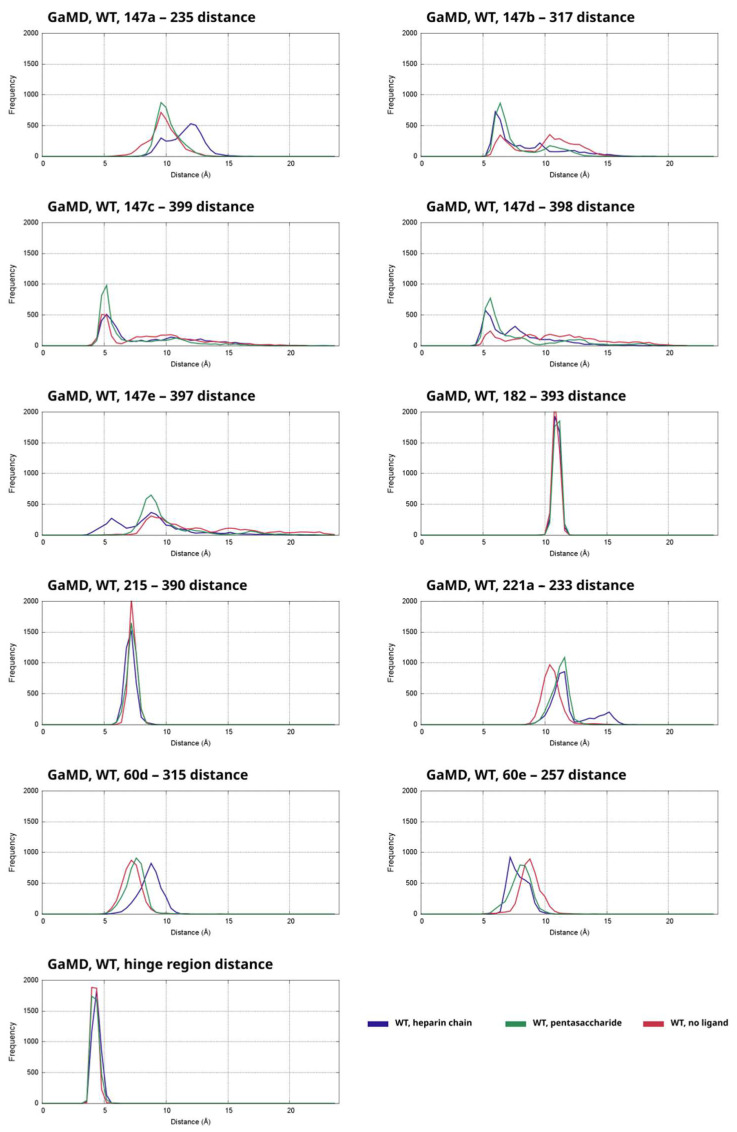
Distances between the alpha-carbon atoms of multiple residue pairs between AT and thrombin in the GaMD simulations of the wild type (WT) system, shown as histograms. The data from the simulations of the three types (heparin chain, pentasaccharide, no ligand) were combined and are plotted as separate histograms for each distance parameter separately. The frequency is the number of analyzed snapshots in each “bin” of the histogram, each curve corresponds to 4000 data points in total.

To study the effects of the presence of two types of heparinoid ligand on the exosite interactions, we have performed 500 ns GaMD simulations, four replicas for the three modeled activation states (heparin chain, pentasaccharide and no ligand). From the trajectories we calculated distances for several interacting residue pairs and compared the results to the results for the system with no ligand. The distribution of distances was depicted as histograms. For all distances, we have plotted the combined data from the four independent simulations for each activation state (heparin chain, pentasaccharide and no ligand) as separate histograms on a single plot. The frequency values correspond to the number of frames in one of the “bins” of the histogram. The results for the systems based on the 1TB6 X-ray structure with wild type AT are shown in Figure 3.

We have also plotted the distances between the involved amino acid pairs in each simulation as function of time, the results are shown in Figures S1–S11. The most important residues in thrombin are located in three different loops which are discussed separately. The 146–149 (γ) loop is one of the critical regions in thrombin that interacts with the exosite in AT. We have analyzed multiple distances between atom pairs in this region in the simulations of the WT system with a close “hinge”. The calculated distance parameters involving amino acids 147b and 147d as well as their interaction pairs in AT had a higher tendency for conformations with a larger distance value. In contrast, larger than average 147a distances occurred more frequently in the simulation with a heparin chain. Another important region playing a role in the interaction is the 60a–60f loop of thrombin. In this region, the Asp60e amino acid is typically located closer to Lys257 of AT in the simulations that contain a heparin chain than in the simulations with a ligand that cannot form a bridge.

We have also analyzed the conformation of the hinge region of AT in the simulations based on the X-ray structure, which is an important structural element known to undergo conformational change upon binding of heparinoids. However, the X-ray diffraction studies have shown that in a thrombin-AT Michaelis complex the optimal conformation is the closed state, which is otherwise a characteristic of the not activated state. To assess the stability of the “closed” conformation of this structural element in the simulations based on the X-ray structure, we have plotted the distance of the Glu380 amino acid in the hinge region from the alpha-carbon atom of Val375 (located in the neighboring strand of beta sheet A) as histograms (sixth row of Figure 3). It is evident that the conformation of the region in GaMD simulations is very stable, as indicated by the distance values between 4 and 5 Å in the large majority of analyzed frames.

### 2.3. Exosite Interactions in the Simulations of the Complexes Built by Docking

**Figure 4:**
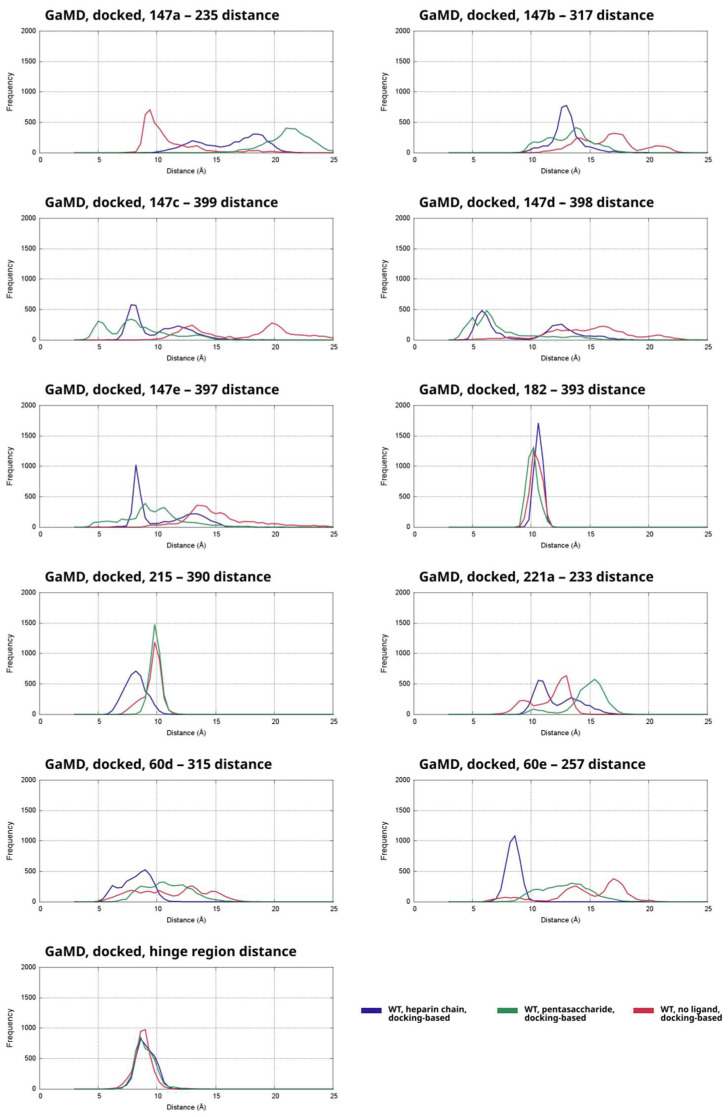
Distances between the alpha-carbon atoms of multiple residue pairs between AT and thrombin in the GaMD simulations of the Michaelis complex structure obtained by docking, shown as histograms. The data from the simulations of the three types (heparin chain, pentasaccharide, no ligand) were combined for each distance parameter. The frequency is the number of analyzed snapshots in each “bin” of the histogram, each curve corresponds to 4000 data points in total.

Using HADDOCK, we were able to build a starting structure for simulations in which AT had “open” hinge conformations and the most important contacts between the thrombin exosites and AT were present as well. To assess the strength of the interaction between the two proteins in such a state, we performed GaMD simulations for three types of the systems built from the docked structure (heparin chain, pentasaccharide, no ligand). To assess the stability between the most critical amino acid pairs involved in “exosite” contact, we analyzed the same interactions as in Figure 3 from the GaMD simulations. Similarly to Figure 3, the data is depicted as histograms (Figure 4). The distances as function of time were also plotted (Figures S12–S22).

In general, the simulations without a ligand or with a heparin chain or pentasaccharide exhibit considerably different conformational behavior. In the simulations based on the docked structure with an open hinge region but no ligand, several of the most important interactions are almost absent (Figure 4, Supplementary Figures S12–S22). The contacts between the 147a–148 loop and AT are drastically affected. This is indicated by the much larger distance values for thrombin residues 147a, 147b and 147c on the histograms compared to the simulations of the X-ray diffraction-based systems. Although a tendency for weaker contact between this loop and AT is evident even in the simulations with the “closed” hinge, the distribution of the relevant distance values shows peaks at extremely high values. In contrast, the binding of Trp147a amino acid was comparable to that in the simulations based on the 1TB6 structure with a closed “hinge”. These results can be explained by the fact that a state with hinge region expulsion but without a ligand is probably already unfavorable, and binding of thrombin to such a state of AT is even less likely. The interaction of the critical Arg221a amino acid with its interaction partner was, however, the least affected in the simulations without a pentasaccharide. In summary, the analysis results clearly show that the interactions with the exosites are much less favorable than the “open hinge” conformation of AT binds to thrombin.

Compared to the other two categories of simulations based on the docked structure, states with high values between the analyzed amino acid pairs was observed at the lowest frequency in the trajectories that contained a heparin chain “bridging” the two proteins. Importantly, the contacts formed by amino acids 60d, 60e, 147b, 147c and 215 (see the corresponding plots of Figure 3) were closer to the optimal value in the complexes with the longer heparinoid compared to the related pentasaccharide-containing and ligand-free systems. It was closer to the corresponding distances in the histograms of X-ray diffraction-based simulations as well. In contrast, the distances between the important Trp147a amino acid and its binding site were far higher than optimal in nearly all analyzed frames. This suggests that the presence of a heparin chain forming a bridge has a limited stabilizing effect on the complex.

In the simulations that were based on the docked structure but contained a pentasaccharide (shown as a separate curve on the plots of Figure 3), high distance values corresponding to a lack of favorable interactions were observed in the majority of the analyzed distance parameters. Most importantly, in the vast majority of analyzed frames, the critical 147a and 221a residues of thrombin were at a very large distance from their assumed interaction partner in AT, compared to the simulations with a closed “hinge”. Furthermore, the values for the parameters involving thrombin residues 60d, 60e were slightly lower than in those observed for the simulations with no ligand, but even these results indicate very weak binding.

In summary, the “open” state of the hinge region makes the “exosite” interaction between thrombin and AT considerably weaker. The hinge region probably has a significant effect on the conformation of the RCL of AT. As a result, the RCL conformations optimal for thrombin binding and “exosite” interactions may become unfavorable, while the typical RCL conformations in this state do not allow for favorable interactions between the exosites.

### 2.4. Exosite Interactions in the Michaelis Complexes with the AT-Resistant Thrombin Mutants

To study the consequences of three selected mutations involving the Arg221a amino acids known to cause impaired thrombin inactivation by AT, we performed molecular dynamics simulations for two types of complexes for each mutant: one containing a heparinoid and one without ligand. These simulations were based on starting conformations comparable to the X-ray diffraction structure. (The structure obtained by docking with an open “hinge” in AT was not used for simulating mutants because the observed weak exosite interactions in the GaMD simulations suggest that these are probably not the typical conformations of the system.)

The plots for each analyzed distance parameters contain histograms for the three mutations in the R221a position and the two simulated activation states (heparin chain, no ligand) separately (six histograms in total in one plot) (Figure 5 and Figures S23–S33). Each corresponds to the combined data from the two replicas. One curve in each plot represents 2000 analyzed frames in total.

In general, the presence or absence of a heparinoid molecule has similar effects on the distances in both the simulation of the WT system and the mutants. If the distance between one analyzed residue pair such as 147a in wild type thrombin and 317 in AT was typically lower in the simulations with a heparin chain than in those without a ligand, then the same tendency could be observed in the simulations of the mutant thrombins as well.

**Figure 5:**
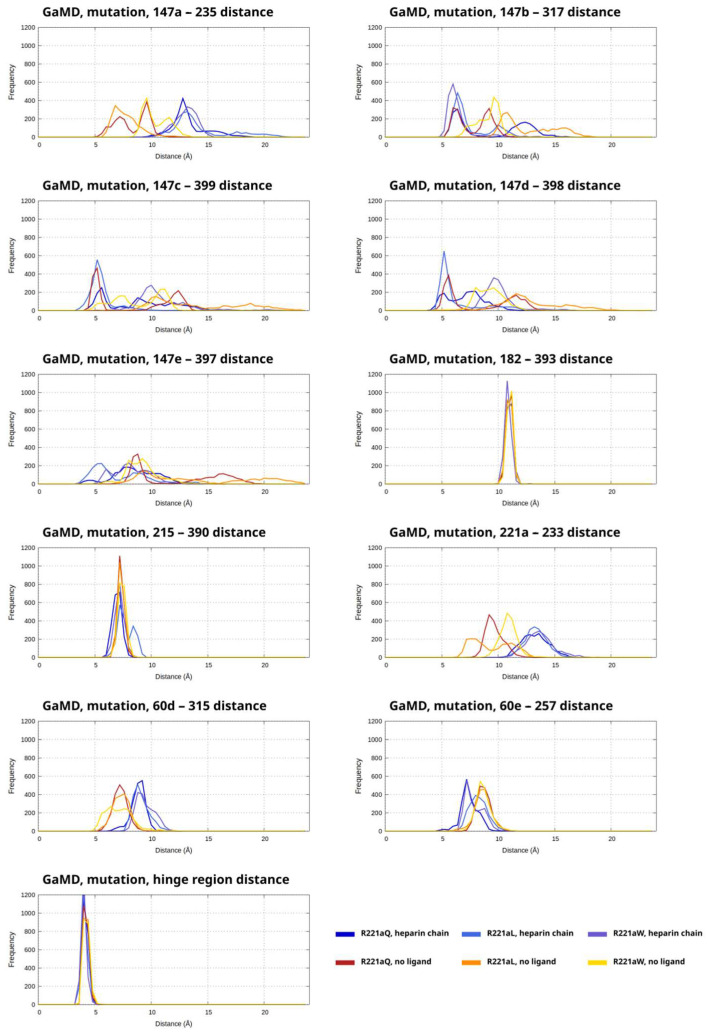
Distances between the alpha-carbon atoms of multiple residue pairs between AT and thrombin in the GaMD simulations of the thrombin mutants, shown as histograms. The data from the simulations of the two types of model systems (heparinoid chain, no ligand) were combined for each distance parameter, which are plotted as separate histograms. The curves for each mutant are shown in shades of blue (heparinoid) or in shades of orange or yellow (no ligand). The frequency is the number of analyzed snapshots in each “bin” of the histogram, each curve corresponds to 2000 data points in total.

Among the three simulations of the systems containing a heparin-like molecule, the highest distance values (corresponding to less favorable binding) could be observed in the R221aW simulations (Figure 5). Exceptions were the 147b–317 distance where the R221aQ mutant had the highest fraction of analyzed states with larger distance values and the 60e–257 distance where the R221aL mutant was the most affected. Regarding the simulations without the ligand, we could observe values for the analyzed parameters that were well above those found in WT simulations and this was most evident in the simulations of the mutation R221aL. This was most apparent for amino acids 147b–147e of thrombin and their amino acid pair in AT.

To assess the conformation of “hinge” region in AT from these simulations, we also analyzed the distance between amino acids 375 and 380. Similarly to the simulations of WT thrombin based on the X-ray structure, the analyzed parameter was in a narrow range between 4 and 5 Å in almost all frames. This indicates high conformational stability in the region and no tendency for transitioning into an “open” state.

### 2.5. Effect on Arg596 Mutations on the Exosite Interactions

The Arg221a (Arg596) amino acid of thrombin affected by the Yukuhashi, Belgrade and Padua II mutations is part of an interaction site that normally binds a Na^+^ ion (along with another important residue, Lys224). In the X-ray structure published by Li et al., it interacts with the Glu232 and Asn233 amino acids of AT [6].

First, we have analyzed the effect of the presence of a heparin chain or a pentasaccharide on the presence or absence of exosite interactions between AT and the catalytic domain of WT thrombin. Supplementary Figure S34 shows the frames in the simulation as the function of time in which the distance was below 5 Å for the simulations containing WT thrombin. The figure is colored by “groups” of residues in the AT protein that are located close to each other (in other words, part of the same exosite). In the analysis, we mainly included salt bridges and other polar interactions; however, the contacts between a few important pairs of polar residues were also analyzed.

The interaction of the Y60a and the K60f residues with the reactive center loop amino acids 391–394 is relatively stable in all analyzed simulations. A significant difference between the simulations containing a full heparin chain from those with a pentasaccharide or no ligand was the contribution of D60a amino acid to the binding. The salt bridge between it and K226 in AT was only present in the former group of simulations. In the simulations with a heparin pentasaccharide or no heparinoid at all, this amino acid interacts with Lys257 only. The W147a amino acid formed contacts in three of the four simulations containing a heparin chain with Tyr253 of AT, an important exosite residue. However, there was almost no interaction between these residues in the remaining two types of WT systems (pentasaccharide, no ligand). Its interactions with Asn233 and His319, located in the same region in AT, were less affected by the absence of a relatively long heparinoid chain. In contrast, interaction between the W60d amino acid of thrombin and the N-terminal end of the reactive center loop was mainly observed in the simulations containing only a pentasaccharide.

In Figure S35, the data for the presence or absence of interactions below 5 Å is shown for the three thrombin mutants in the simulations with or without a heparinoid ligand. From the data, it is evident that several interactions become weaker between the two proteins as a consequence of the mutations. Compared to the corresponding simulations of the WT system, the three mutants had a particularly large effect on the interactions formed by the 146–147e loop of thrombin in the presence of a heparin chain “bridge”. Almost no contact was detected between the E146 amino acid in thrombin and R235 in AT. The contact between T147 in thrombin and Y253 in AT was also almost completely absent. The interactions involving W147a in thrombin were also affected considerably by the mutants.

We have also performed this analysis on the simulations of mutant thrombin systems containing no heparinoid. In all of the analyzed simulations, one of the most critical contacts between the two proteins was absent; no interaction was detected between the important R221a residue of thrombin and its binding site near Asn233 in AT. Among the three simulations, the R221aW was somewhat less affected than the other two systems. The results indicate that the mutants probably have a smaller effect on the state without a heparinoid compared to the ternary (AT–thrombin–heparinoid) complexes.

We performed the analysis on the simulations based on the docked structures as well. Figure S36 contains plots of the presence or absence of the interactions plotted as a function of time. In all of the simulations, only a fraction of the interactions between the exosites of the two interacting proteins was present, compared to the simulations based on the X-ray structure. In nearly all trajectories, we were not able to detect binding between the important Arg221a amino acid in thrombin and its binding site near Asn233 in AT. One of the few interactions still present in most simulations was R173–E232. Based on the absence of the majority of “exosite” interactions, we can conclude that AT–thrombin complexes with an open “hinge” conformation in AT are likely less stable than in the states corresponding to the X-ray structure (closed “hinge”).

### 2.6. Root Mean Fluctuation Analysis of the Complexes with Wild Type or Mutated Thrombin

Root mean square fluctuations are a frequently used metric for the flexibility of the protein backbone at each amino acid in a peptide chain. In our previous work, we have used this method successfully for analyzing the effects of ligand binding on distant regions of the proteins such as AT. We were also able to study whether the ligands have an allosteric effect on the factor (or amino acids that the factor does not directly interact with in the case of the long heparin chain).

**Figure 6:**
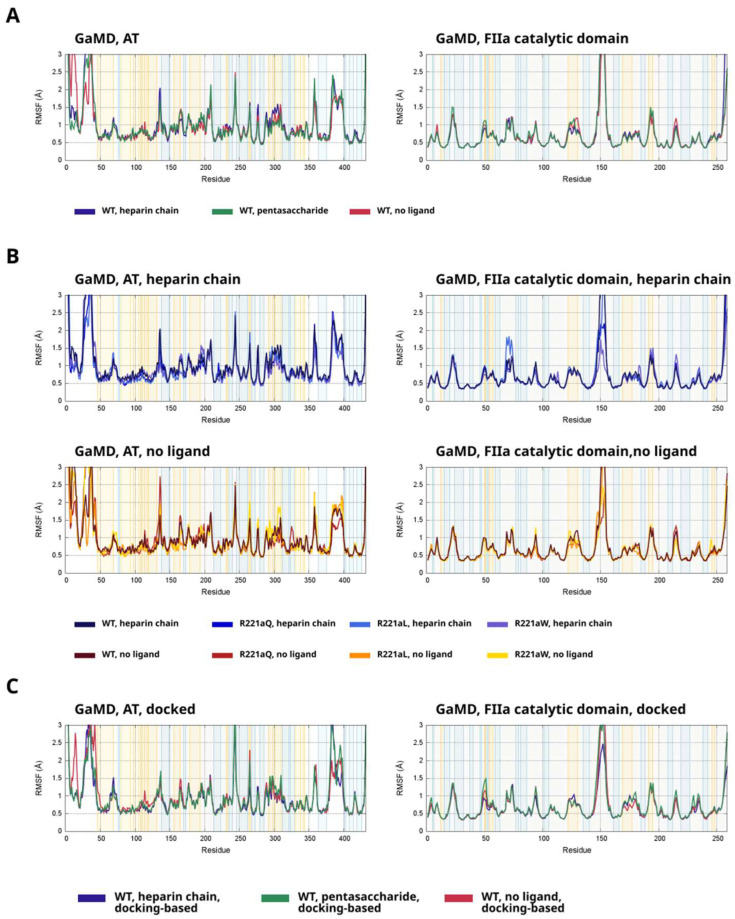
(**A**) Root mean square fluctuations (RMSF) of AT and the catalytic domain of thrombin in the GaMD simulations of the WT systems. The figure shows averaged data from 4 simulations based on the same model systems (heparin chain, pentasaccharide, no ligand). Residues in thrombin are numbered from the N-terminus and do not follow the chymotrypsin numbering. (**B**) Root mean square fluctuations (RMSF) of AT and the catalytic domain of thrombin in the GaMD simulations of the R221a mutants, compared to the WT systems. The RMSF for the mutants is an average of the data from two independent simulations. (**C**) The results of RMSF analysis for the simulations based on the docked structure.

Using RMSF analysis on the WT simulations, we were able to identify regions whose conformation is affected by the presence or absence of pentasaccharide ligands (Figure 6A, Figures S37 and S38). First, we detected differences in two important regions among the three types of simulated model systems 230–240 and 300–320 which are located close to the exosites. This suggests that the presence of a longer heparin chain has allosteric effects on this region, but this is less significant with the system where only a pentasaccharide is present. Second, the reactive center loop showed less extensive fluctuations in the simulations of the system with a pentasaccharide ligand compared to the other two types of model systems.

In the catalytic domain of thrombin, the conformational behavior in the simulations without a ligand was different from the other two types of simulations mainly in two regions: a helix at amino acids 125–130 and at the 203–206 loop. In the simulations containing a heparinoid molecule, the regions close to the binding site exhibited lower fluctuations in the simulations of all three mutants compared to the WT.

To assess the effects of the mutants on the fluctuations of AT or the catalytic domain FIIa, we compared the averaged fluctuations from the two replicas to the data from the relevant four simulations of the WT system. In Figure 6B (and Figures S39 and S40), the data is shown for the systems containing a heparin chain and no ligand, respectively. In general, the three mutants showed similar conformational behavior to each other, which was different than in the WT simulations. The most important regions where we could detect differences were at amino acids 80–120 (close to heparin binding site), 320–320 (close to the thrombin binding sites outside the RCL) and the RCL itself (amino acids 380–398). Thrombin with the R221aQ mutation exhibited significantly different conformational behavior in the 70–78 region from both the wild type protein and the mutants. This loop is not located close to the binding site, but does form contact with amino acids of the γ-loop, indirectly affecting its conformation. However, this was not observed in the simulations without a pentasaccharide.

To assess the flexibility of important regions in the docking-based simulations and to investigate the consequences of heparinoid ligand binding, we performed RMSF analysis on the simulations based on the docked structure as well. We performed this analysis for both AT and thrombin. The results are presented on Figure 6C and on Figures S41 and S42.

In AT, the binding of heparinoid ligands has an effect on the conformation of amino acids in region 110–125 which contains multiple residues involved in the heparin binding. In the simulations with a heparin chain or a pentasaccharide, lower values were observed than in those without a ligand, indicating that the heparin binding site was more stable in the presence of a ligand. The results also show significant differences between the fluctuations of the RCL among the trajectories for the three types of model systems. However, the flexibility of this loop can be both affected allosterically by heparinoid binding or the interaction with thrombin. Higher values in the simulations with pentasaccharide possibly indicate such an allosteric effect combined with a less favorable AT–thrombin interaction, compared to the system with a longer heparin chain. In thrombin, the conformations of two important surface loops close to the binding sites, around amino acids 50 and 170, exhibited different conformational behavior between the three types of systems.

### 2.7. Allosteric Processes in the Complexes Based on Generalized Correlation Analysis

Detection of correlated motions at protein backbone between two amino acids can help elucidate which regions in the protein are part of allosteric pathways. We have used the “generalized correlation” method developed by Lange and Grubmüller for this analysis, similarly to our previous work [24,25]. We have performed this analysis on the GaMD simulations of both the wild type protein and the mutants. The results are presented in Supplementary Figure S43.

In the plots for the analysis of the four simulations of WT thrombin with a heparin chain, it is evident that the correlations were most significant inside thrombin or AT; however, there are also signs of such processes between the two protein components of the system. The correlations within AT were also different than in the system with a longer heparin chain. A larger region in AT (470–500) showed only very weak correlated motions with both other parts of AT and thrombin. The correlated motions inside the two protein subunits were less affected, although the patterns were considerably different than those detected in the simulations with a pentasaccharide.

### 2.8. Cluster Analysis

We have performed cluster analysis on the trajectories of both the wild type and mutant system to identify conformational types present in the simulations. The cluster analysis of the WT simulations was performed on all three types of the systems (heparin chain, pentasaccharide, no ligand) at the same time. The analysis for the mutants included the systems containing the three mutations, as well as the two activation states (heparin chain, no ligand) we have modeled. For clustering, we have used the K-means algorithm. The clusters were numbered by the software by the fraction of frames belonging to them; the numbers do not imply any similarity between the detected conformational types.

**Figure 7:**
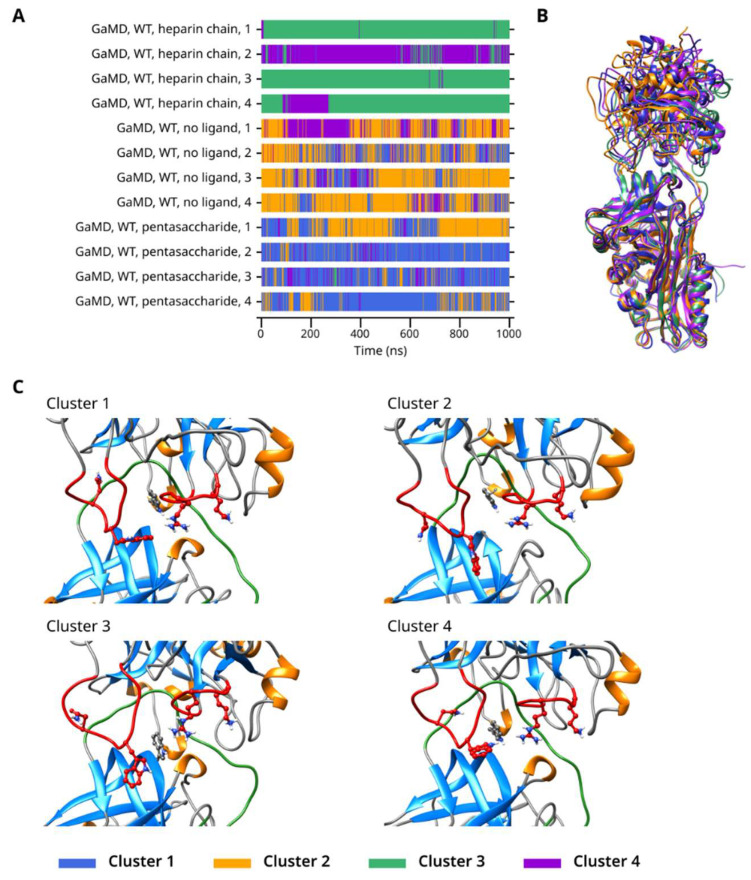
(**A**) Cluster number as a function of time in the GaMD simulations of WT thrombin complexed with AT. (**B**) Superposition of the representative frames from each cluster. (**C**) The interaction between the exosites of thrombin and AT in the four clusters.

First, we have performed an analysis where the data from the four simulations for each activation state (heparin chain, pentasaccharide, no ligand) of the wild type system were combined, which resulted in four clusters. The results are shown in Figure 7. In the simulations with a heparin chain, most conformations belonged to either Cluster 3 or 4. In contrast, in the trajectories for the systems with a heparin pentasaccharide or no ligand at all, Clusters 1 and 2 were predominant, with Cluster 2 more common in the trajectories with no ligand and Cluster 1 in the pentasaccharide-containing ones. Cluster 4 was an exception. It was the predominant cluster in one of the simulations with a heparin chain, but was also present in the trajectories without the longer heparin chain. Rapid changes between conformations belonging to different clusters could be observed in the systems with either a pentasaccharide or no ligand, suggesting significant conformational flexibility. In contrast, such changes happened considerably less frequently when the heparin chain capable of forming a bridge was present.

**Figure 8:**
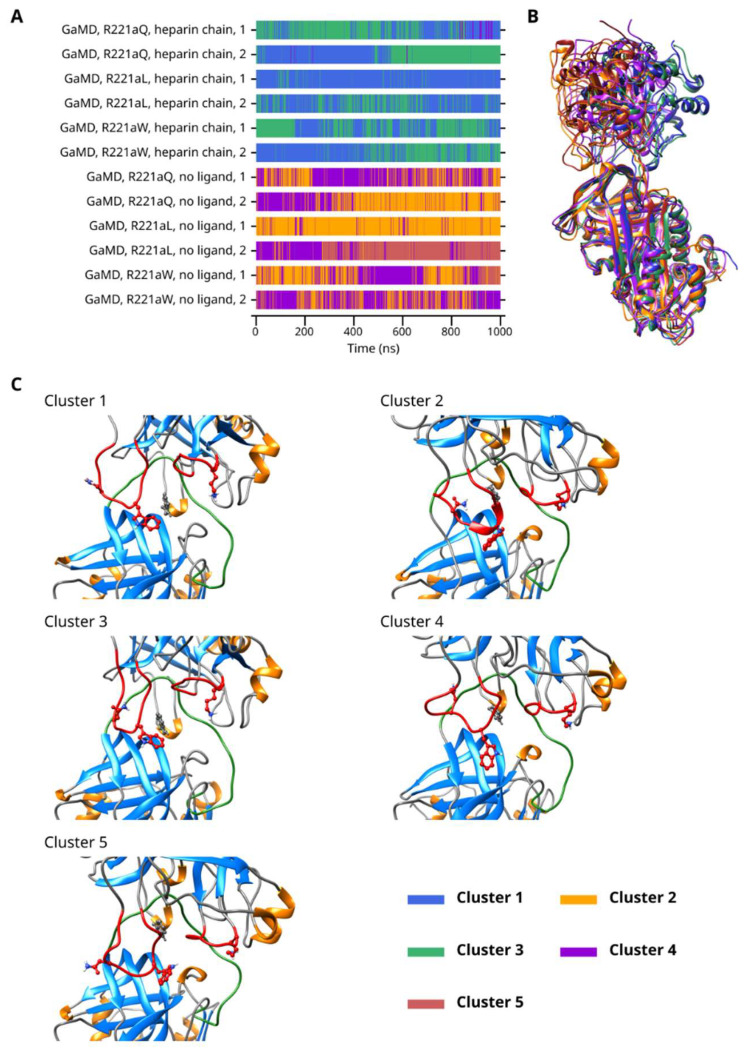
(**A**) Cluster number as a function of time in the GaMD simulations of thrombin mutants complexed with AT. (**B**) Superposition of the representative frames from each cluster. (**C**) The interaction between the exosites of thrombin and AT in the five clusters.

For the GaMD simulations of the three Arg221a mutants, five clusters were created. The analysis (Figure 8) included all three mutants in both simulated activation states (a total of 12 simulations with the two replicas included in all cases.) There was significant difference between the behavior of the systems with a heparinoid or no ligand. Rapid conformational transitions were observed between Clusters 1 and 3 in the heparin-chain-containing simulations and between Clusters 2 and 4 in the simulations with no ligand. Cluster 5 was mainly present in the simulations of the R221aL and R221aW mutants without a ligand. Interestingly, conformations belonging to Cluster 4 were also observed in trajectories with a heparinoid molecule at a low frequency, mainly in the case of the R221aQ mutant.

We also performed a cluster analysis using a similar protocol on the simulations based on the structures obtained by docking (with an open “hinge” conformation) as well. The results are presented in Supplementary Figure S44. In the simulations containing a heparin pentasaccharide, Clusters 1 and 4 were the most prevalent. In the systems without a ligand, mainly Clusters 1 and 3 were observed, while Clusters 2 and 5 were mostly present in the simulations based on the docked structure with an added pentasaccharide. Conformational transitions between two clusters were observed in only some simulations; in others, there was one “dominant” conformational type. It is also evident (part B of Figure S44) that the conformational types found by cluster analysis were vastly different from each other regarding the position of thrombin compared to AT. The representative frames for each cluster indicate vast differences in the conformation of the γ-loop among the observed conformational types. This may indicate that due to significant changes in the relative positions between the two proteins in the docking-based simulations compared to the X-ray structure, this loop cannot adapt its optimal conformation in multiple clusters. Interestingly, in Clusters 2 and 5, mainly found in the pentasaccharide-containing systems, the loop was shifted towards amino acid Gln268 in AT, about 9–10 Å away from the binding site in the X-ray structure. In Cluster 3, it had a tendency for forming a helix-like structure, resulting in an orientation of the Trp147a amino acid that is not suitable for binding. Furthermore, the critical Arg221a amino acid was not able to bind to its optimal binding site near amino acid Asn233 in Clusters 2, 4 and 5. This data further supports the conclusion that the AT–thrombin Michaelis complexes with an open “hinge” conformation of AT may have limited physiological significance.

### 2.9. Binding of the Heparin Chains to the Two Interacting Proteins and the Heparin Pentasaccharide to AT

Heparin chains that are long enough to form a bridge between thrombin and AT significantly improve the inhibition of thrombin. For comparison, we have also simulated systems containing a pentasaccharide which can interact with AT only. (The mutants were not modeled with a pentasaccharide.) To assess whether the mutations have a significant effect on the ligand binding, we compared the binding sites of the ligands on either AT or thrombin to their position in the X-ray diffraction structure, or the starting structure used for the pentasaccharide-containing system. The conformational differences were expressed as RMSD. For the heparin chains, the interactions with both thrombin and AT were analyzed.

**Figure 9:**
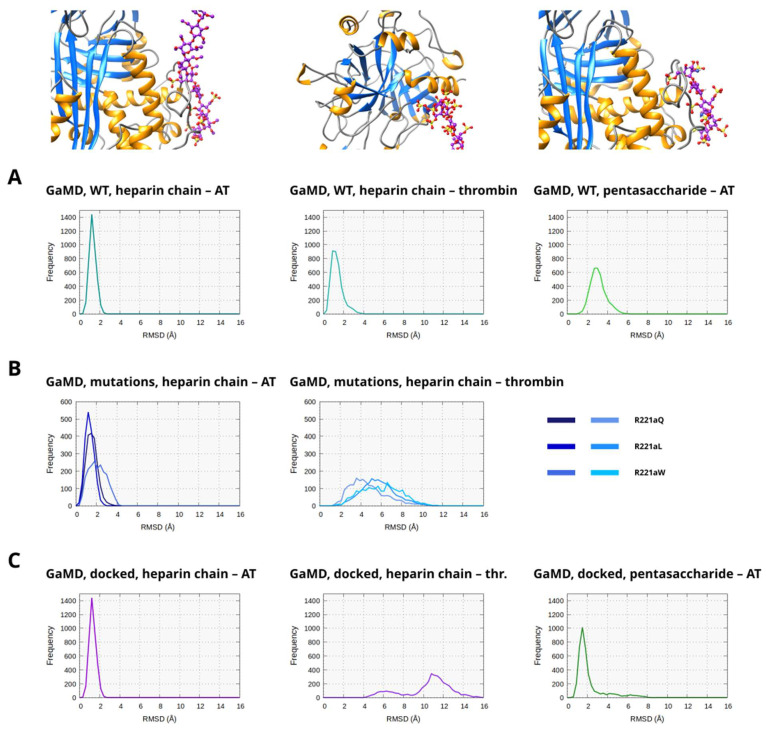
Analysis of the interactions of the heparinoid molecules with their binding sites either in AT or thrombin. The difference in the position of the ligands compared to the initial conformations was expressed using RMSD. For the simulations containing a heparin chain, the binding to both AT and thrombin were analyzed separately. In the figure, the results are shown separately for the simulations containing a WT system (4000 analyzed frames). (**A**) The simulations of the three mutants, with the data combined to two histograms (2000 analyzed frames) (**B**) (systems with a pentasaccharide were not simulated in this case) and finally, the simulations based on the docked structures (4000 analyzed frames). (**C**) The frequency is the number of analyzed snapshots in each “bin” of the histogram.

In the simulations based on the X-ray structure and containing WT thrombin (Figure 9A and Figure S45), the binding of the heparin chain was stable to both proteins, and its positions were close to their positions in the X-ray structure, as suggested by the RMSD values below 3 Å. In the WT systems simulated with a pentasaccharide, somewhat higher RMSD was detected in a fraction of the frames, indicating that fondaparinux interacts somewhat less favorably with AT than a heparin chain in a Michaelis complex with thrombin.

In the simulations of the three R221a mutants (Figure 9B and Figure S46), the interaction between the heparinoid and AT remained stable. However, the RMSD values in the R221aW system were somewhat higher. This can be explained by the significant movement of thrombin in these complexes compared to the X-ray structure, which probably results in some level of conformational strain at the other end of the molecule. In the analysis of the heparin chain binding to thrombin, we could detect increased RMSD values compared to the WT simulations. The values were typically higher in the simulations of the R221aW and R221aQ mutants compared to R221aL.

In the simulations based on docking (Figure 9C and Figure S47), both types of ligands (heparin chain, pentasaccharide) interacted favorably with the binding site of AT. In contrast, very large RMSD values were observed for the interaction of the heparin chain with the heparin binding exosite of thrombin. This indicates that the “open” hinge region conformations have a large effect on how thrombin can bind to AT, and as a consequence, the interaction of the molecule with both proteins at the same time becomes less favorable.

## 3. Discussion

Although the mechanism of thrombin inactivation by AT was a subject of several studies, some unanswered questions about the interaction of the two proteins at the molecular level still remain. An X-ray structure was only available for ternary Michaelis complexes with a heparin-like molecule. No experimentally determined, atomic-level structure exists that could explain how the two proteins interact when no heparinoid is present or why the binding of a pentasaccharide (too small for a ternary complex) has only a very limited effect on the processes in contrast to FXa and FIXa. Also, it was not completely understood why a “closed” hinge region conformation of AT is favored despite the presence of a heparinoid.

We modeled multiple types of systems in the present work. First, based on the X-ray structure of the complex, we performed simulations for complexes with two types of ligands (heparin chain or pentasaccharide), as well as no ligand. Second, we built a model system using docking that contained an “open” conformation for the “hinge” region and, based on it, we modeled the same three types of systems (two types of heparinoids plus no ligand). Third, we selected three mutations involving Arg221a in thrombin known to cause AT resistance, a type of thrombophilia. (Prothrombin Yukuhashi (Arg221aLeu), Belgrade (Arg22aGln), Padua 2 (Arg221aTrp)). The model systems for mutants were based on the X-ray structure rather than the docked complex.

Furthermore, we have built an AT–thrombin complex structure (the protocol is described in Section 4.3) using docking that contained an open “hinge” region, in AT, with the conformation of AT based on the 1E03 X-ray diffraction structure. Using GaMD simulations based on this structure, we were able to study the role of the important “hinge” region of AT in the AT–thrombin interaction. The trajectories showed that multiple important interactions become unfavorable in such a state, indicated by a large increase in the distance values between the amino acid pairs. The significant conformational changes in the γ-loop of thrombin suggest that conformations similar to those found in the X-ray structure cannot bind favorably to AT. This is a large significant difference from the AT–FXa and AT–FIXa complexes, where the “open” conformation is the requirement of favorable interaction. Our findings probably indicate a lower biological relevance of the open “hinge” state in the case of thrombin inhibition. This was also the reason why we selected the X-ray diffraction structure rather than the docked structure as the basis for simulating the AT-resistant thrombin mutants.

Our simulations serve as an atomic level model for better understanding the process in the absence of heparinoids forming bridges. Using cluster analysis, we have detected several relevant conformational types in both the presence and the absence of heparinoids. This suggested the significant conformational flexibility of the systems, especially when there was no “bridging” heparin chain present.

We have also modeled another form of the Michaelis complex in which antithrombin was complexed with a high-affinity pentasaccharide. Such molecules are known to enhance the FXa and FIXa inactivation rates of AT by several orders of magnitude, yet the presence of such a molecule has only a very modest effect on thrombin inhibition. Cluster analysis from the results of our simulations have revealed that the characteristic conformational types in such a state have similarities to those found in the systems simulated without any ligand, but were considerably different from what we have observed for complexes with a “bridging” heparinoid chain. This suggests that the binding remains weak even in the presence of a heparin chain; such a molecule cannot compensate for the defects in the interactions between the exosites of the two proteins.

Mutations in thrombin located in one of the exosites are known to cause a rare but very severe type of thrombophilia. The most studied mutations affect the Arg221a (in chymotrypsin numbering) residue. Compared to a previous study involving docking [20], our molecular dynamics simulations can serve as an improved in silico model for understanding the consequences of three selected mutants at an atomic level: Prothrombin Yukuhashi (Arg221aLeu, p.Arg596Leu), Belgrade (Arg221aGln, p.Arg596Gln), Padua 2 (Arg221aTrp, p.Arg596Trp). By analyzing our simulations, we could find amino acid pairs that could no longer contribute to the interaction as the consequence of the mutations that otherwise play a role in the AT–thrombin complex with WT thrombin. An important observation was that the presence of a heparin chain cannot compensate for the defects of interactions between the exosite; the binding between the two proteins is at least as weak as in the absence of the ligand. Studying these mutants could also provide insights into the effectiveness of AT-dependent anticoagulants in the affected patients.

Our studies have some limitations. Although we aimed for a detailed study of the conformational behavior of the AT–thrombin Michaelis complex, the sampling could have been insufficient, as we may have missed some relevant conformations using our current simulation protocol. Also, we could not observe a significant level of unbinding between the two proteins of the complex in any of the simulations, so we could not draw any conclusion on the mechanism of unbinding or binding. The conformational sampling might be improved even further by using a newer enhanced sampling MD technique such as PPI-GaMD [32] or simulating multiple docked structures obtained using different protocols. Another limitation of our study is that due to the extent of conformational changes within the simulations’ multiple activation states of AT or in thrombin mutants, it is difficult to assess the differences quantitatively using metrics such as free energies due to the high fluctuations. Despite the qualitative nature of our data, we still expect that our simulations can still serve as a model for interpreting experimental data.

In summary, our molecular dynamics simulation revealed several novel aspects of the molecular mechanism of AT–thrombin interactions, in addition to previously available X-ray crystallography and biochemical data. Most importantly, our results have revealed the loss of interactions between amino acid pairs and identified conformational changes as possible reasons behind less favorable interactions in systems not containing a stabilizing heparin chain or having an “open” hinge conformation. Furthermore, our simulations provide a detailed and complex picture about the mechanisms behind the Arg221a mutations, and why they are causing impaired thrombin inhibition by AT. By revealing the differences at an atomic level between thrombin mutants and WT structures, the major consequence of the mutations—due to altered conformations—might be a weaker interaction between thrombin and AT.

## 4. Materials and Methods

### 4.1. Preparation of Model Systems from the X-Ray Diffraction Structure

To study the interaction of AT with wild type thrombin, we built three types of model systems. All of the structures were based on the 1TB6 X-ray structure of AT–thrombin–heparin chain ternary complex [6]. The first was based on the X-ray structure of the AT, which contains a heparin-like molecule that formed a bridge between the two proteins. The heparin mimetic we used was SR123781, the molecule found in the X-ray structure. The second model contained a high-affinity pentasaccharide molecule, fondaparinux. The pentasaccharide was added to the system by root mean square (RMS) fitting of the 3EVJ X-ray diffraction structure [14]—which contains AT complexed with this molecule—on the AT protein in the 1TB6 complex structure. The third type of model lacked any heparinoid.

To study the consequences of amino acid changes in position 596 (221a according to chymotrypsin numbering) on the binding, we also investigated three mutants, Arg221aGln, Arg221aLeu and Arg221aTrp. For the simulations of the mutants, we have assumed that conformations with a closed “hinge” are the stable form, and therefore we have built these structures based on the X-ray structure. We simulated two types of systems for them, one with the “long” heparin mimetic and one with no heparinoid; we did not build a fondaparinux-containing model in this case. Otherwise, we followed the same system preparation protocol as for the wild type systems.

For the simulation of the protein, we have chosen the AMBER 14SB force field [33] and we used GAFF 1.8 parameters for the heparin pentasaccharide (fondaparinux) and the heparin chain ligands [34]. We have selected this force field based on our previous work with AT–pentasaccharide complexes, the parameters for the pentasaccharide were identical to those we previously developed for the same molecule [25]. To parameterize the SR123781 molecule, we have followed a similar approach. We generated the GAFF 1 parameters using the Antechamber program and the charge parameters for the atoms were computed using the RESP method [35,36] implemented in the ANTECHAMBER program of AmberTools 22 (AmberTools 2022, University of California, San Francisco, CA, USA) [37]. The electrostatic potentials for RESP were computed using Gaussian 16 at the HF/6-31G* level [38].

The systems were solvated with TIP3P solvent molecules in an octahedral box [39]. The size of the box was calculated to have at least 10 Å space between its edges and the atoms of protein or ligand. We added Na^+^ and Cl^−^ ions to all simulated systems to model an ionic strength close to 0.15 M.

### 4.2. Molecular Dynamics Simulations

We used the Amber 22 software package (Amber 2022, University of California, San Francisco, CA, USA) [37] for molecular dynamics (MD) simulation. Before the production simulations of the models based on the 1TB6 X-ray structure, we performed energy minimization and equilibration on all simulated systems.

The energy minimization consisted of two steps. The first phase was 2000 steps of minimization using the steepest descent method in which we have applied weak position restraints on all atoms of the protein and the ligand (if it was present) to eliminate unfavorable interactions between the protein and the solvent. The second phase was performed with the restraints removed and consisted of 500 steps of steepest descent and then 1500 steps of conjugate gradients minimization.

After minimization, each system was heated to 310 K in a 2 ns simulation, with the non-solvent atoms restrained. This was followed by a 2 ns long pressure equilibration using the Berendsen barostat [40,41], with similar restraints. Before production MD, we performed 150 ns long equilibrium MD simulations in all systems studied.

The production simulations were performed at 310 K under NPT condition. The integration timestep was 2 fs. For temperature regulation, we have used the Langevin thermostat with a time constant of 2 ns^−1^. The systems were simulated at a constant 1 bar pressure using the Monte Carlo barostat. The Coulomb and the van der Waals cutoffs were set to 10 Å. Long-range electrostatic interactions were taken into account using the PME method [42].

To perform sampling of the conformational space of the protein complexes, we have used an enhanced sampling molecular dynamics method, GaMD [28,43]. We have already applied this method successfully in our previous work related to AT-factor Xa and AT-factor IXa complexes [29]. The GaMD equilibration phase was 100 ns long. In the first 10 ns, no acceleration was applied to the system, and the data collection started after 5 ns in this phase. Then the system was simulated for 90 ns with the GaMD potential. The GaMD parameters were updated at a frequency of 200,000 MD steps in this second phase, with the exception of the first 5 ns.

The production GaMD simulations for each wild type and mutated systems were 500 ns long. We performed four independent simulations for wild type AT in all three activation states, with a long heparin-like chain or fondaparinux and without heparinoid ligand (12 simulations in total). For the three thrombin mutants, two simulations were run for both states (with or without heparin chain; 12 further simulations).

### 4.3. Construction of Antithrombin–Thrombin Complex Models with an Open “Hinge” Region Conformation Using Protein Docking

As no X-ray diffraction structure for AT–thrombin complex is available with an open hinge region conformation, we have used protein–protein docking to build the initial structure. To achieve this, a suitable starting structure of AT was required. Most importantly, the RCL and the critical Arg393 residue in it must be in a conformation suitable for interaction with the catalytic domain of thrombin. Such an initial structure for the docking was obtained from a GaMD simulation of AT based on X-ray diffraction structure 1E03, which contains a partially open “hinge” conformation. This simulation was 300 ns long, but otherwise we have applied an identical protocol to the other GaMD simulations in the project. The best conformation for docking was selected based on the distance between the Arg393 side chain in the RCL from residue Tyr253 (an important amino acid of the “exosite”). The AT structure used in docking contained no heparinoid.

The structure for thrombin was derived from the 1TB6 [6] X-ray structure which was used for the other simulations in this study. Docking was performed using HADDOCK 2.4 [44,45] which is available as a web-based service. (https://rascar.science.uu.nl/haddock2.4, accessed on 17 April 2025) In one of our previous articles, we have successfully used HADDOCK for building AT–FXa and AT–FIXa complexes [29]. The main advantage of HADDOCK is that it allows defining multiple types of “restraints”, for example amino acids that are known to be involved in the protein–protein interactions. In order to build a complex structure where the important Arg393 residue of AT was positioned correctly, we defined two “unambiguous interaction restraints”: first, between the catalytic residue 195 of thrombin and the peptide backbone at the cleaved 393–394 bond and second, the positively charged side chain of Arg393 in AT and its binding pocket at amino acid Asp189 in thrombin. Additionally, we have defined further residues as “ambiguous” restraints: 229, 231–238, 249–250, 253–257, 319, 385–403 in AT and 39–41, 57, 60C–60F, 96–97A, 99, 147–149, 171–175, 190, 193, 195, 215–225 in thrombin. Such “restraints” are taken into account by HADDOCK in a way that only a fraction of them needs to be present in the final structure. The structure with the highest score from the best ranked cluster was selected for further simulations.

The structure produced using docking did not contain a pentasaccharide or a heparin chain. The fondaparinux ligand was re-added by alignment of the structure obtained by docking with the initial pentasaccharide-bound complex structure. However, the relative positions of the two proteins in the docked complex differed significantly from that found in the 1TB6 structure; therefore, we followed a protocol with multiple steps to build the ternary complex.

First, we built an initial structure in which the heparinoid molecule was only bound to AT using the same method we used for the pentasaccharide complex. Later, we performed energy minimization and a 250 ns long equilibrium MD simulation. It corresponded to the 150 ns equilibration step of the system preparation. Otherwise, the protocol for system equilibration and production GaMD was identical to that we used for the simulations of the systems based on the 1TB6 structure. We have performed four independent 500 ns GaMD simulations for all three types of model systems: heparin chain, pentasaccharide and no ligand.

### 4.4. Trajectory Analysis

We performed trajectory analysis, including calculation of distances, RMSF and cluster analysis using the CPPTRAJ program of AMBER 22 (AmberTools 2022, University of California, San Francisco, CA, USA) [46].A total of 1000 snapshots from each 500 ns production GaMD simulation were included in the distance analysis, and 500 in the cluster analysis. For cluster analysis, we have used the K-means algorithm available in CPPTRAJ. “Generalized correlations” between the movements of alpha-carbon atoms were computed using a method proposed by Lange and Grubmüller [47].

## Data Availability

The original contributions presented in this study are included in the article and Supplementary Material. Further inquiries can be directed to the corresponding authors.

## Supplementary Materials

The supporting information can be downloaded at: https://www.mdpi.com/article/10.3390/ijms26209901/s1.

## Abbreviations

The following abbreviations are used in this manuscript:

AT	Antithrombin
FIIa	Thrombin
FIXa	Activated coagulation factor IX
FXa	Activated coagulation factor X
GaMD	Gaussian Accelerated Molecular Dynamics
MD	Molecular dynamics
RCL	Reactive center loop (of AT)
RMSD	Root mean square deviation
RMSF	Root mean square fluctuations
WT	Wild type
