# Supplementary material for: The Effects of Heparin Binding and Arg596 Mutations on the Conformation of Thrombin–Antithrombin Michaelis Complex, Revealed by Enhanced Sampling Molecular Dynamics Simulations
Source: Int J Mol Sci. 2025 Oct 11;26(20):9901. doi: 10.3390/ijms26209901 (PMC12564035; doi:10.3390/ijms26209901)
Supplement: Supplementary file 1 [file ijms-26-09901-s001.zip › ijms-3911877-supplementary.pdf]

The Effects of Heparin Binding and Arg596 Mutations on the Conformation of Thrombin–Antithrombin Michaelis Complex, Revealed by Enhanced Sampling Molecular Dynamics Simulations

Gábor Balogh and Zsuzsanna Bereczky

Division of Clinical Laboratory Science, Department of Laboratory Medicine, Faculty of Medicine, University of Debrecen, H-4032 Debrecen, Hungary

* Correspondence: balogh.gabor@med.unideb.hu (G.B.); zsbereczky@med.unideb.hu (Z.B.)

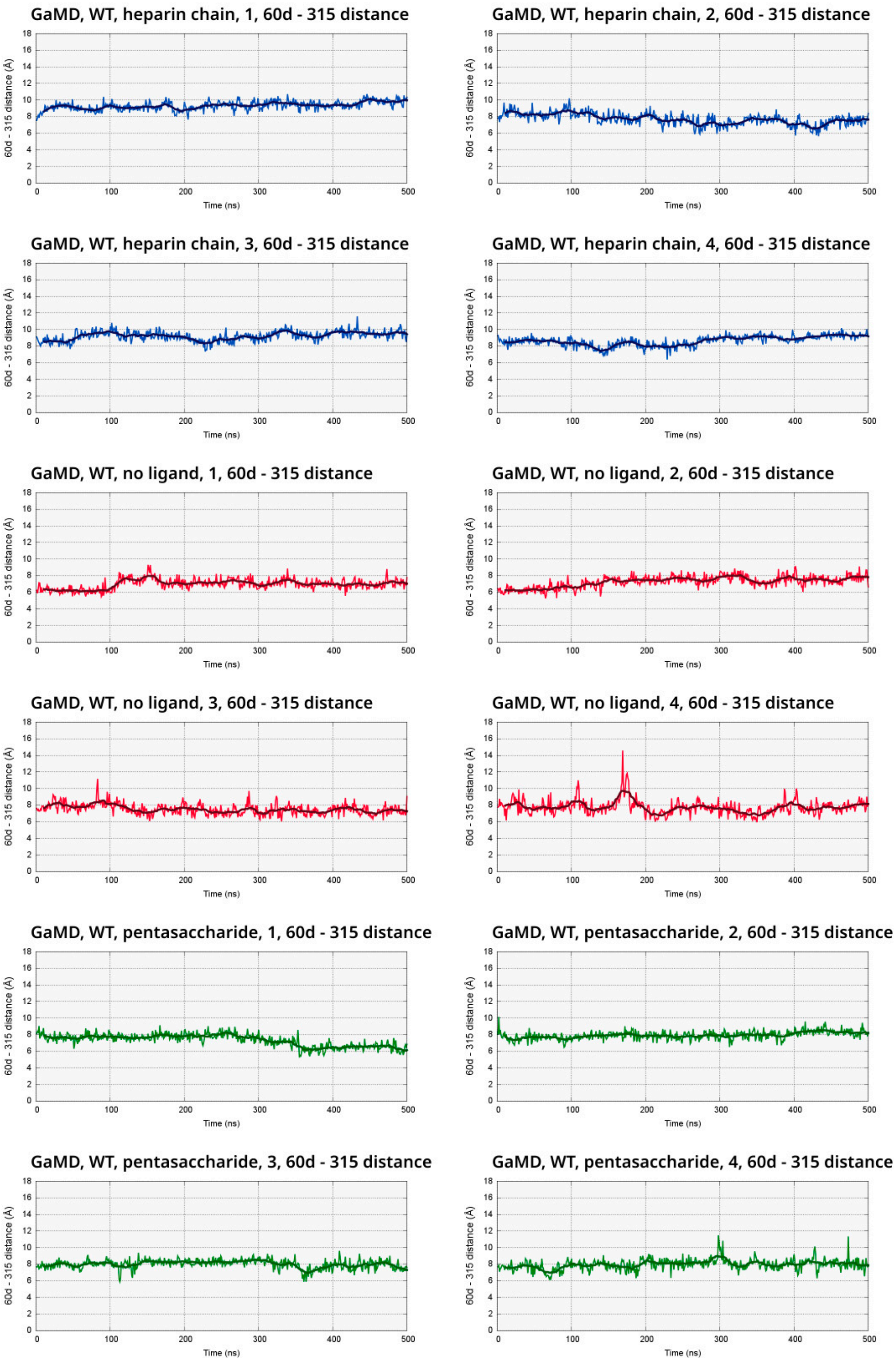

Figure S1. Distance between the alpha carbon atoms of amino acid 60d in thrombin and 315 in antithrombin, as a function of time, in the GaMD simulations of antithrombin complexes with wild type (WT) thrombin.

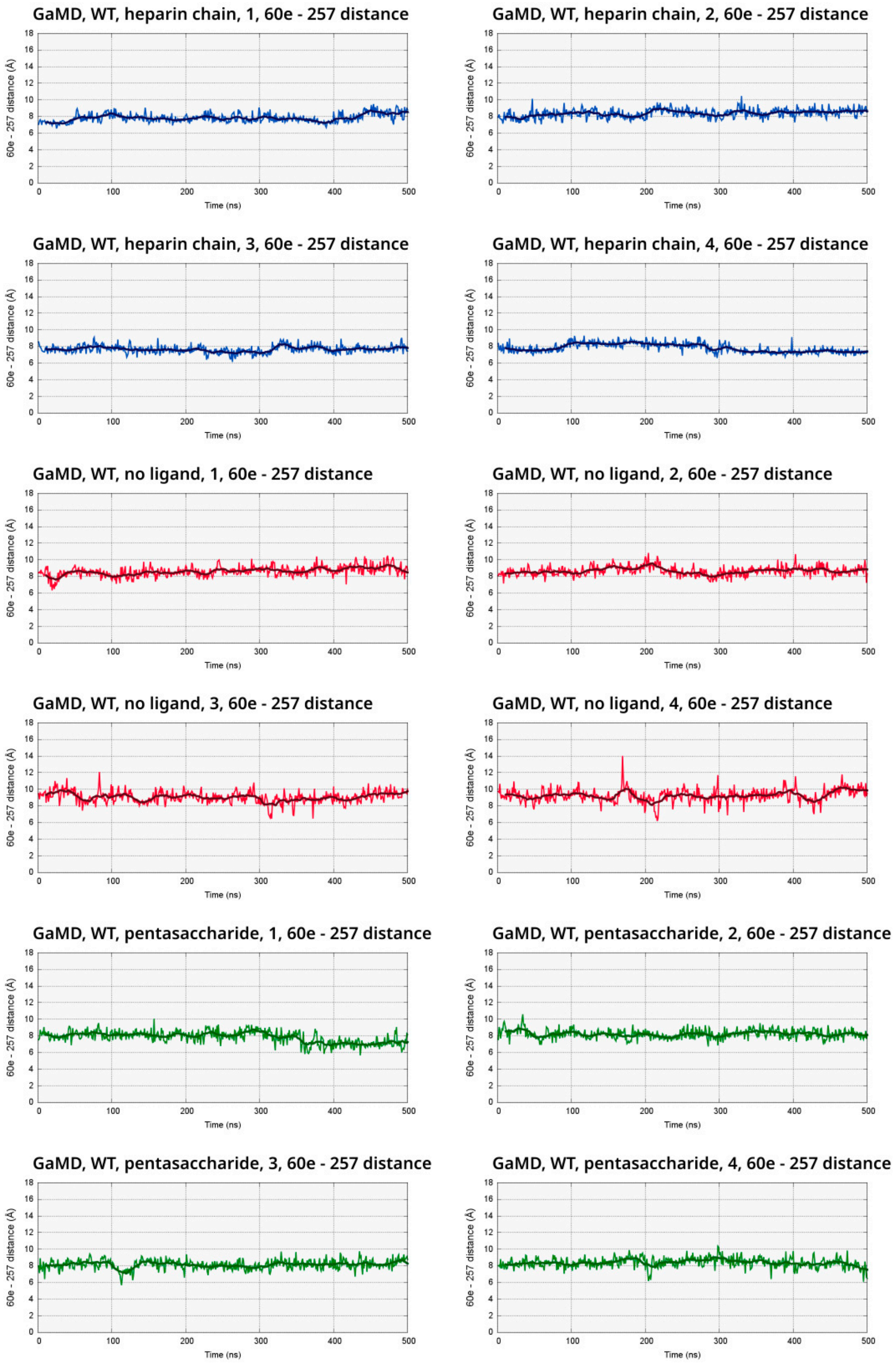

Figure S2. Distance between the alpha carbon atoms of amino acid 60e in thrombin and 257 in antithrombin, as a function of time, in the GaMD simulations of antithrombin complexes with wild type (WT) thrombin.

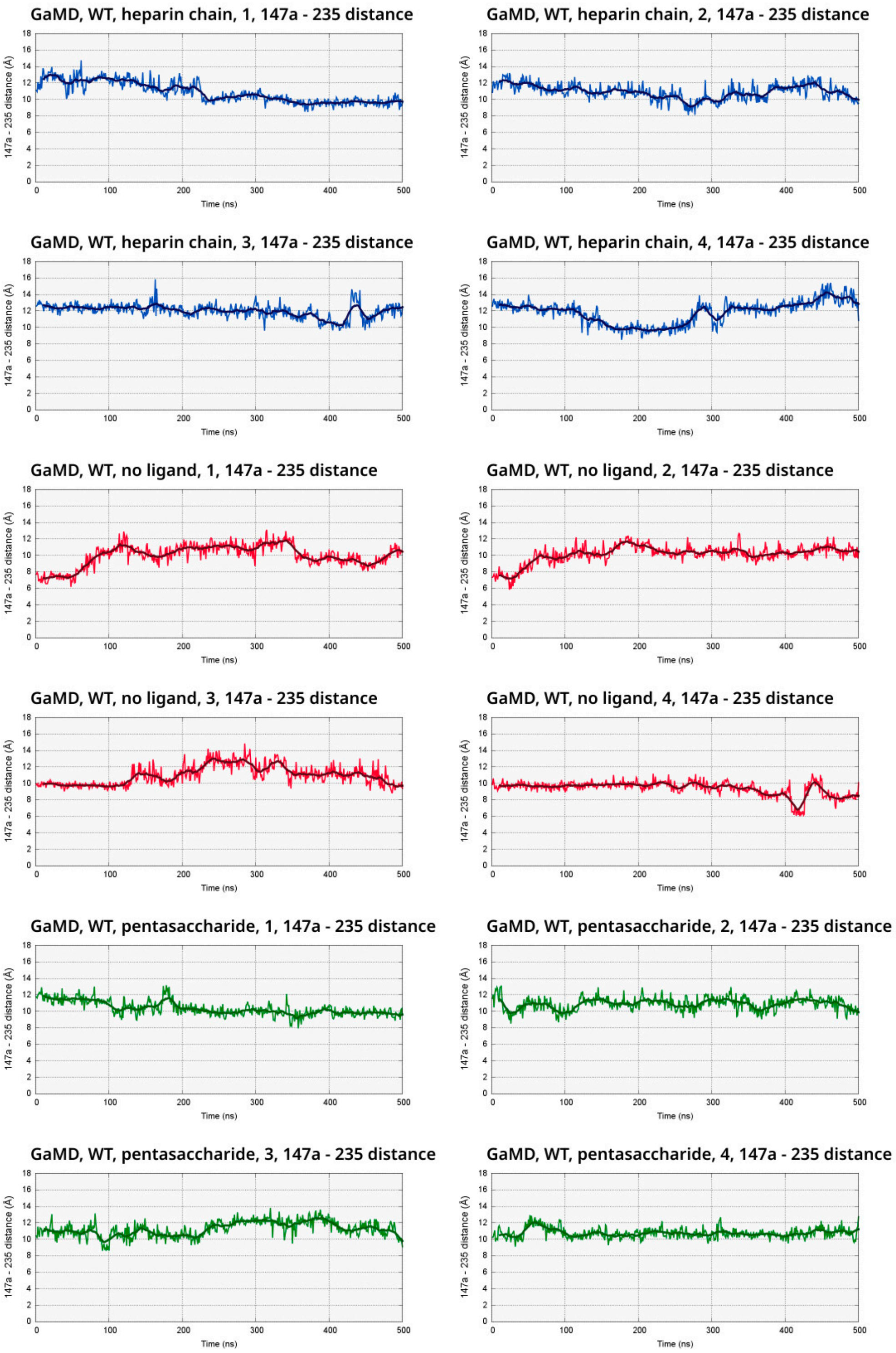

Figure S3. Distance between the alpha carbon atoms of amino acid 147a in thrombin and 235 in antithrombin, as a function of time, in the GaMD simulations of antithrombin complexes with wild type (WT) thrombin.

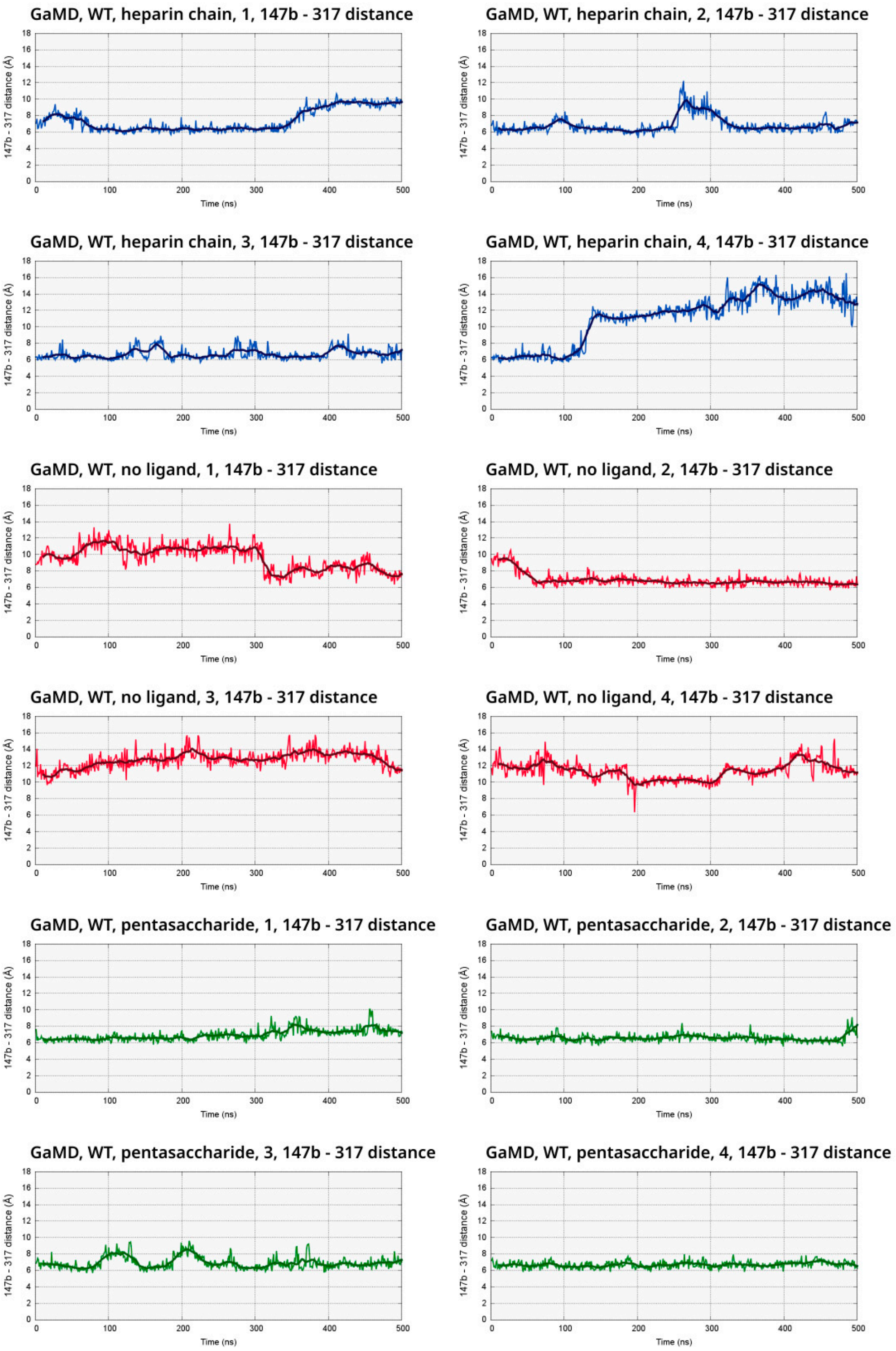

Figure S4. Distance between the alpha carbon atoms of amino acid 147b in thrombin and 317 in antithrombin, as a function of time, in the GaMD simulations of antithrombin complexes with wild type (WT) thrombin.

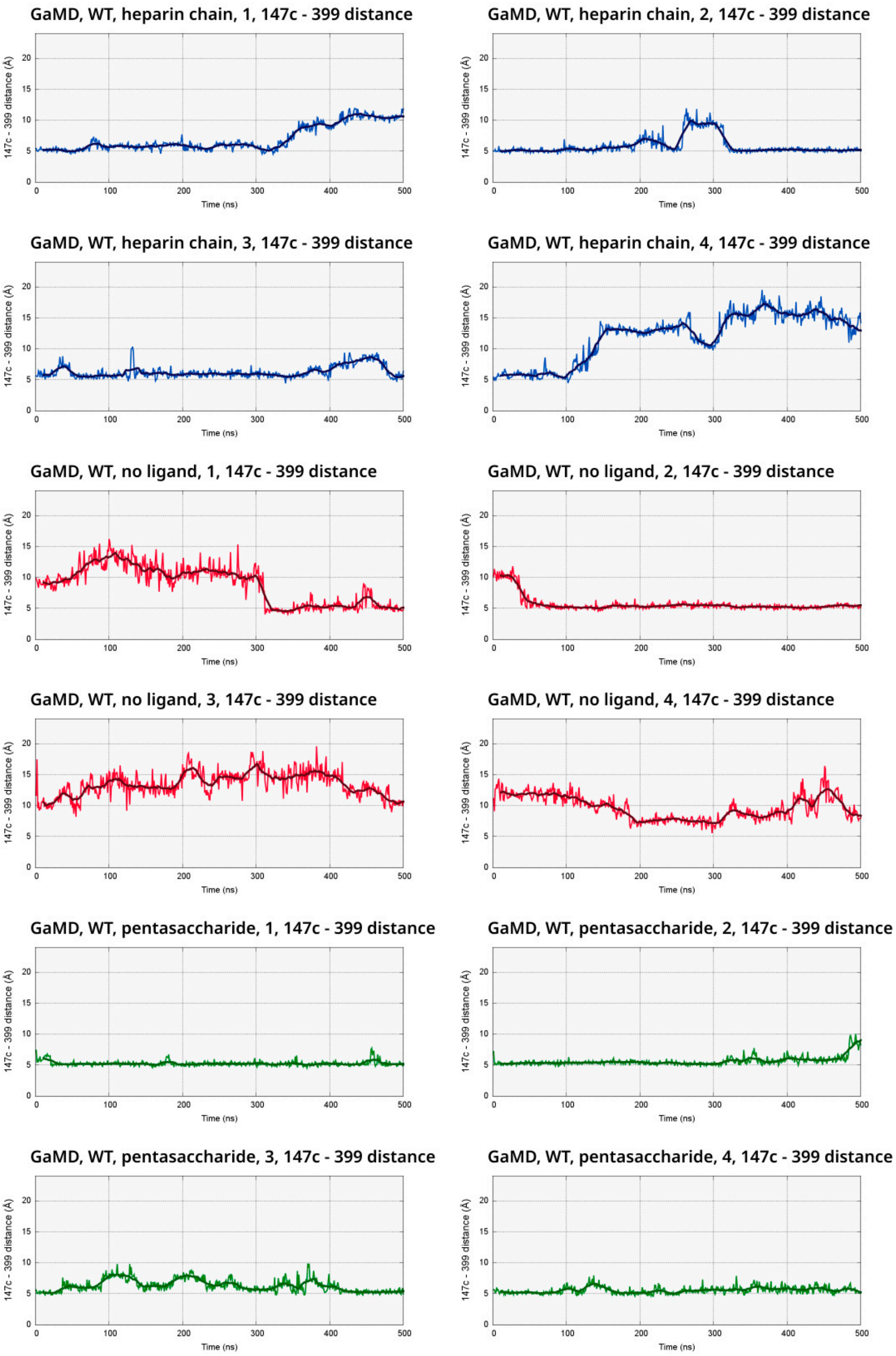

Figure S5. Distance between the alpha carbon atoms of amino acid 147c in thrombin and 399 in antithrombin, as a function of time, in the GaMD simulations of antithrombin complexes with wild type (WT) thrombin.

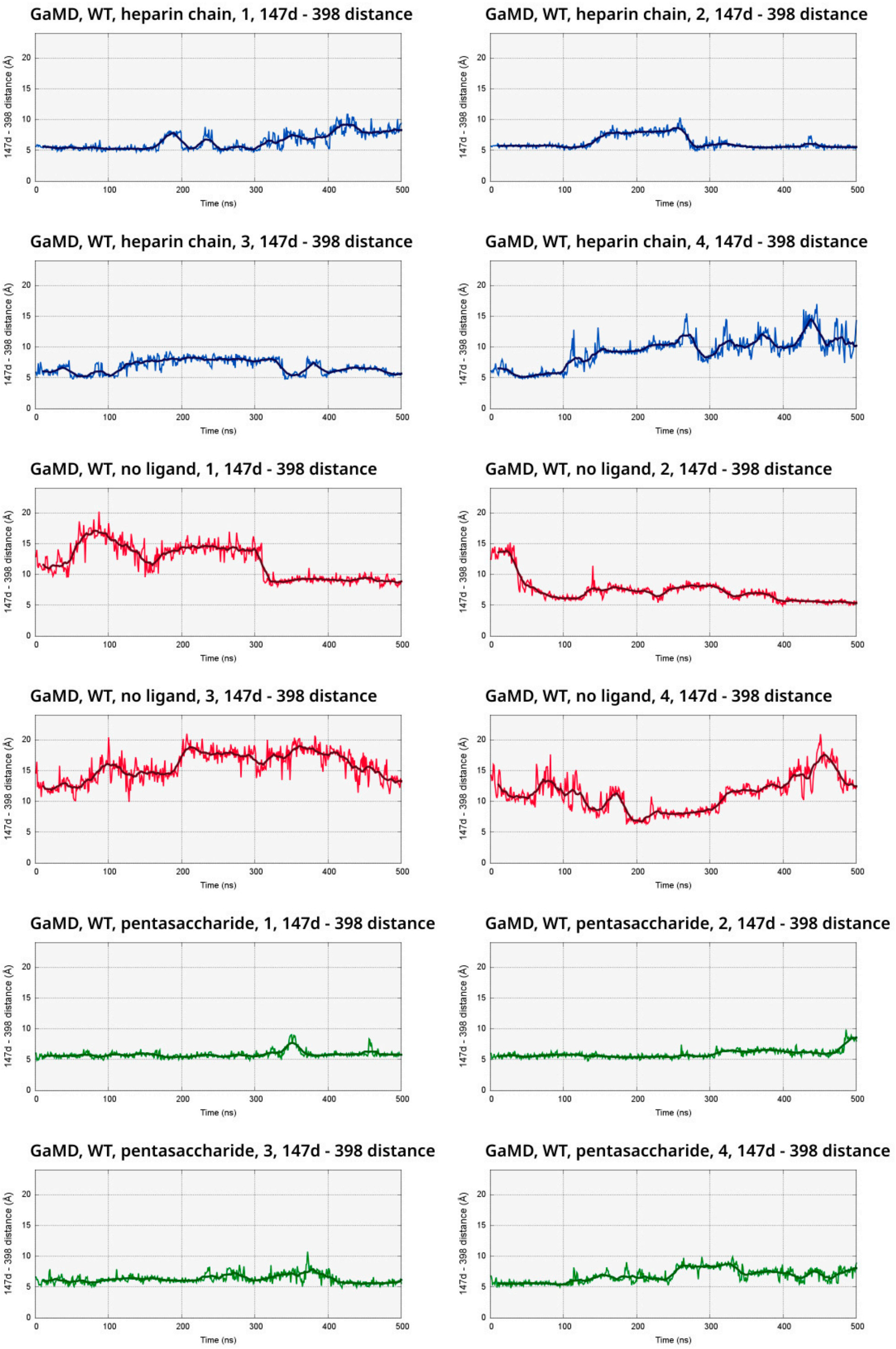

Figure S6. Distance between the alpha carbon atoms of amino acid 147d in thrombin and 398 in antithrombin, as a function of time, in the GaMD simulations of antithrombin complexes with wild type (WT) thrombin.

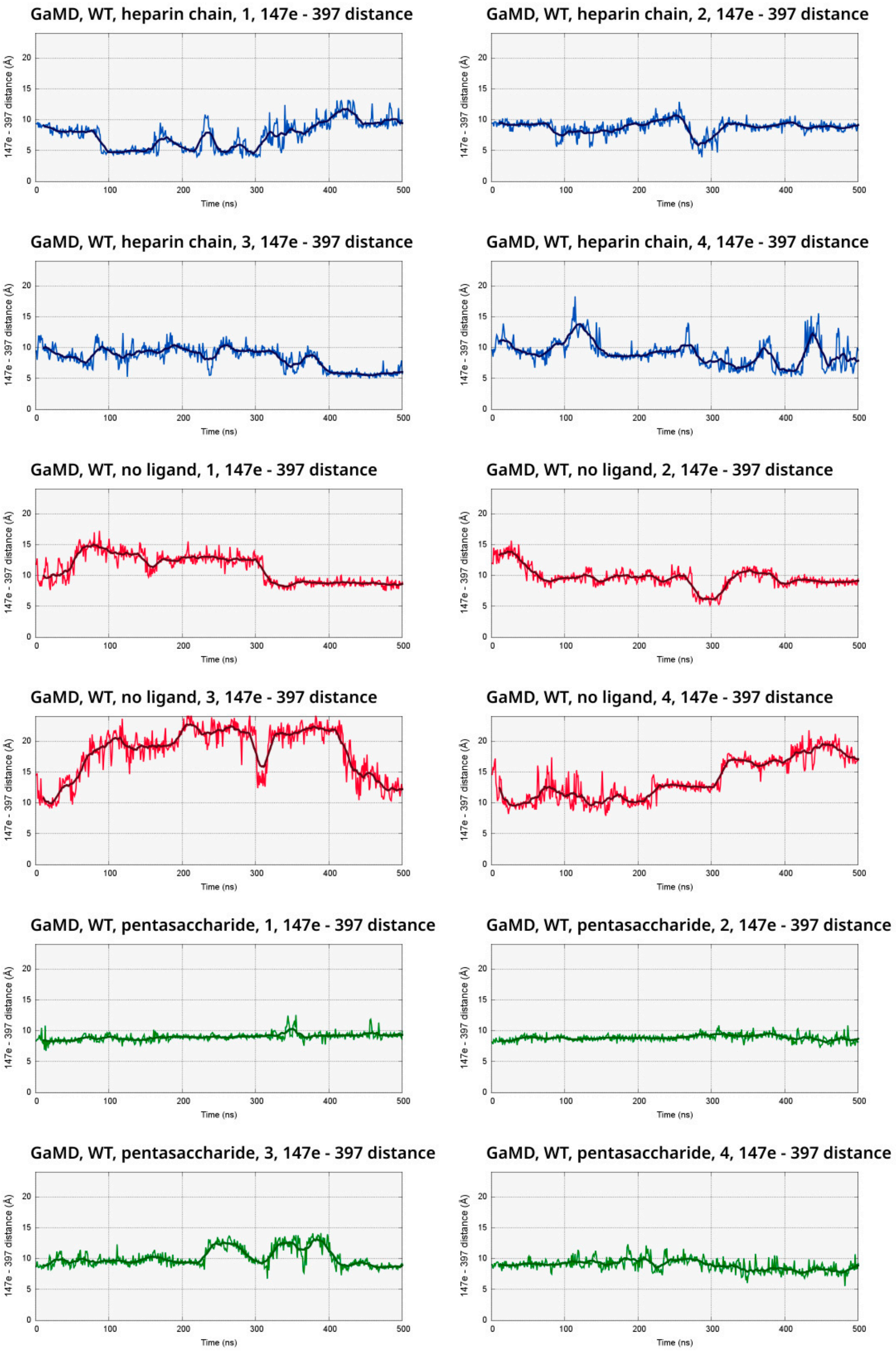

Figure S7. Distance between the alpha carbon atoms of amino acid 147e in thrombin and 397 in antithrombin, as a function of time, in the GaMD simulations of antithrombin complexes with wild type (WT) thrombin.

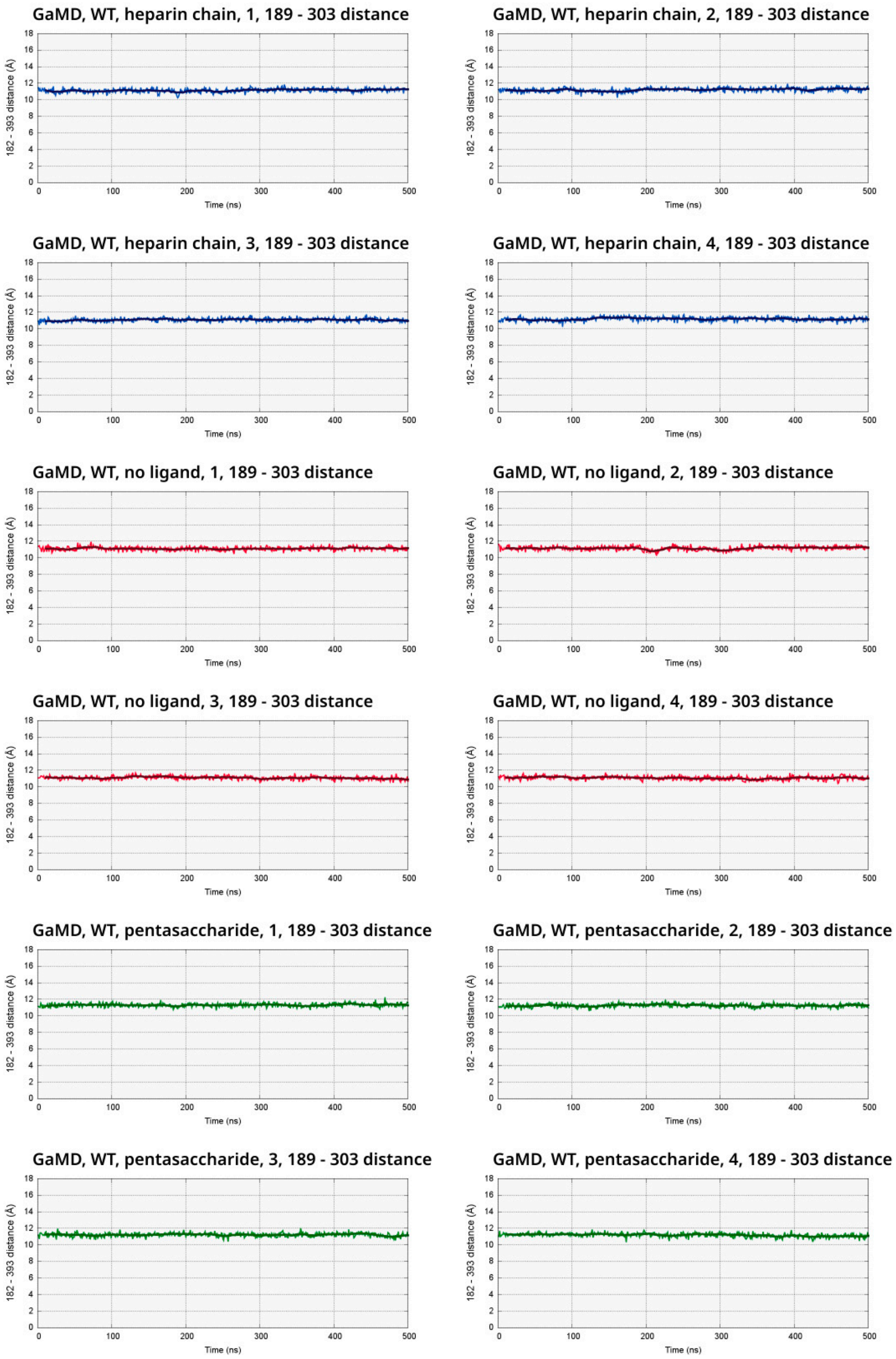

Figure S8. Distance between the alpha carbon atoms of amino acid 189 in thrombin and 393 in antithrombin, as a function of time, in the GaMD simulations of antithrombin complexes with wild type (WT) thrombin.

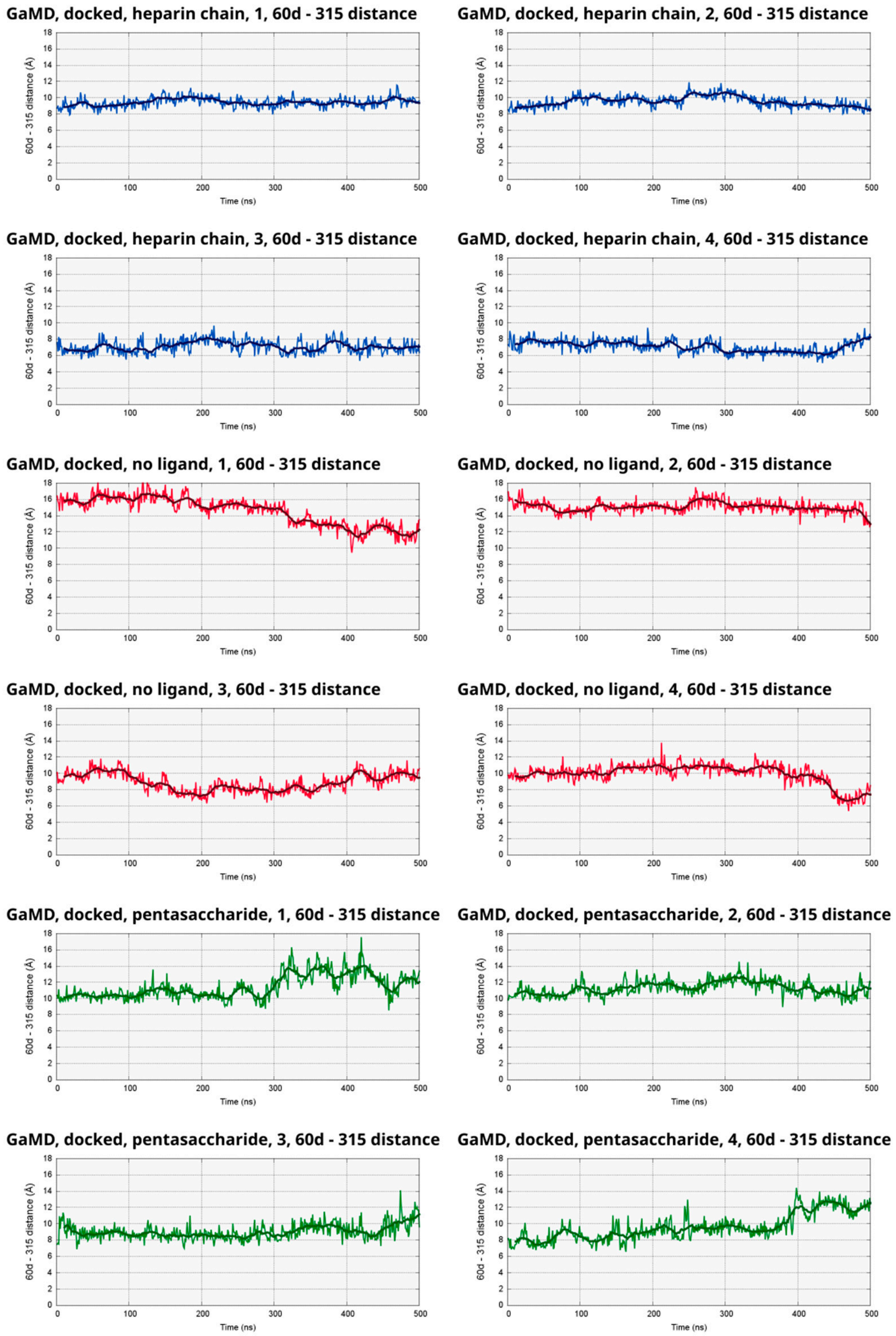

Figure S9. Distance between the alpha carbon atoms of amino acid 60d in thrombin and 315 in antithrombin, as a function of time, in the GaMD simulations of the antithrombin-thrombin complexes built using docking.

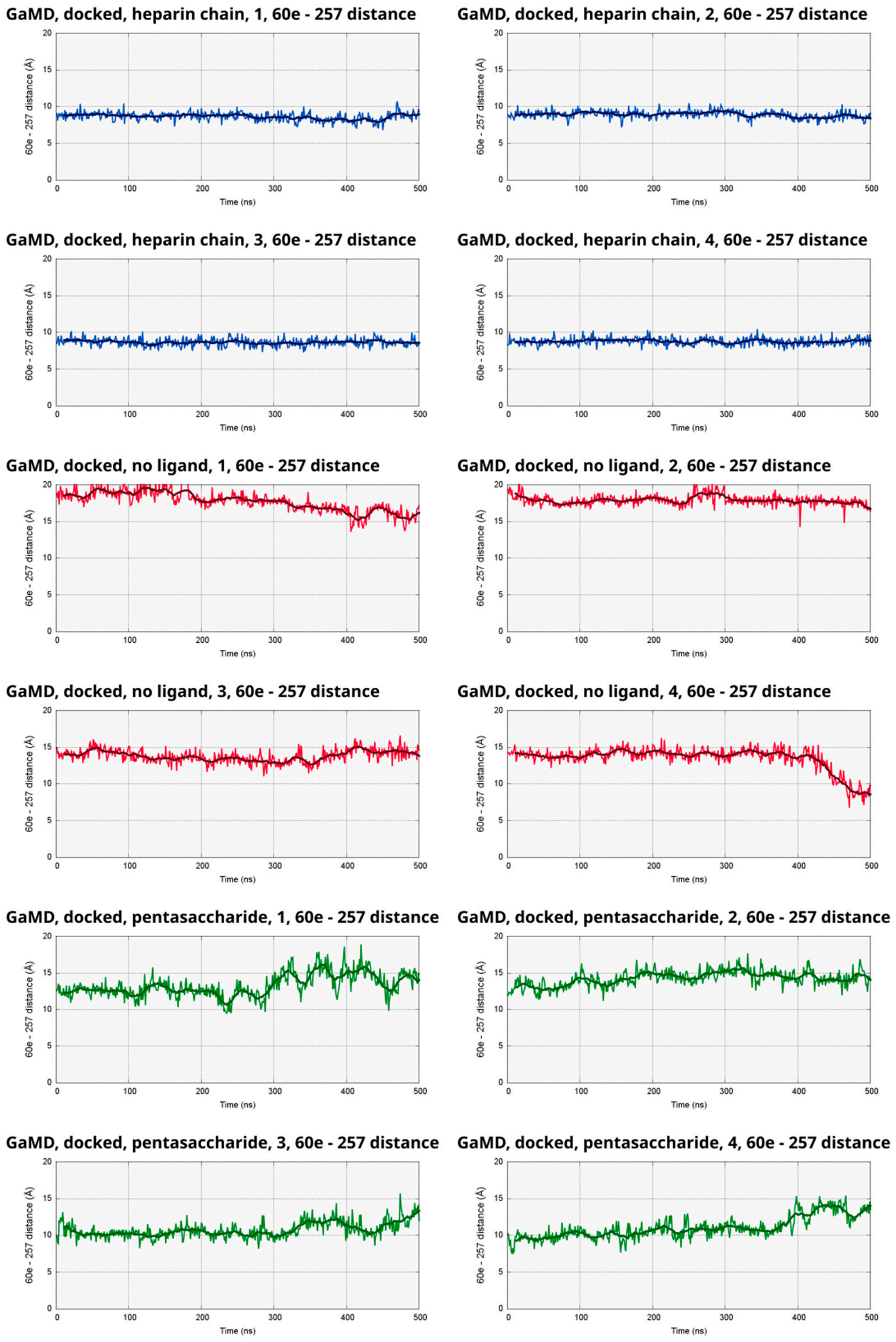

Figure S10. Distance between the alpha carbon atoms of amino acid 60e in thrombin and 257 in antithrombin, as a function of time, in the GaMD simulations of the antithrombin-thrombin complexes built using docking.

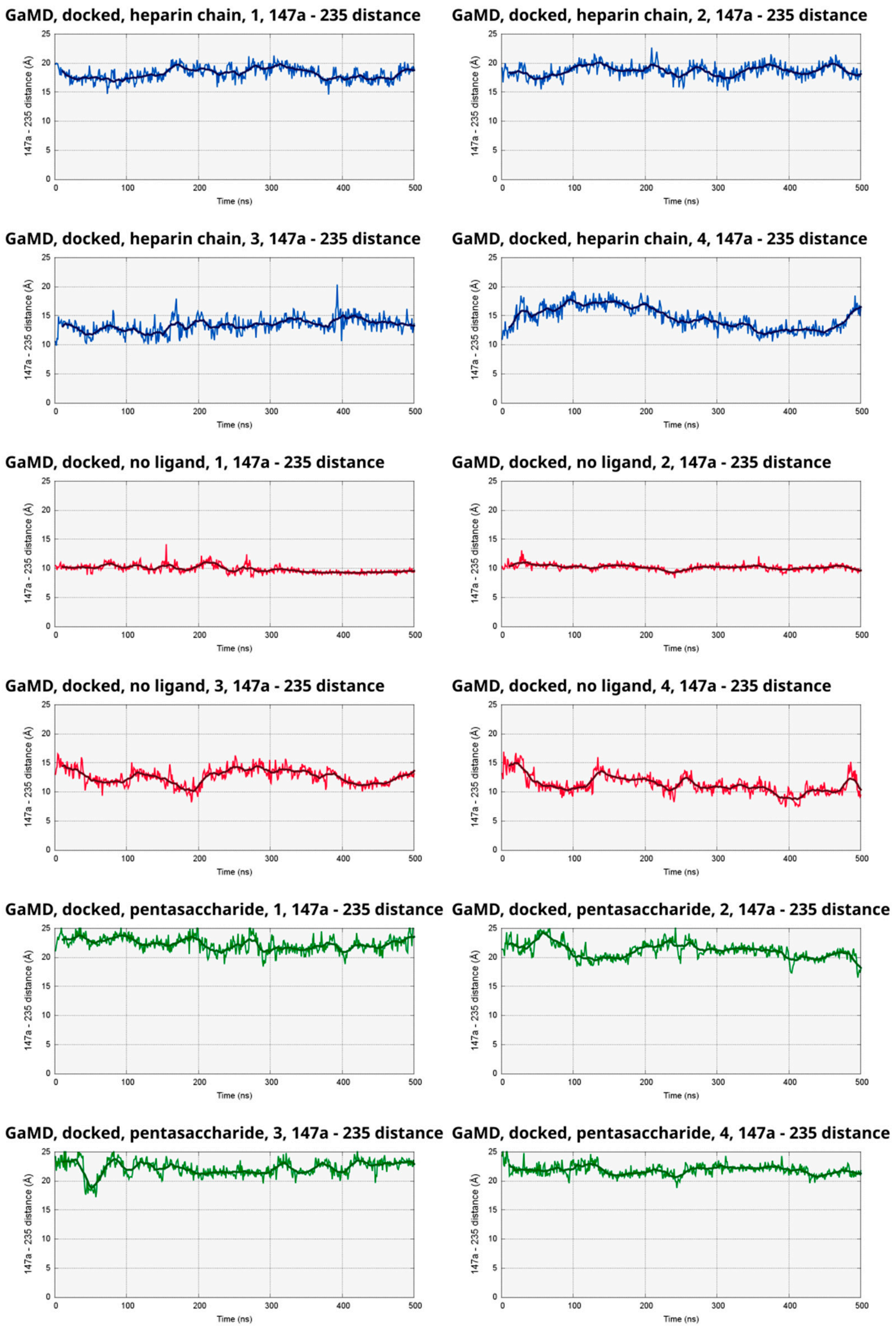

Figure S11. Distance between the alpha carbon atoms of amino acid 147a in thrombin and 235 in antithrombin, as a function of time, in the GaMD simulations of the antithrombin-thrombin complexes built using docking.

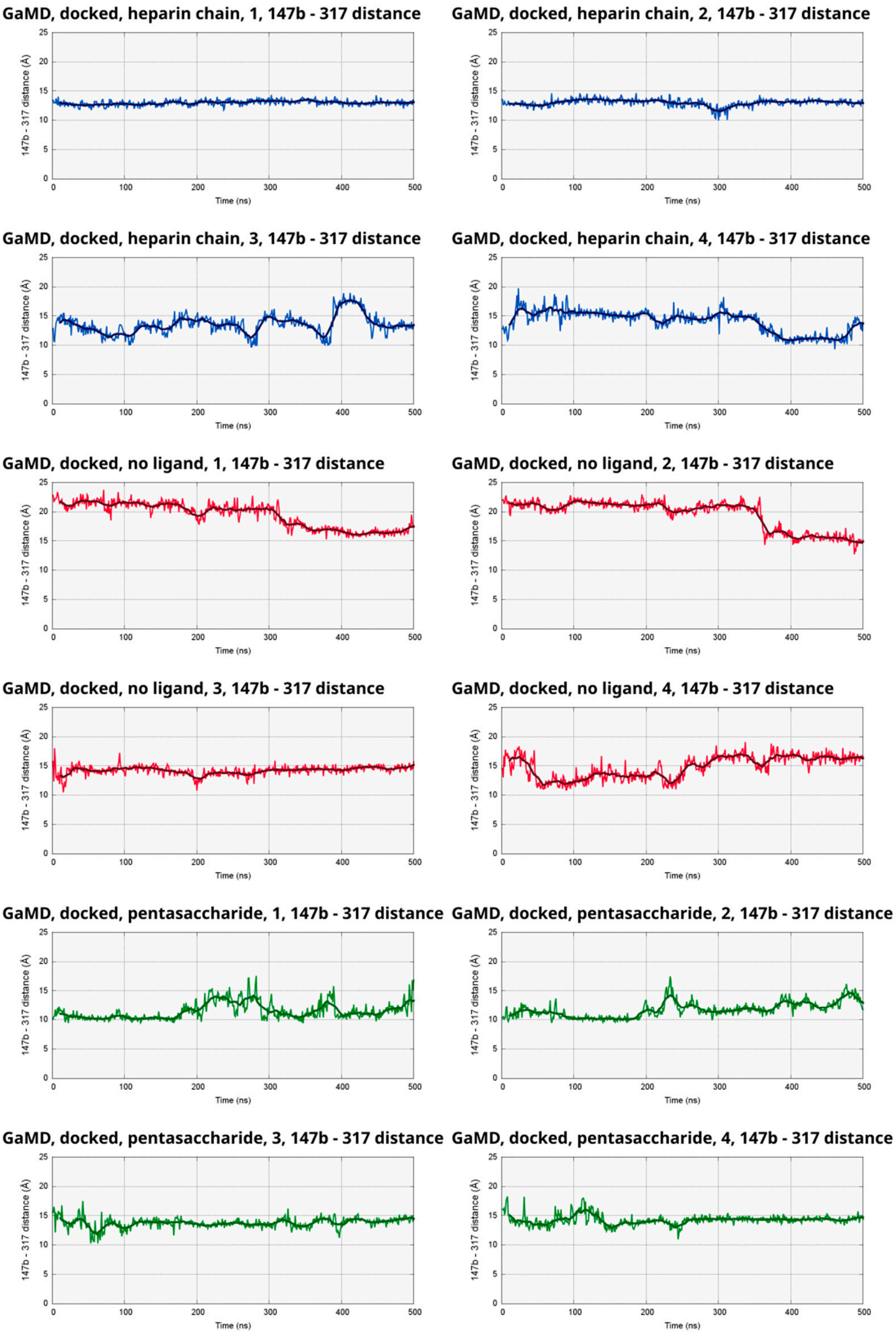

Figure S12. Distance between the alpha carbon atoms of amino acid 147b in thrombin and 317 in antithrombin, as a function of time, in the GaMD simulations of the antithrombin-thrombin complexes built using docking.

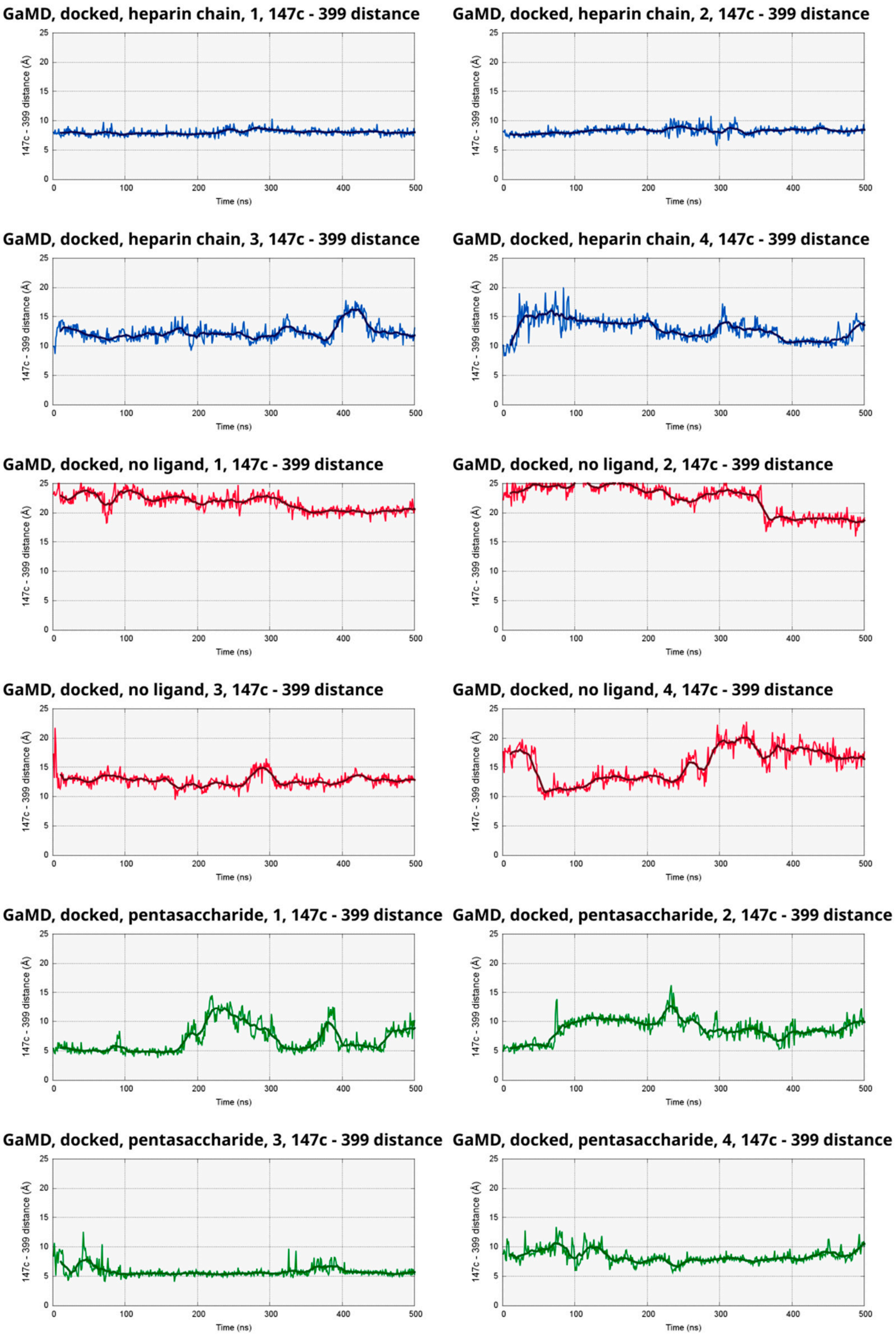

Figure S13. Distance between the alpha carbon atoms of amino acid 147c in thrombin and 399 in antithrombin, as a function of time, in the GaMD simulations of the antithrombin-thrombin complexes built using docking.

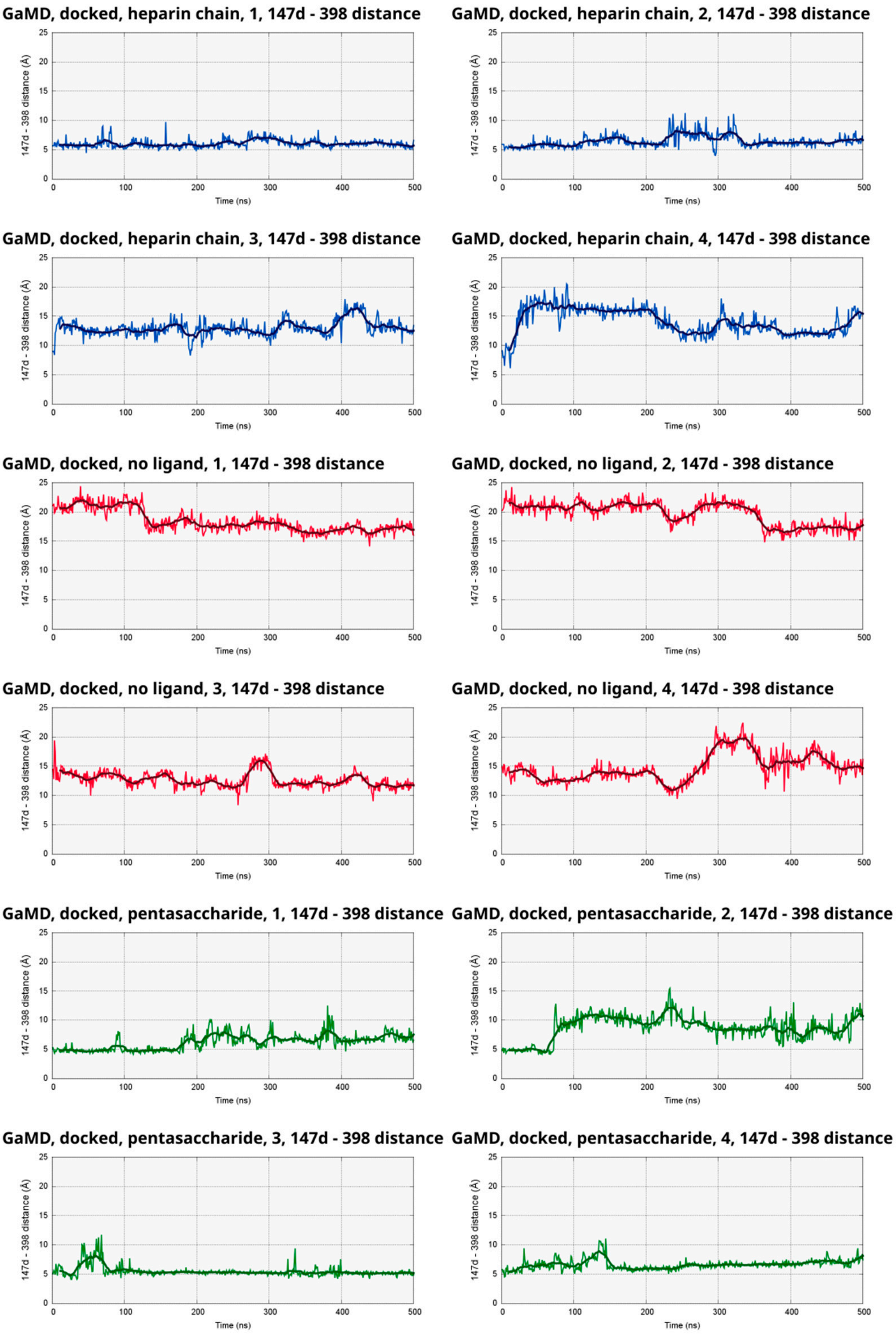

Figure S14. Distance between the alpha carbon atoms of amino acid 147d in thrombin and 398 in antithrombin, as a function of time, in the GaMD simulations of the antithrombin-thrombin complexes built using docking.

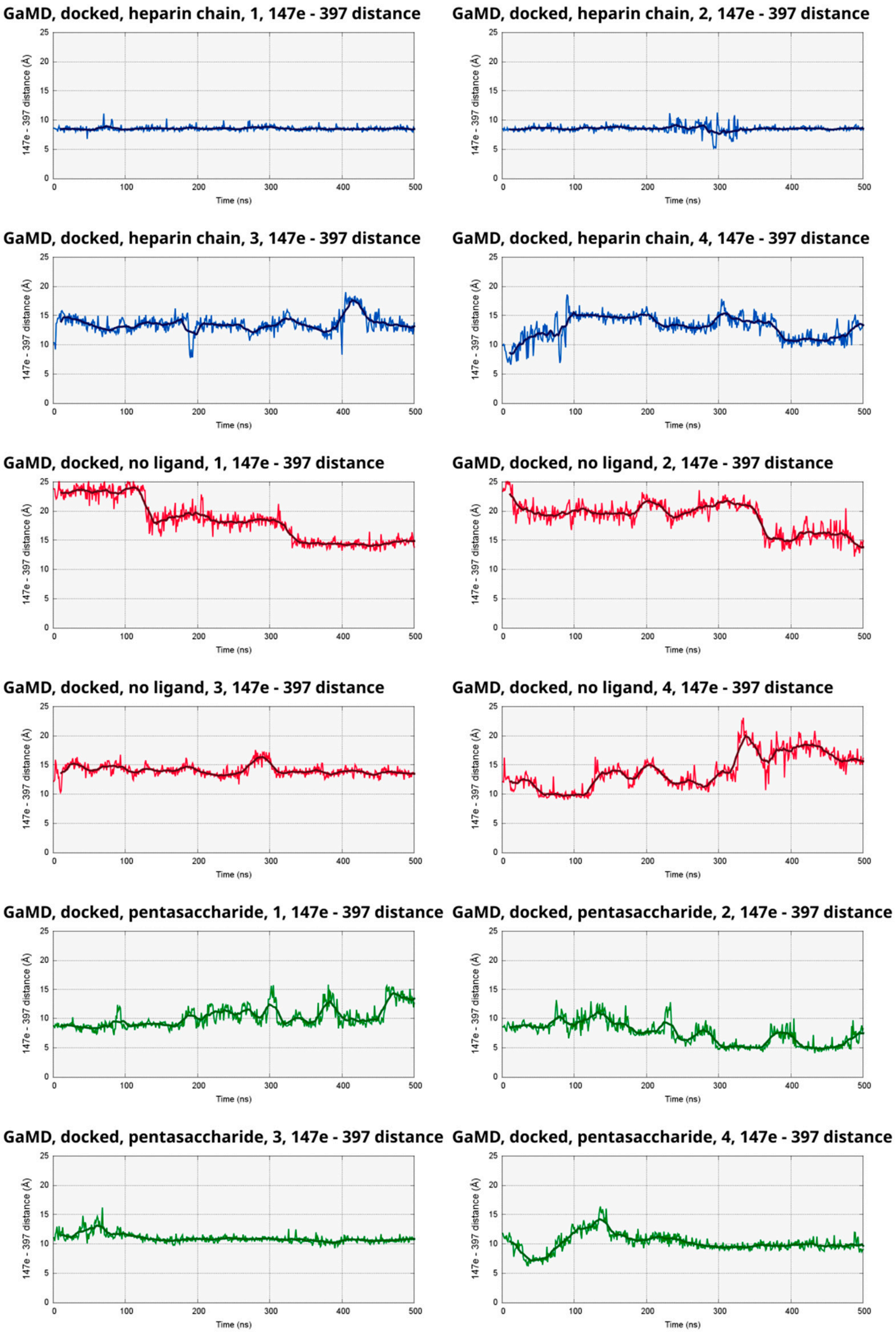

Figure S15. Distance between the alpha carbon atoms of amino acid 147e in thrombin and 397 in antithrombin, as a function of time, in the GaMD simulations of the antithrombin-thrombin complexes built using docking.

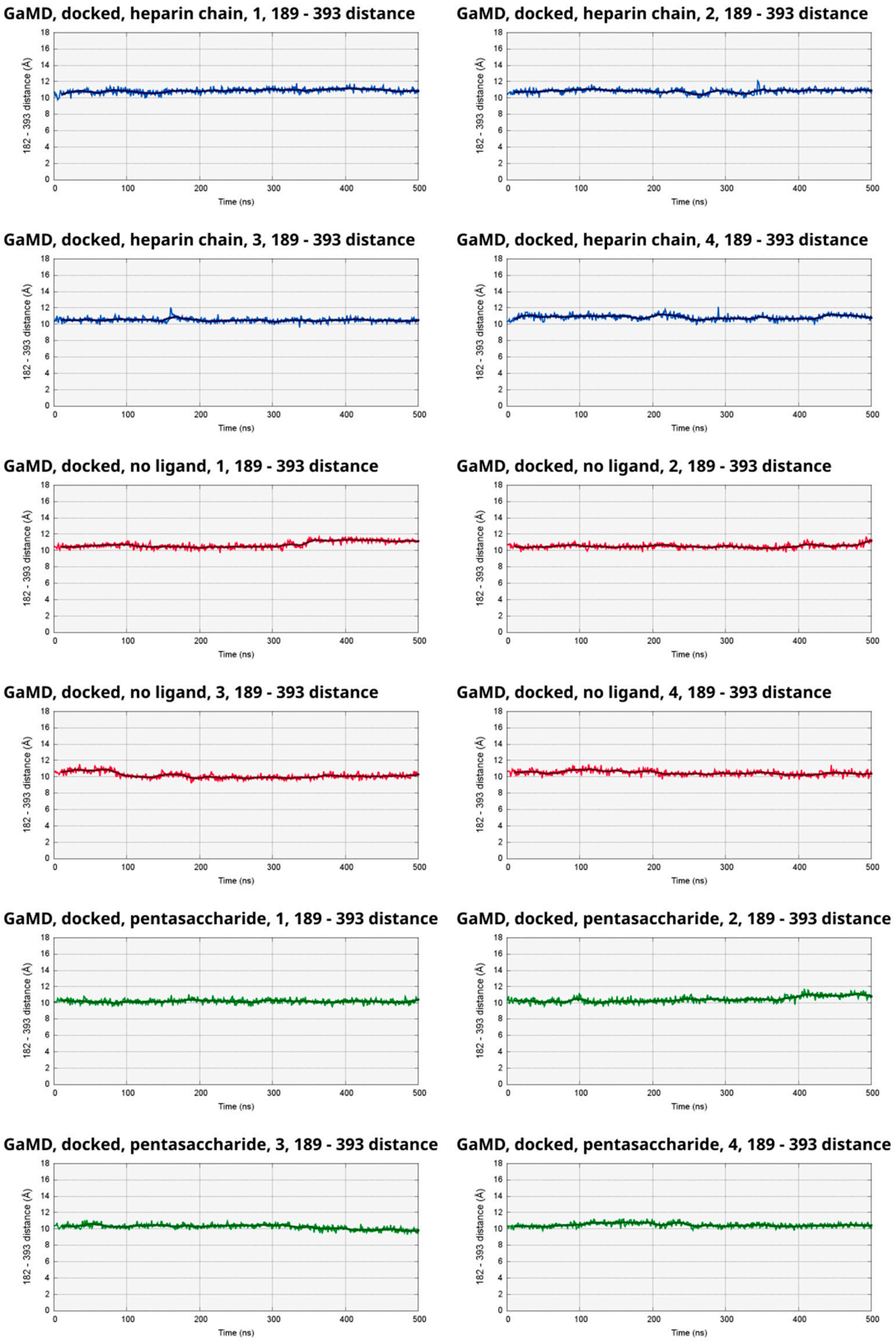

Figure S16. Distance between the alpha carbon atoms of amino acid 189 in thrombin and 393 in antithrombin, as a function of time, in the GaMD simulations of the antithrombin-thrombin complexes built using docking.

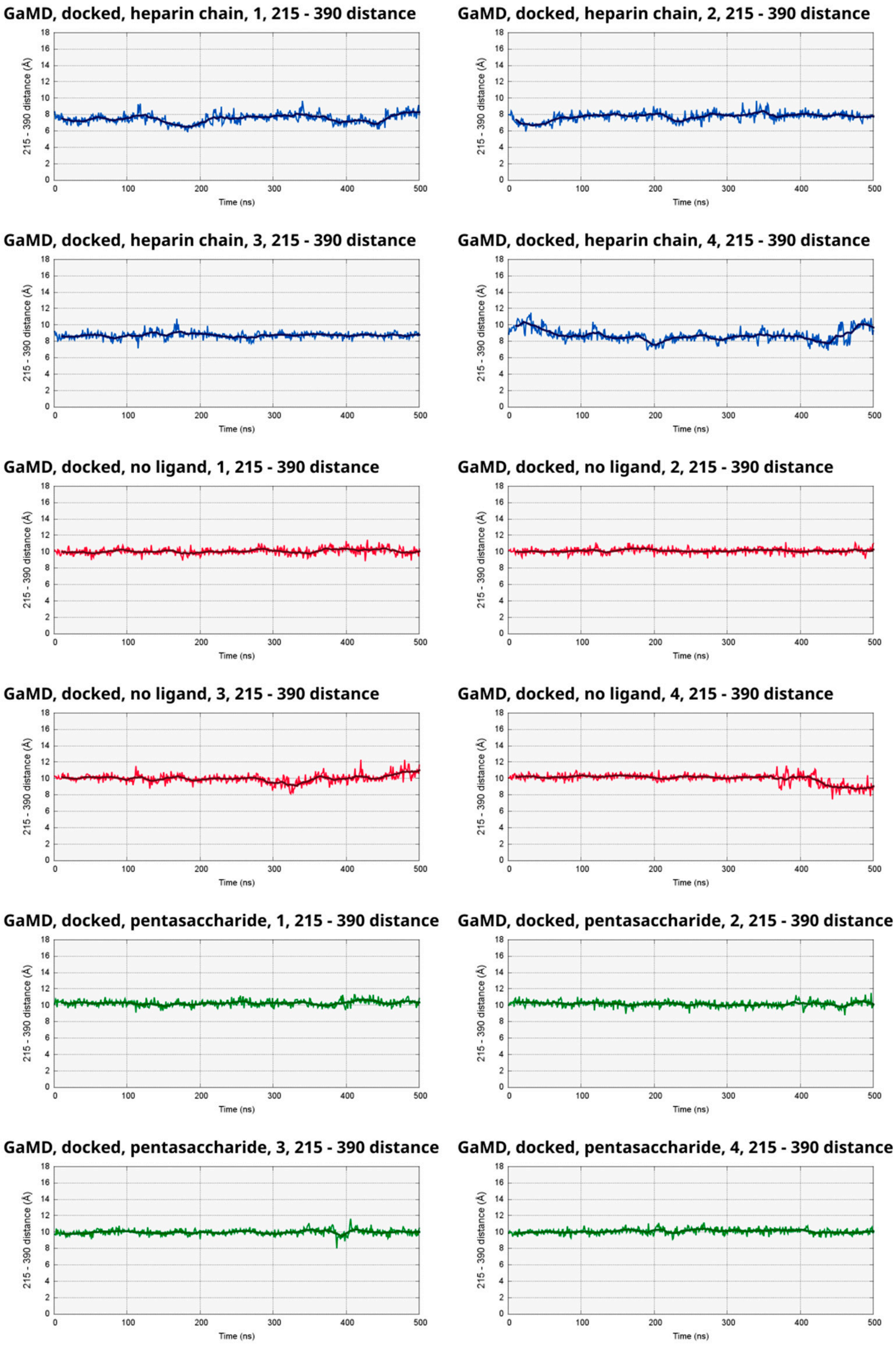

Figure S17. Distance between the alpha carbon atoms of amino acid 215 in thrombin and 390 in antithrombin, as a function of time, in the GaMD simulations of the antithrombin-thrombin complexes built using docking.

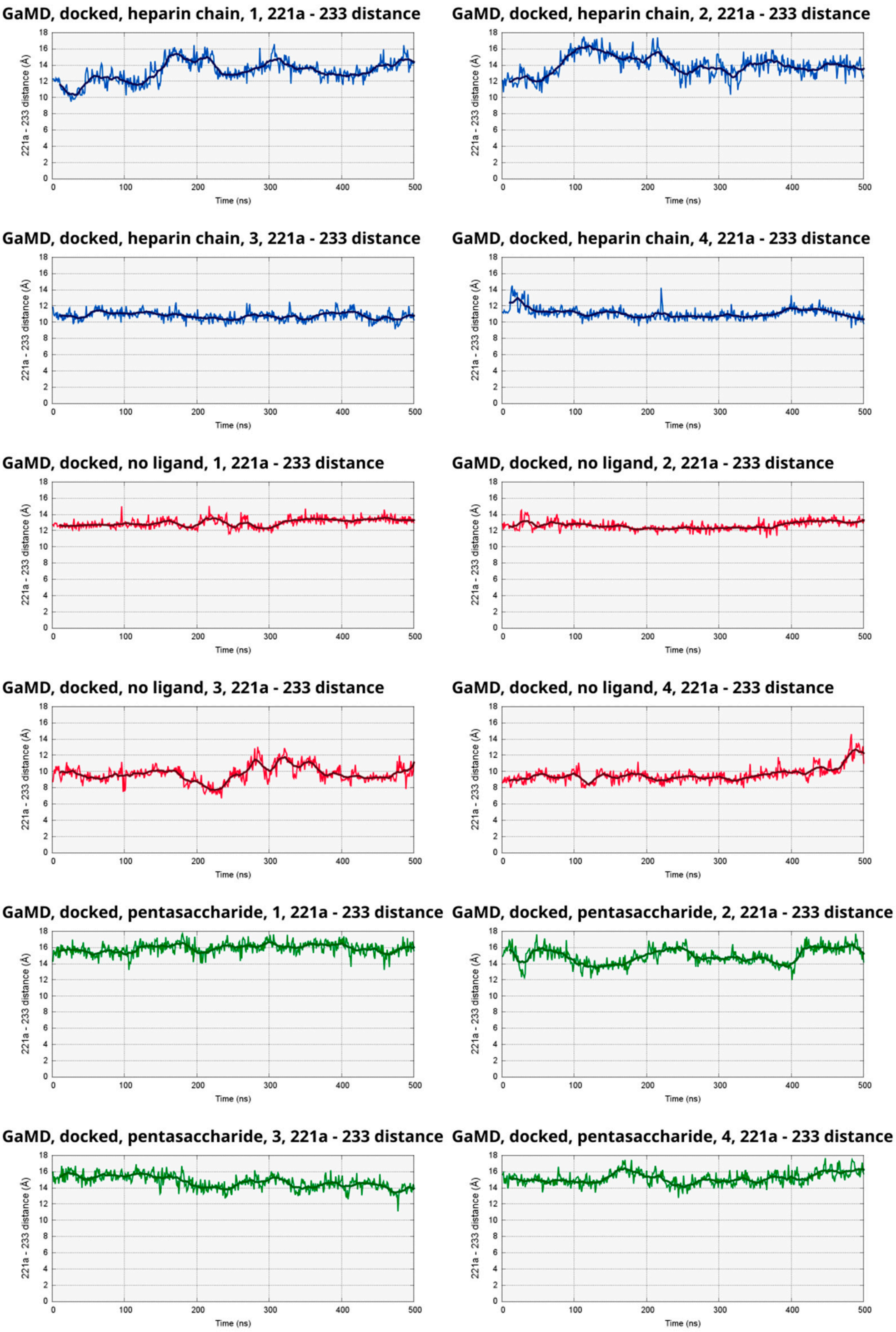

Figure S18. Distance between the alpha carbon atoms of amino acid 221a in thrombin and 233 in antithrombin, as a function of time, in the GaMD simulations of the antithrombin-thrombin complexes built using docking.

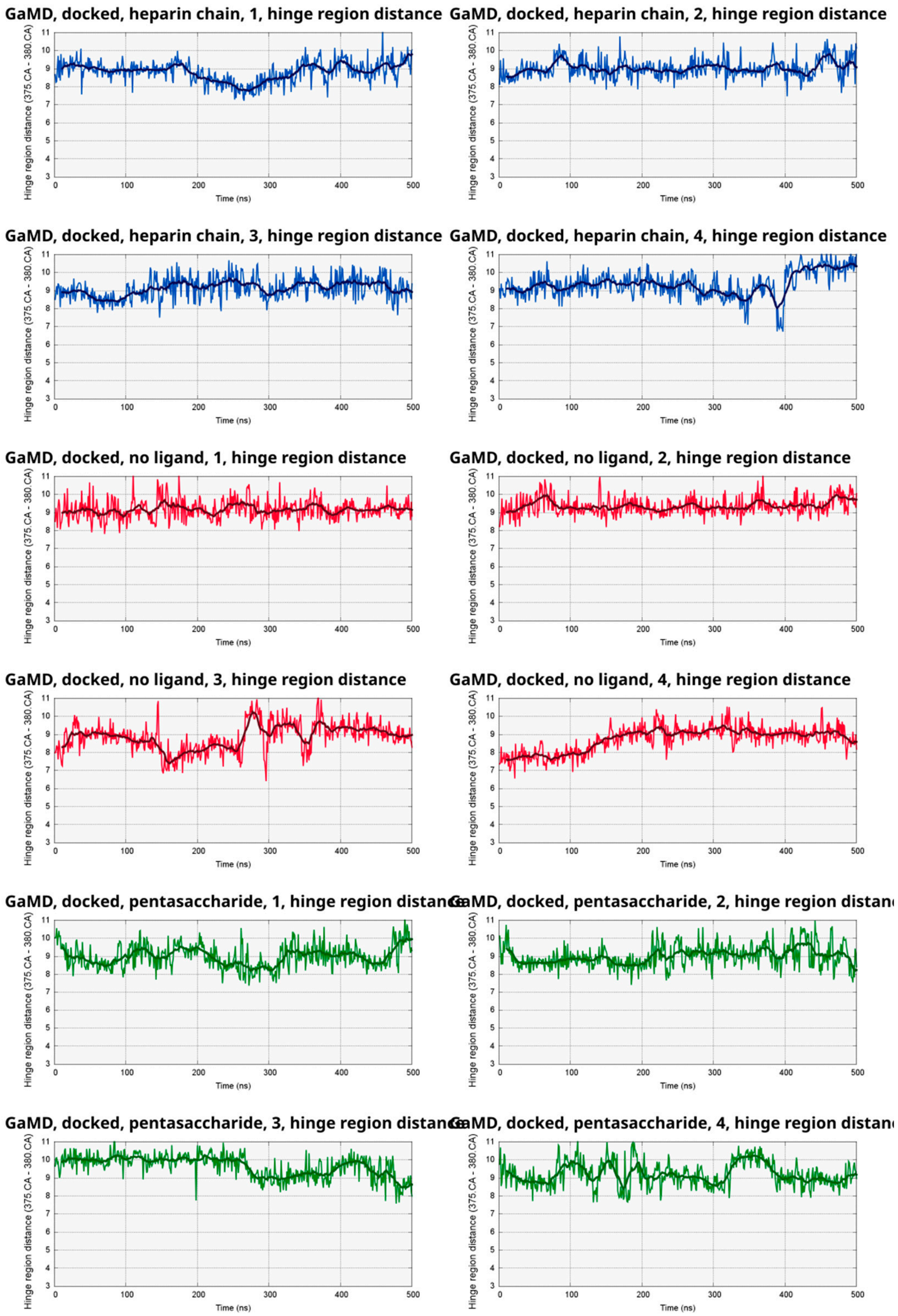

Figure S19. Distance between the alpha carbon atoms of amino acids 375 and 380 in antithrombin, corresponding to the position of the hinge region, as a function of time, in the GaMD simulations of the antithrombin-thrombin complexes built using docking.

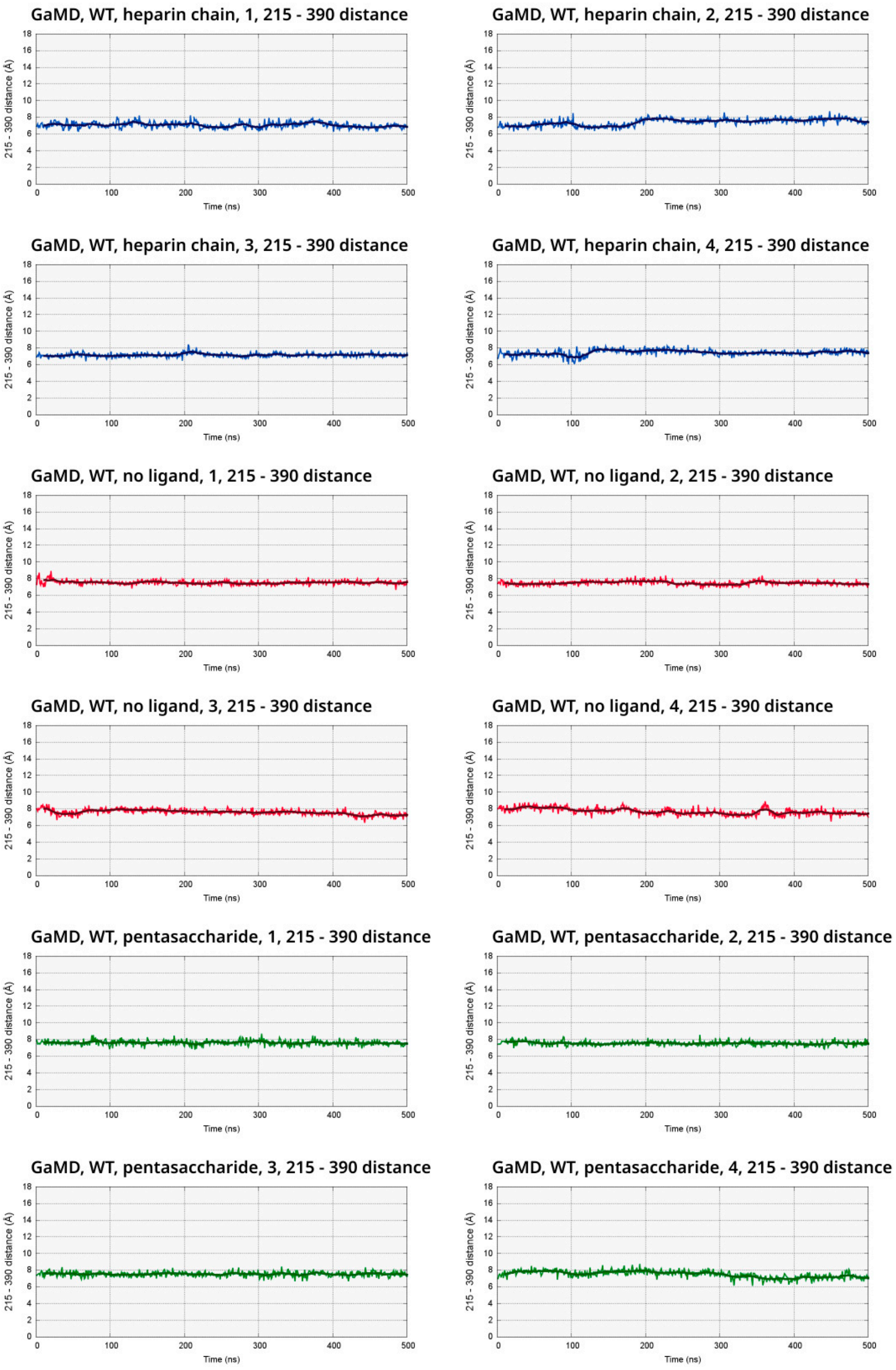

Figure S20. Distance between the alpha carbon atoms of amino acid 215 in thrombin and 390 in antithrombin, as a function of time, in the GaMD simulations of antithrombin complexes with wild type (WT) thrombin.

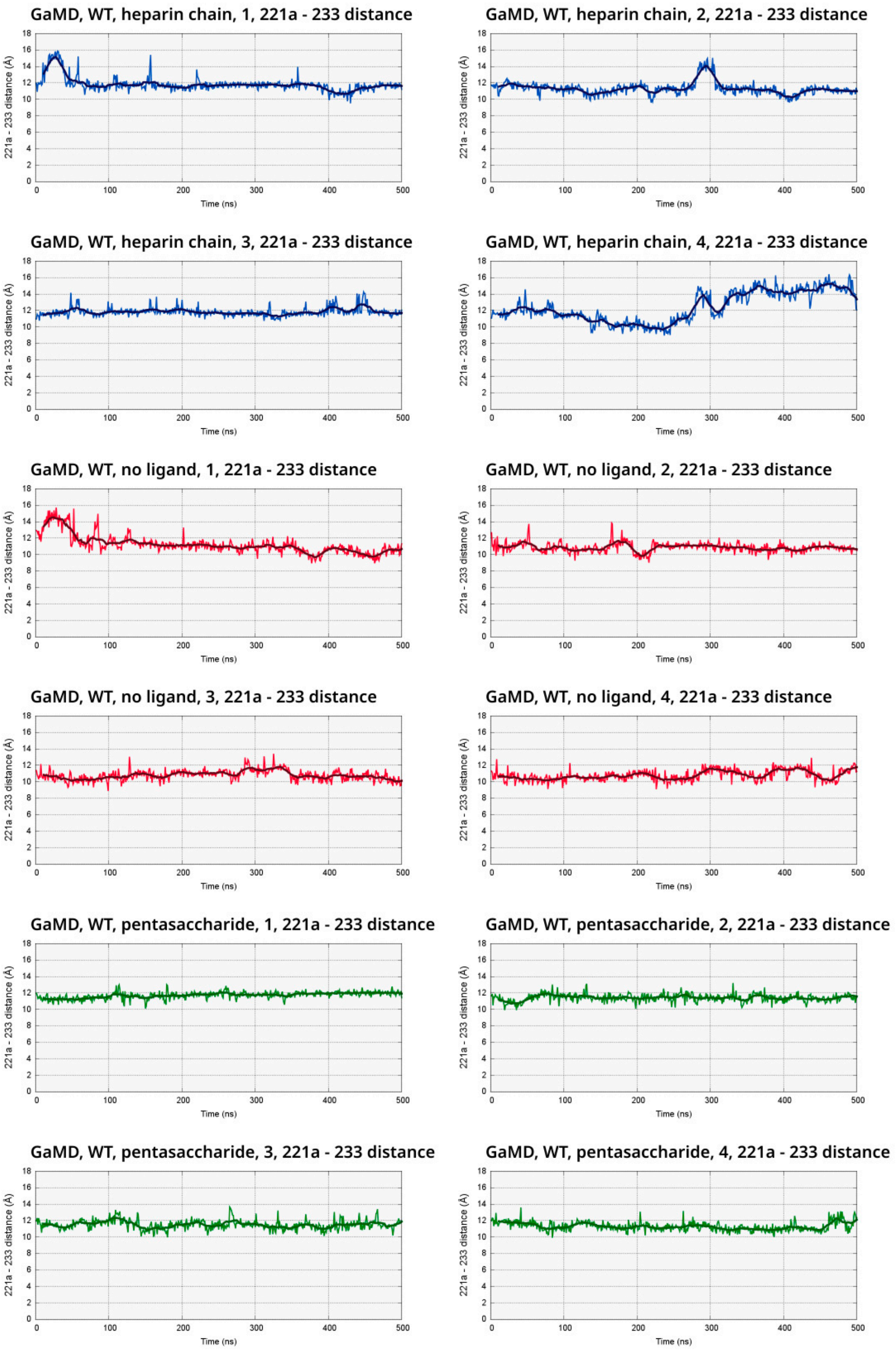

Figure S21. Distance between the alpha carbon atoms of amino acid 221a in thrombin and 233 in antithrombin, as a function of time, in the GaMD simulations of antithrombin complexes with wild type (WT) thrombin.

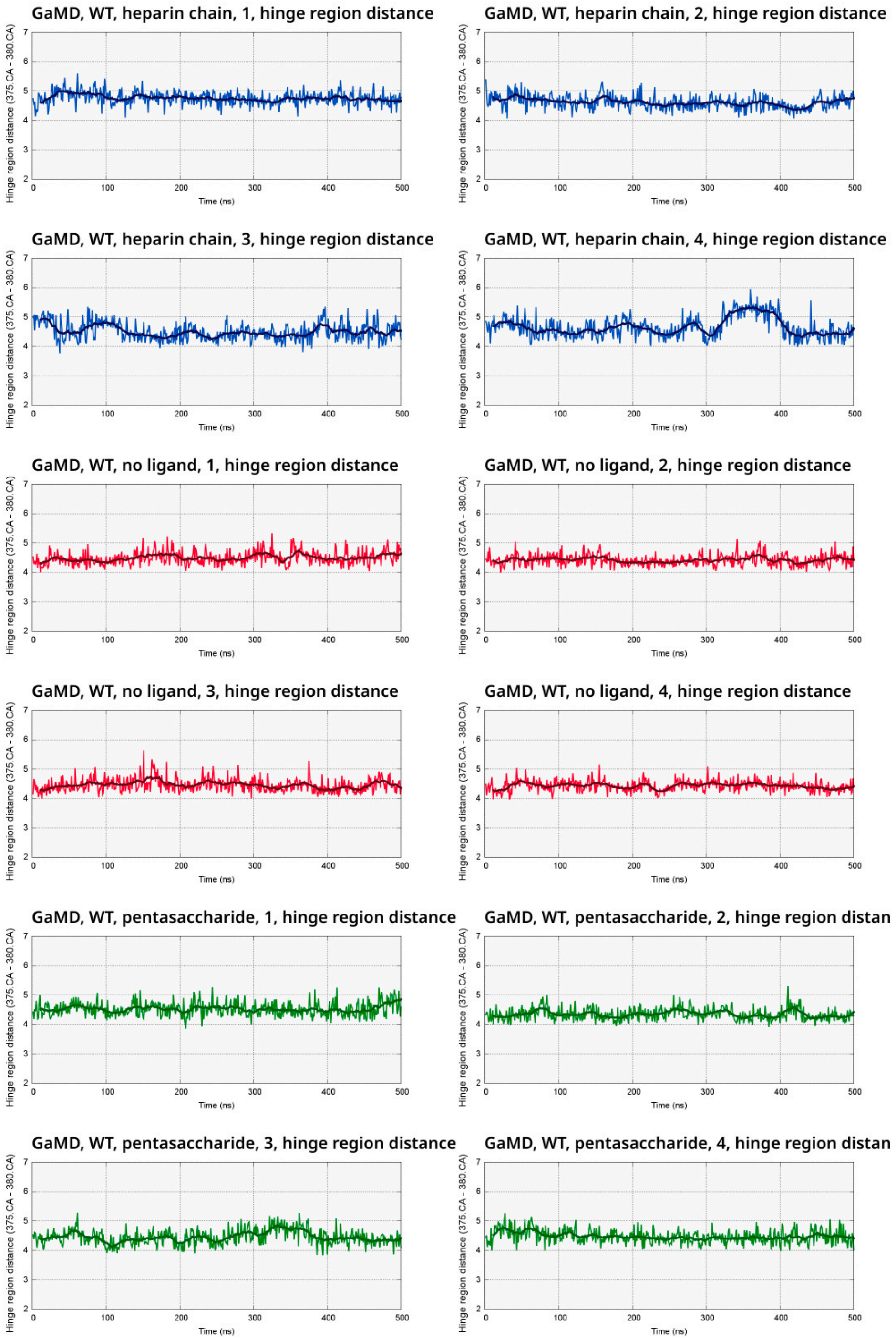

Figure S22. Distance between the alpha carbon atoms of amino acids 375 and 380 in antithrombin, corresponding to the position of the hinge region, as a function of time, in the GaMD simulations of antithrombin complexes with wild type (WT) thrombin.

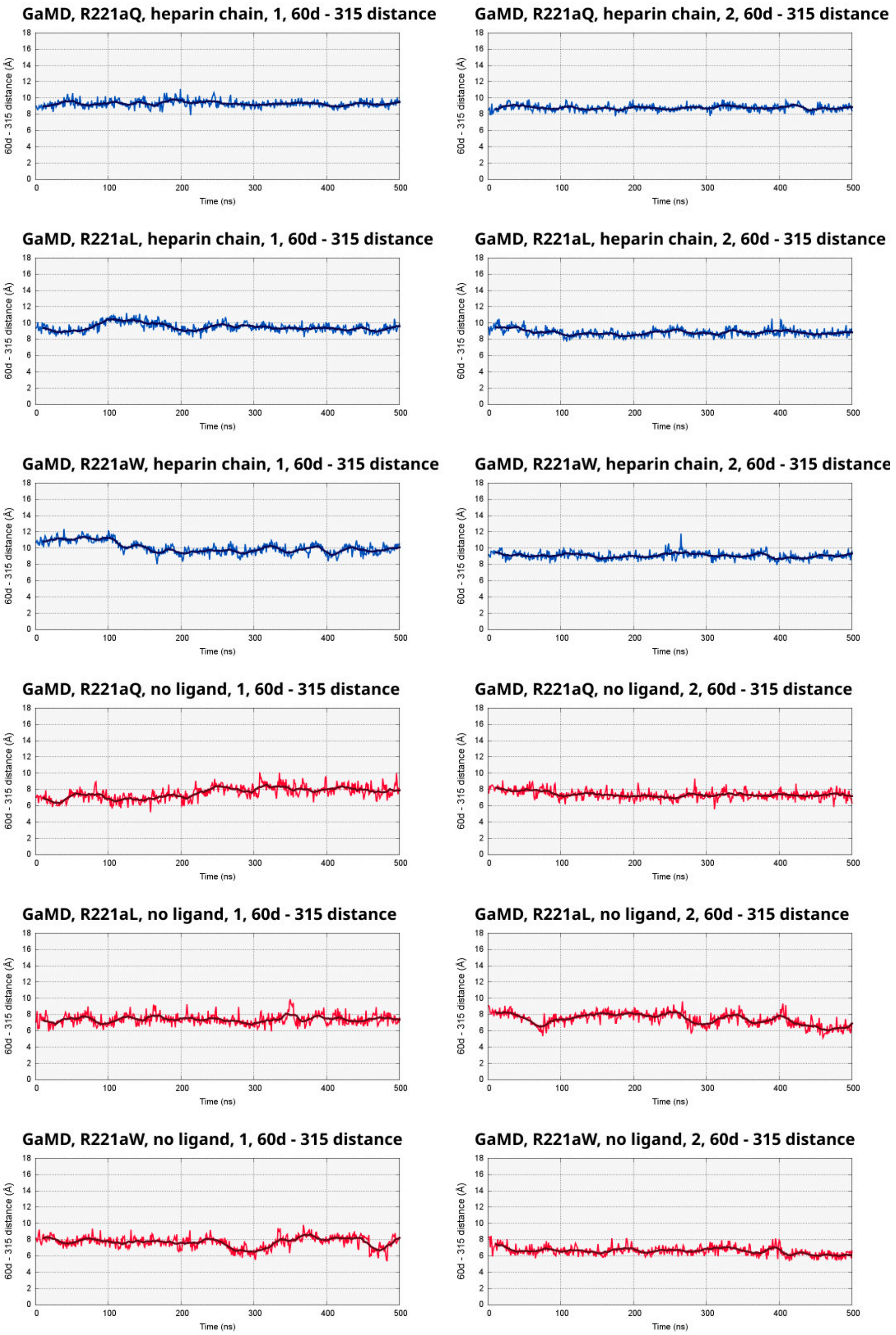

Figure S23. Distance between the alpha carbon atoms of amino acid 60d in thrombin and 315 in antithrombin, as a function of time, in the GaMD simulations of antithrombin complexes with thrombin mutants.

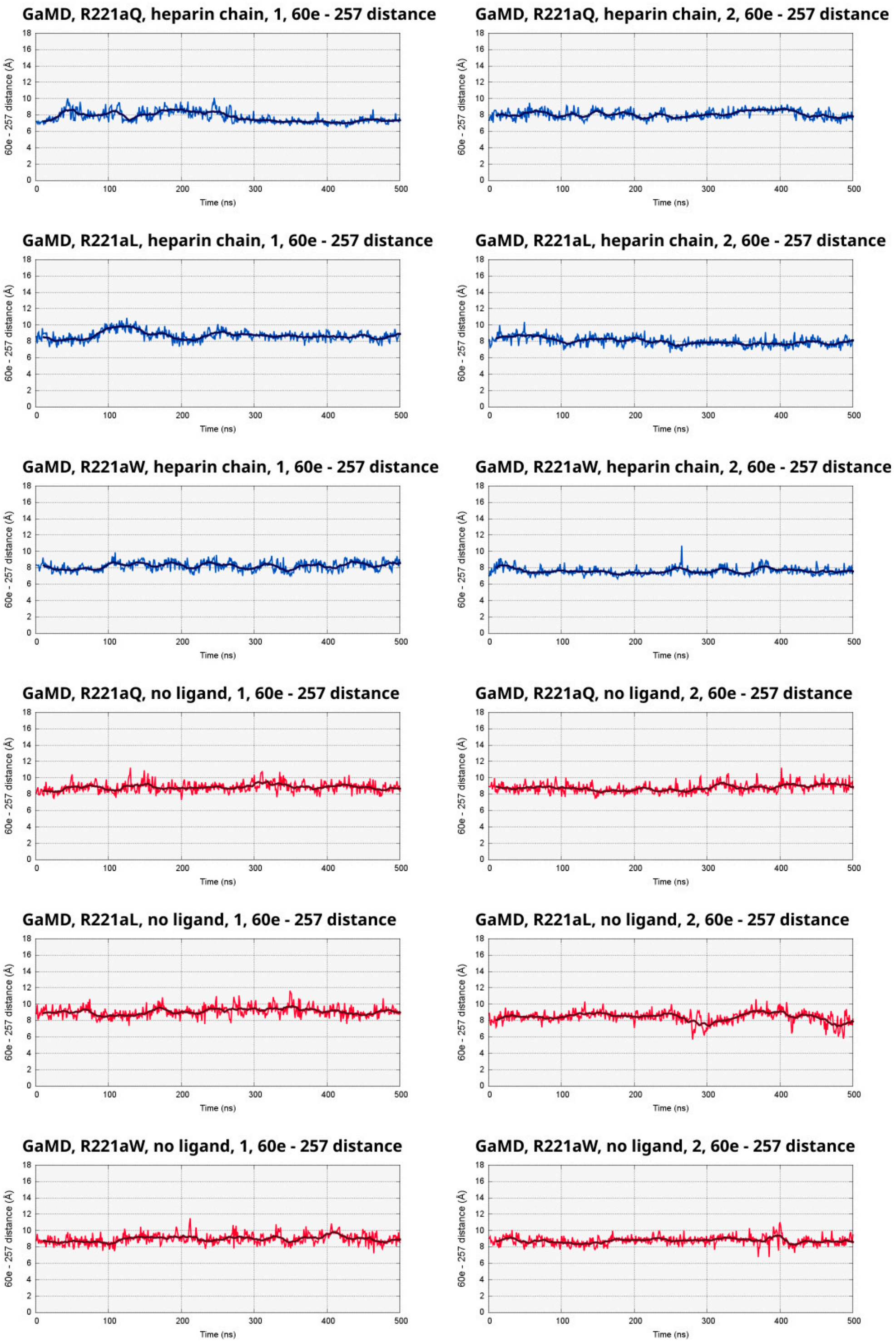

Figure S24. Distance between the alpha carbon atoms of amino acid 60e in thrombin and 257 in antithrombin, as a function of time, in the GaMD simulations of antithrombin complexes with thrombin mutants.

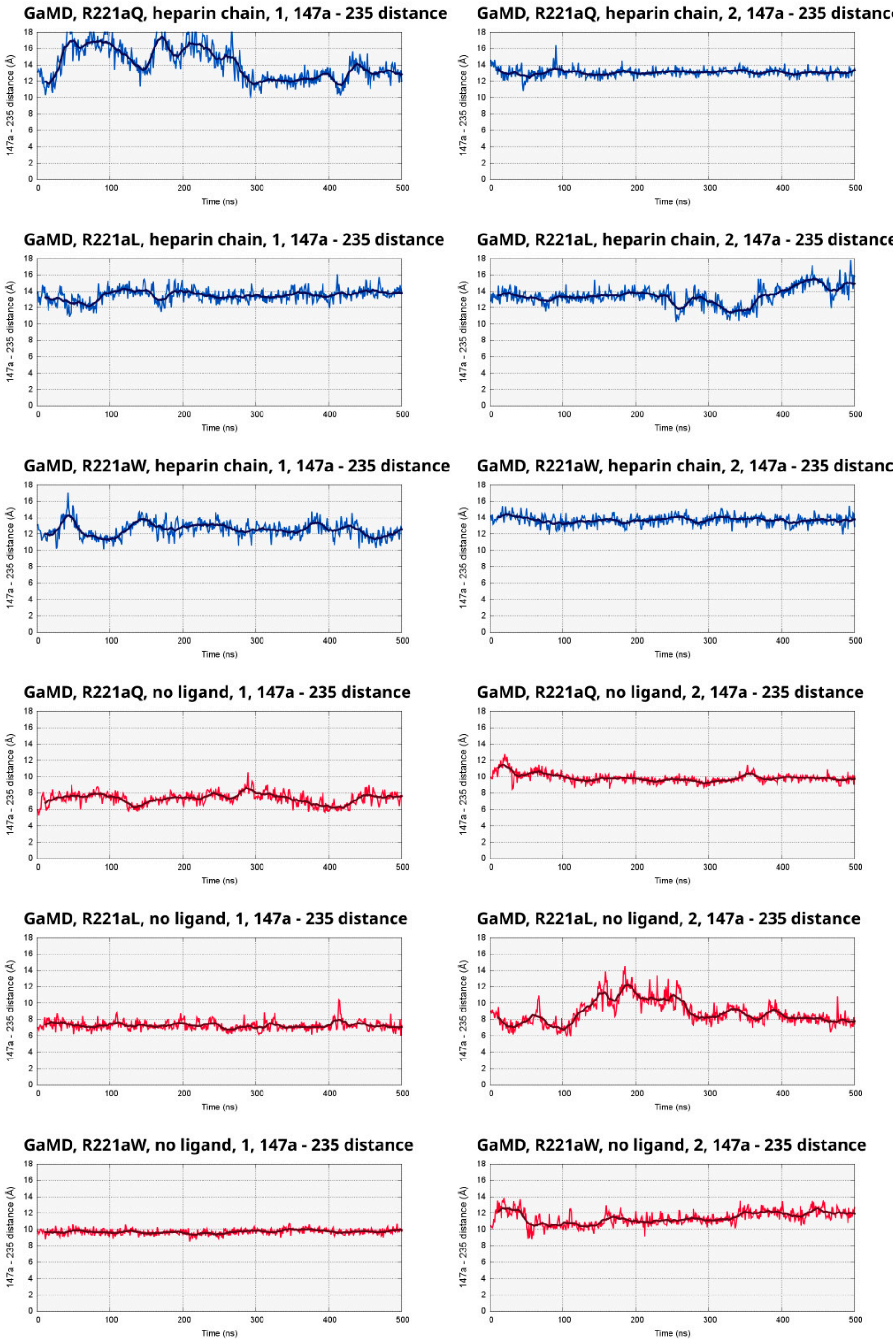

Figure S25. Distance between the alpha carbon atoms of amino acid 147a in thrombin and 235 in antithrombin, as a function of time, in the GaMD simulations of antithrombin complexes with thrombin mutants.

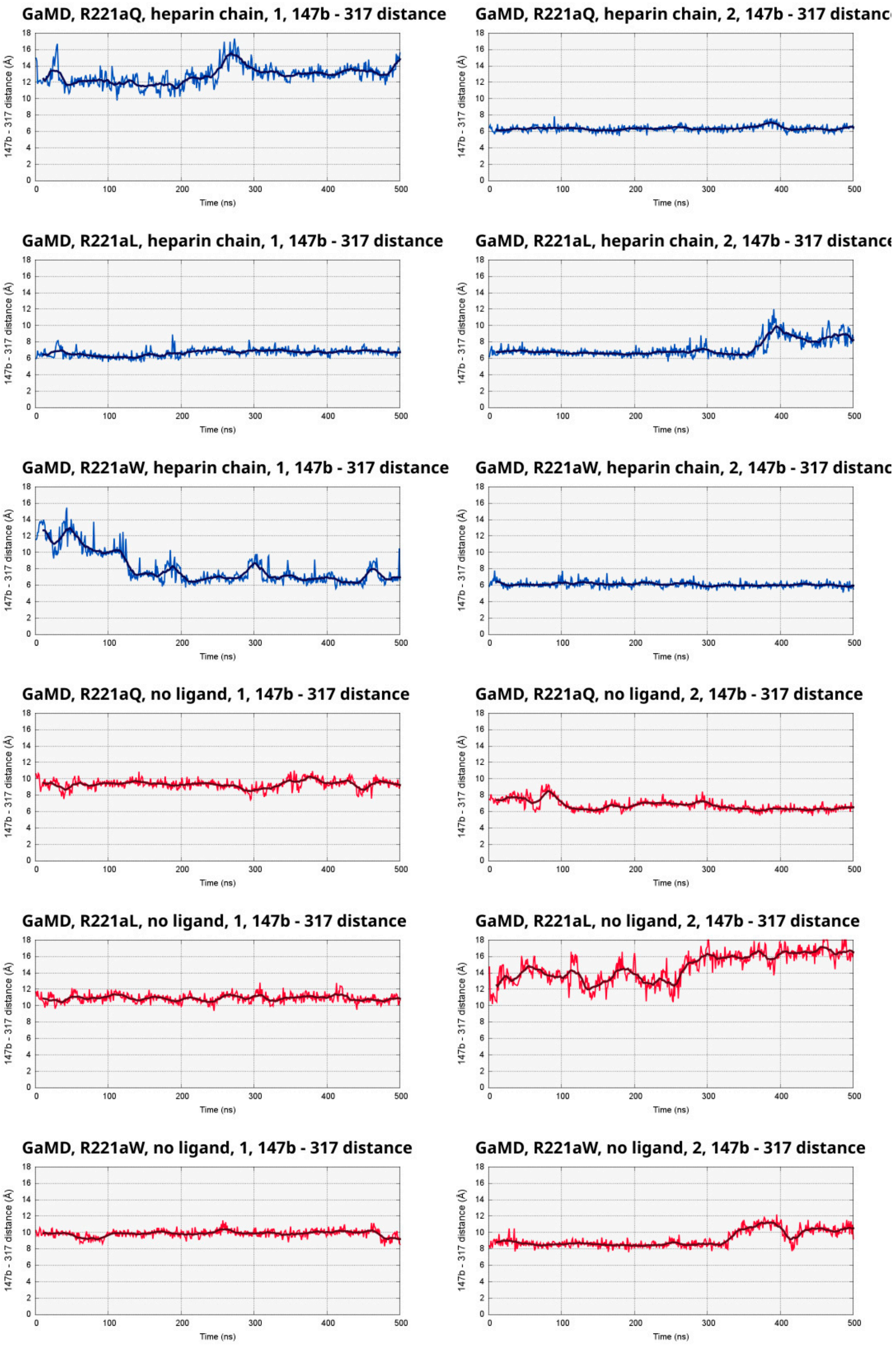

Figure S26. Distance between the alpha carbon atoms of amino acid 147b in thrombin and 317 in antithrombin, as a function of time, in the GaMD simulations of antithrombin complexes with thrombin mutants.

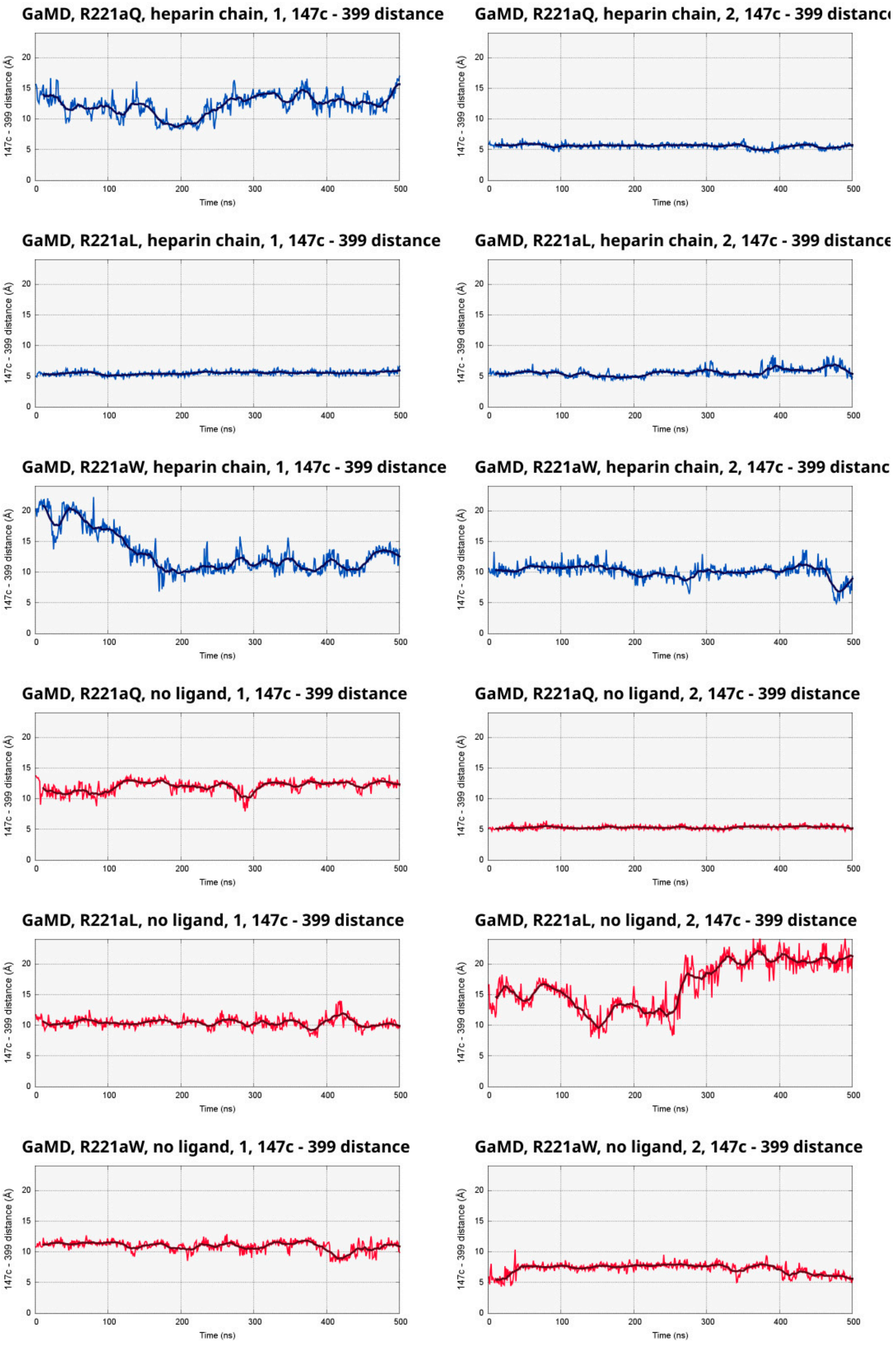

Figure S27. Distance between the alpha carbon atoms of amino acid 147c in thrombin and 399 in antithrombin, as a function of time, in the GaMD simulations of antithrombin complexes with thrombin mutants.

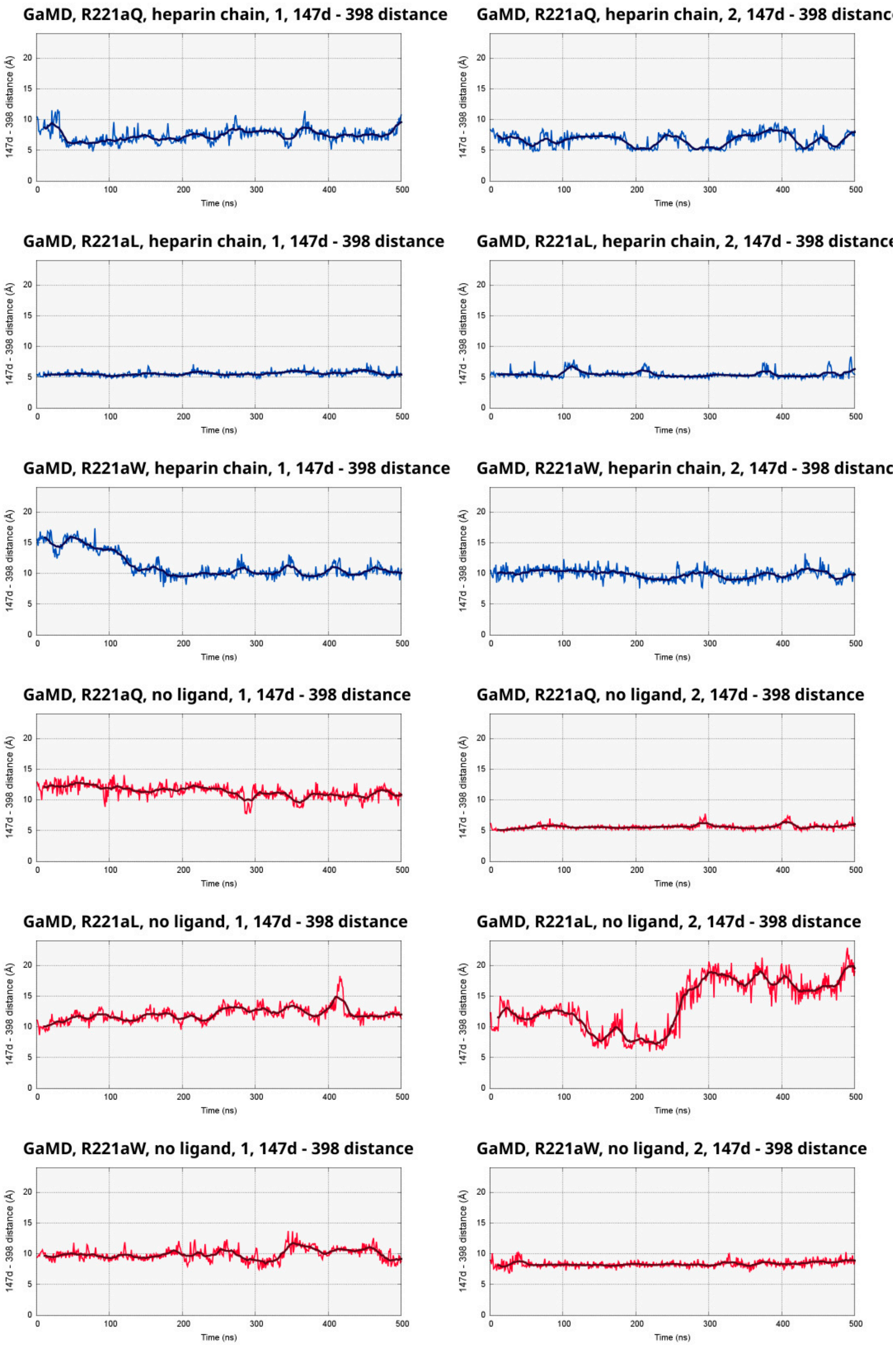

Figure S28. Distance between the alpha carbon atoms of amino acid 147d in thrombin and 398 in antithrombin, as a function of time, in the GaMD simulations of antithrombin complexes with thrombin mutants.

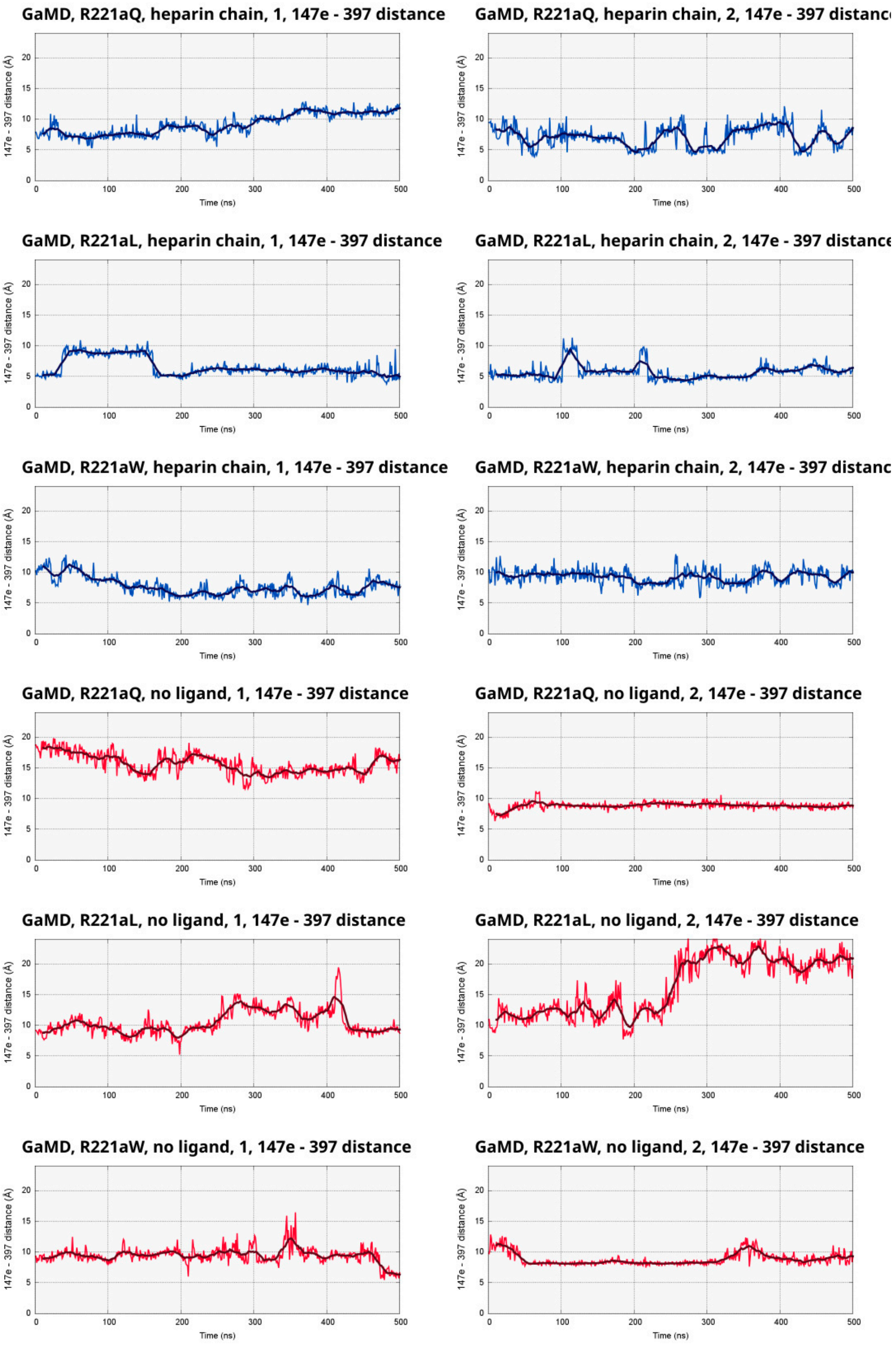

Figure S29. Distance between the alpha carbon atoms of amino acid 147e in thrombin and 397 in antithrombin, as a function of time, in the GaMD simulations of antithrombin complexes with thrombin mutants.

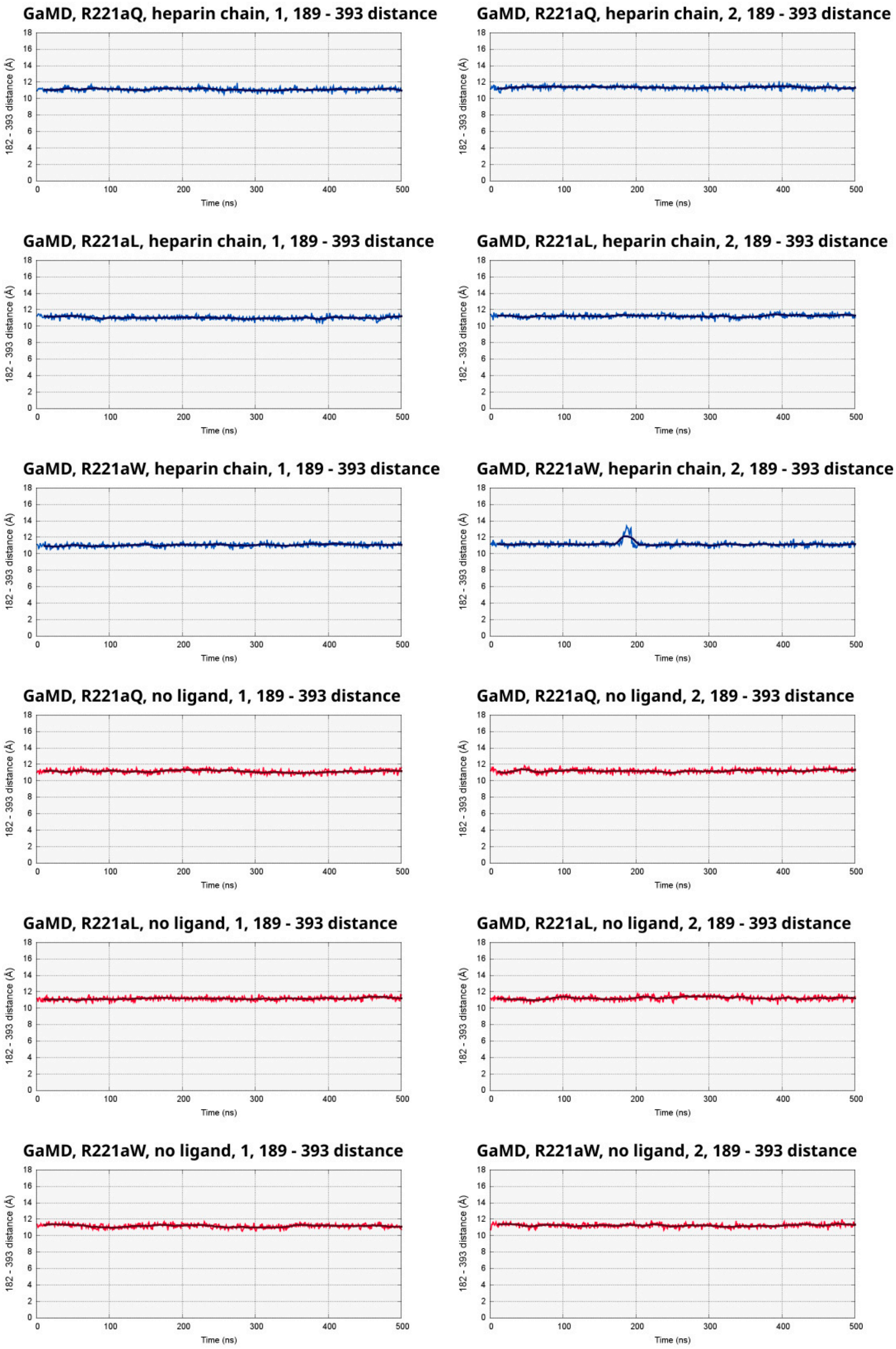

Figure S30. Distance between the alpha carbon atoms of amino acid 189 in thrombin and 393 in antithrombin, as a function of time, in the GaMD simulations of antithrombin complexes with thrombin mutants.

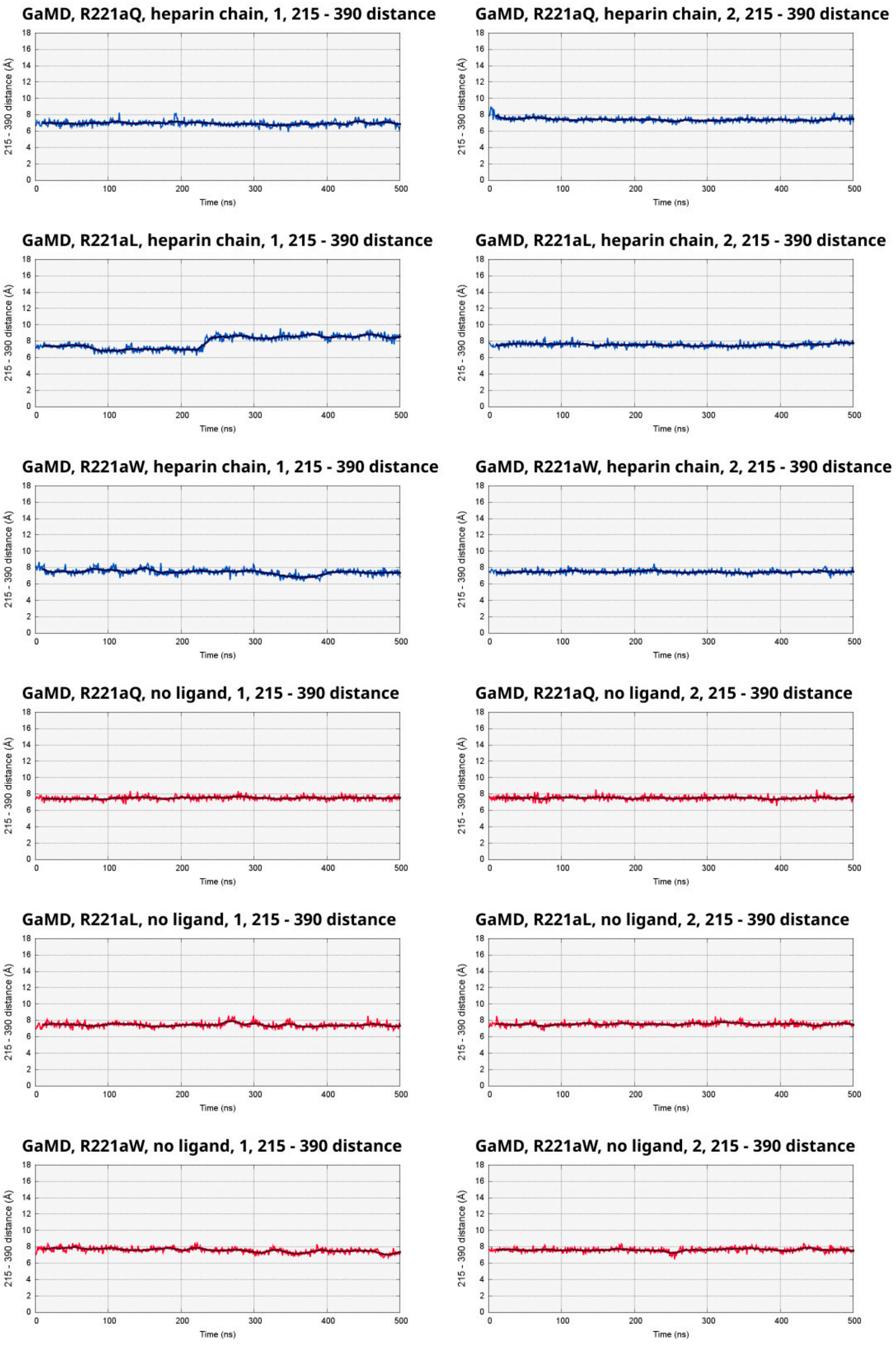

Figure S31. Distance between the alpha carbon atoms of amino acid 215 in thrombin and 390 in antithrombin, as a function of time, in the GaMD simulations of antithrombin complexes with thrombin mutants.

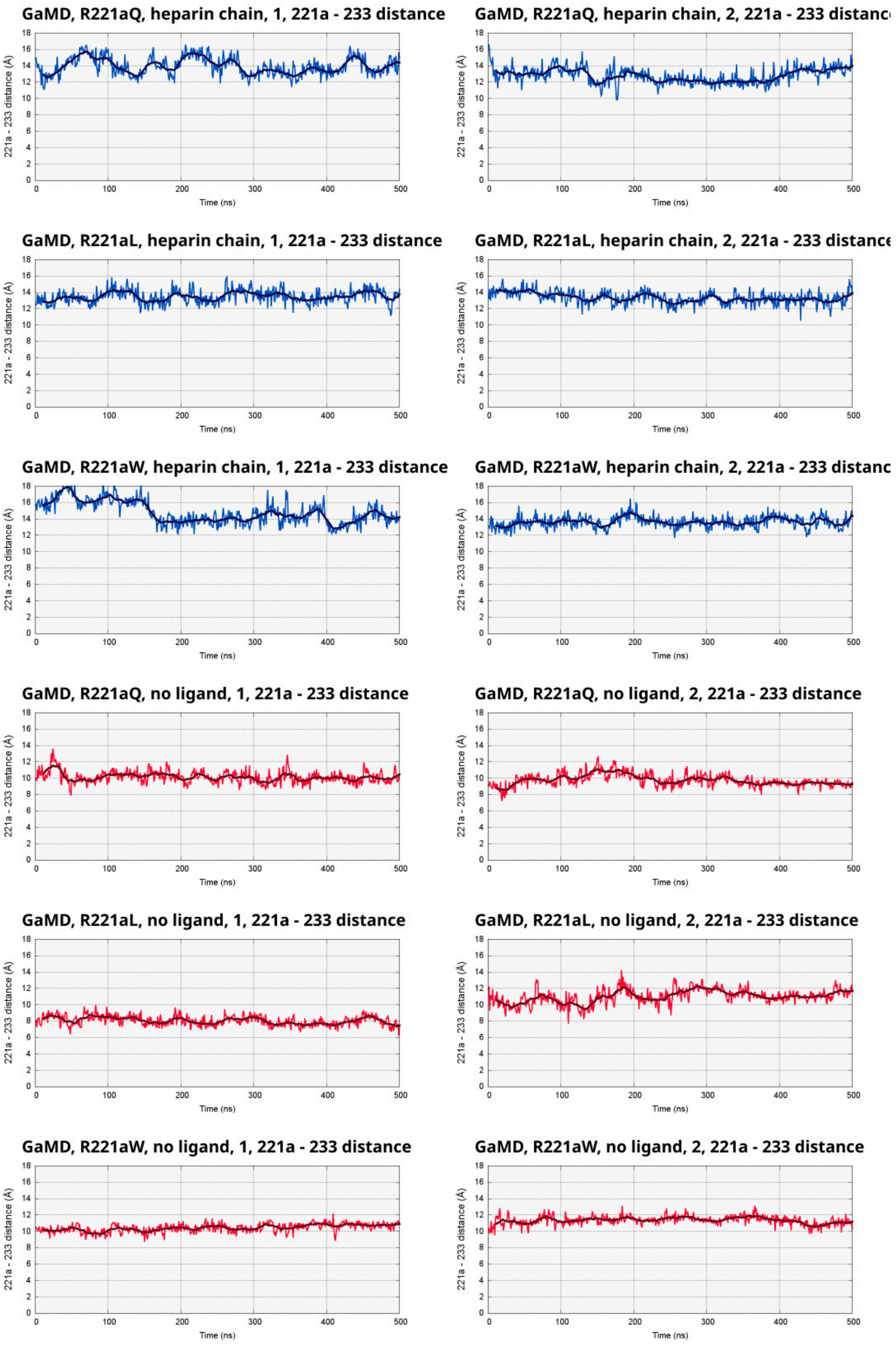

Figure S32. Distance between the alpha carbon atoms of amino acid 221a in thrombin and 233 in antithrombin, as a function of time, in the GaMD simulations of antithrombin complexes with thrombin mutants.

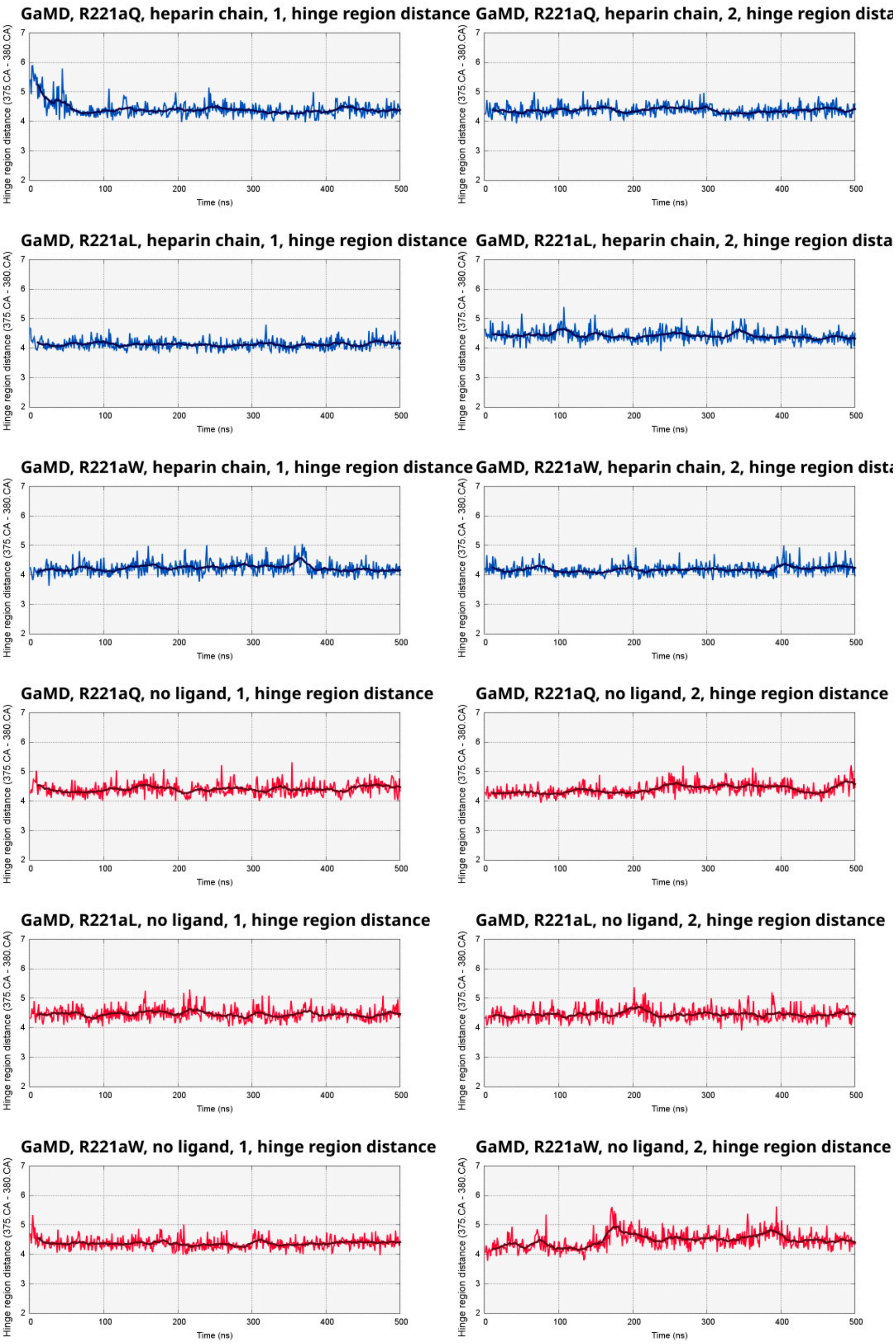

Figure S33. Distance between the alpha carbon atoms of amino acids 375 and 380 in antithrombin, corresponding to the position of the hinge region, as a function of time, in the GaMD simulations of antithrombin complexes with thrombin mutants.

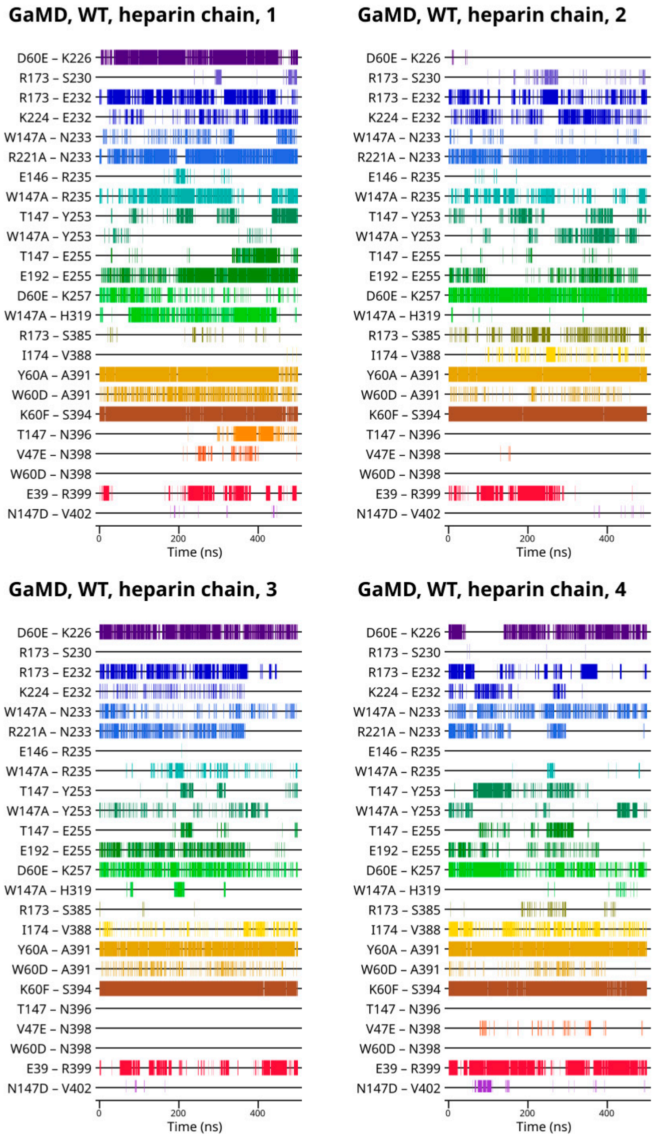

Figure S34A. The amino acids involved in interactions between the catalytic domain of thrombin and antithrombin (distances below 5 Å) as a function of time, in the GaMD simulations of antithrombin complexes with wild type (WT) thrombin. In this part of the figure, the data from the simulations containing a heparin chain are shown.

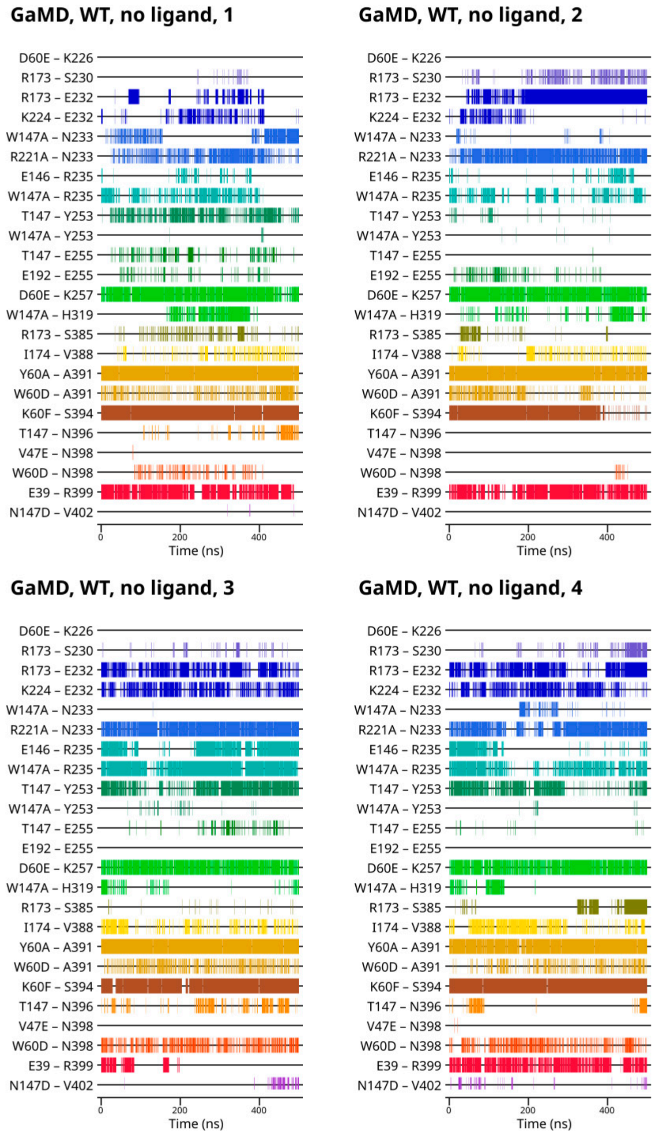

Figure S34B. The amino acids involved in interactions between the catalytic domain of thrombin and antithrombin (distances below 5 Å) as a function of time, in the GaMD simulations of antithrombin complexes with wild type (WT) thrombin. In this part of the figure, the data from the simulations without any ligand are shown.

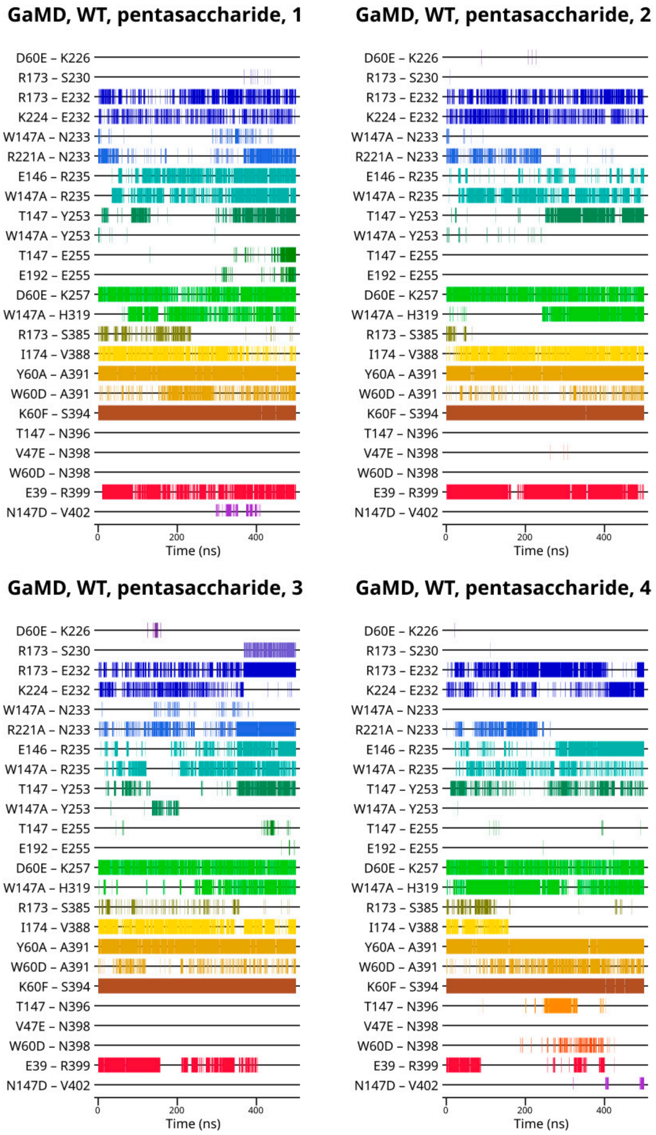

Figure S34C. The amino acids involved in interactions between the catalytic domain of thrombin and antithrombin (distances below 5 Å) as a function of time, in the GaMD simulations of antithrombin complexes with wild type (WT) thrombin. In this part of the figure, the data from the simulations containing a pentasaccharide are shown.

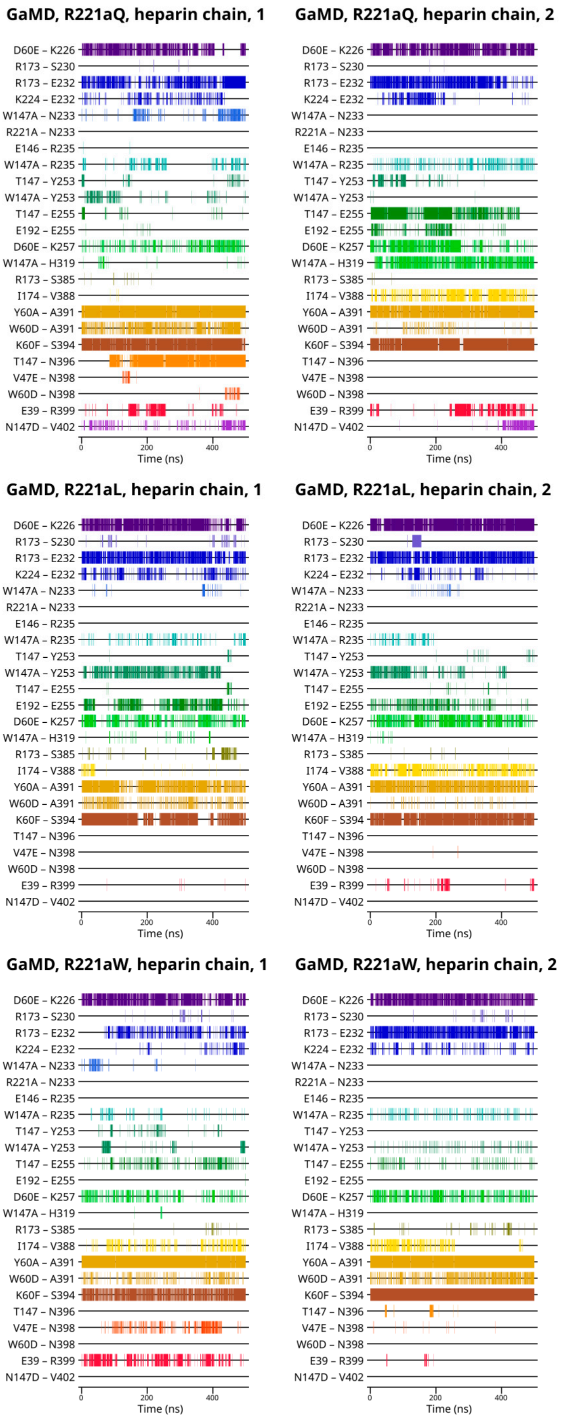

Figure S35A. The amino acids involved in interactions between the catalytic domain of thrombin and antithrombin (distances below 5 Å) as a function of time, in the GaMD simulations of antithrombin complexes with thrombin mutants. In this part of the figure, the data from the simulations containing a heparin chain are shown.

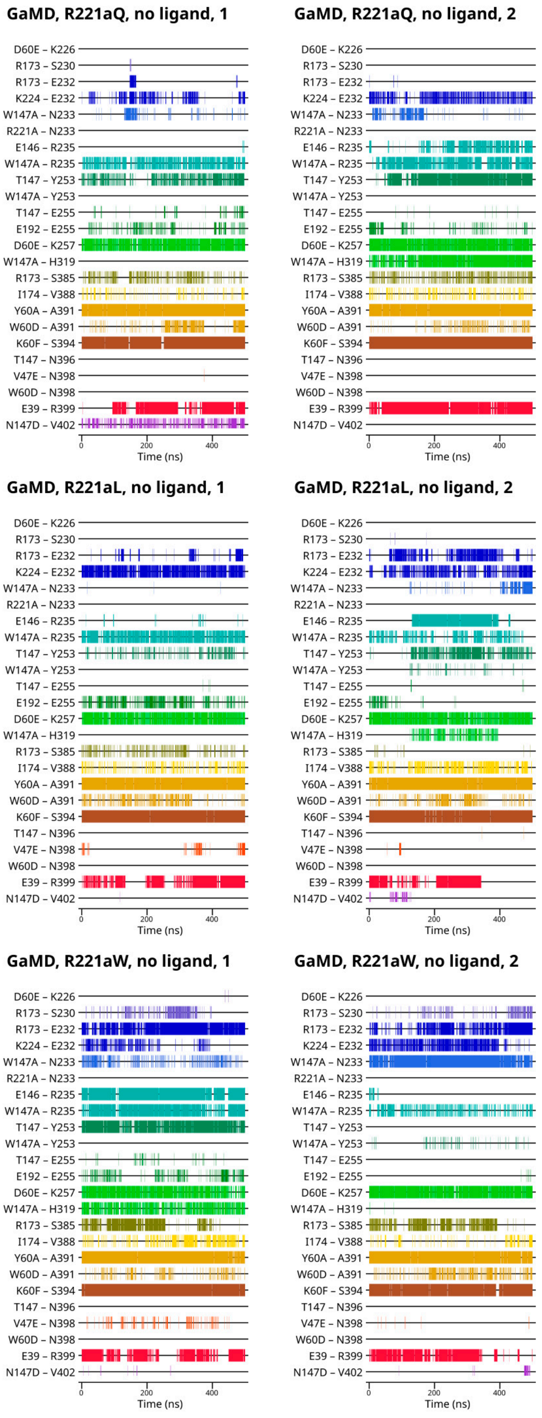

Figure S35B. The amino acids involved in interactions between the catalytic domain of thrombin and antithrombin (distances below 5 Å) as a function of time, in the GaMD simulations of antithrombin complexes with thrombin mutants. In this part of the figure, the data from the simulations without any ligand are shown.

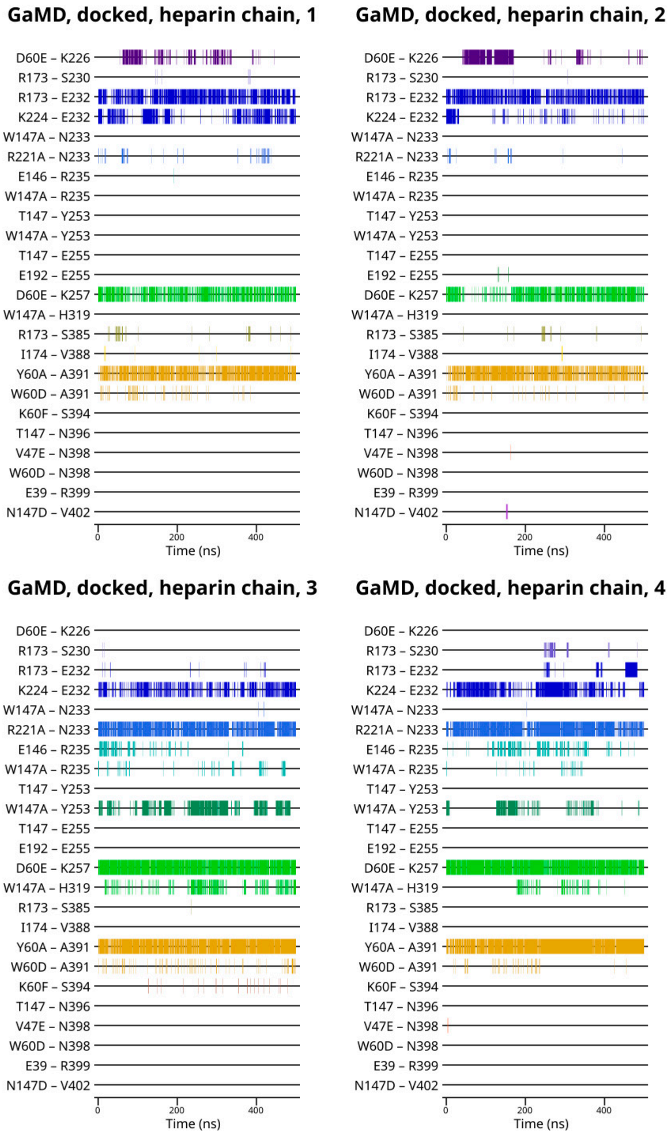

Figure S36A. The amino acids involved in interactions between the catalytic domain of thrombin and antithrombin (distances below 5 Å) as a function of time, in the GaMD simulations of antithrombin-thrombin complexes built using docking. In this part of the figure, the data from the simulations containing a heparin chain are shown.

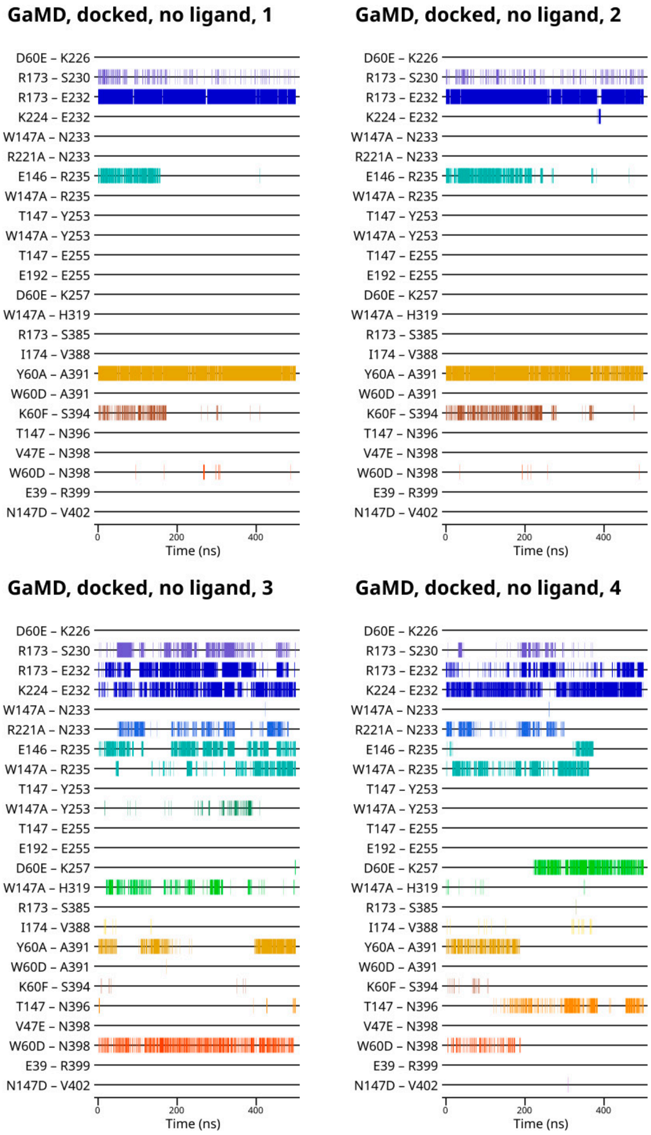

Figure S36B. The amino acids involved in interactions between the catalytic domain of thrombin and antithrombin (distances below 5 Å) as a function of time, in the GaMD simulations of antithrombin-thrombin complexes built using docking. In this part of the figure, the data from the simulations without any ligand are shown.

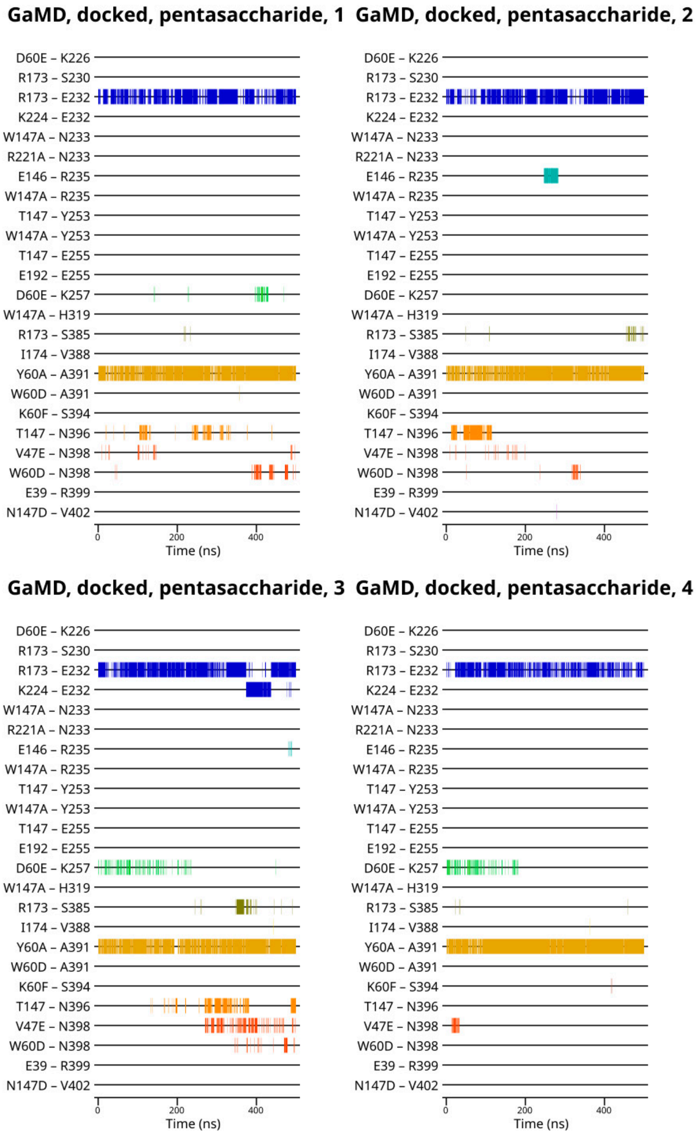

Figure S36C. The amino acids involved in interactions between the catalytic domain of thrombin and antithrombin (distances below 5 Å) as a function of time, in the GaMD simulations of antithrombin-thrombin complexes built using docking. In this part of the figure, the data from the simulations containing a pentasaccharide are shown.

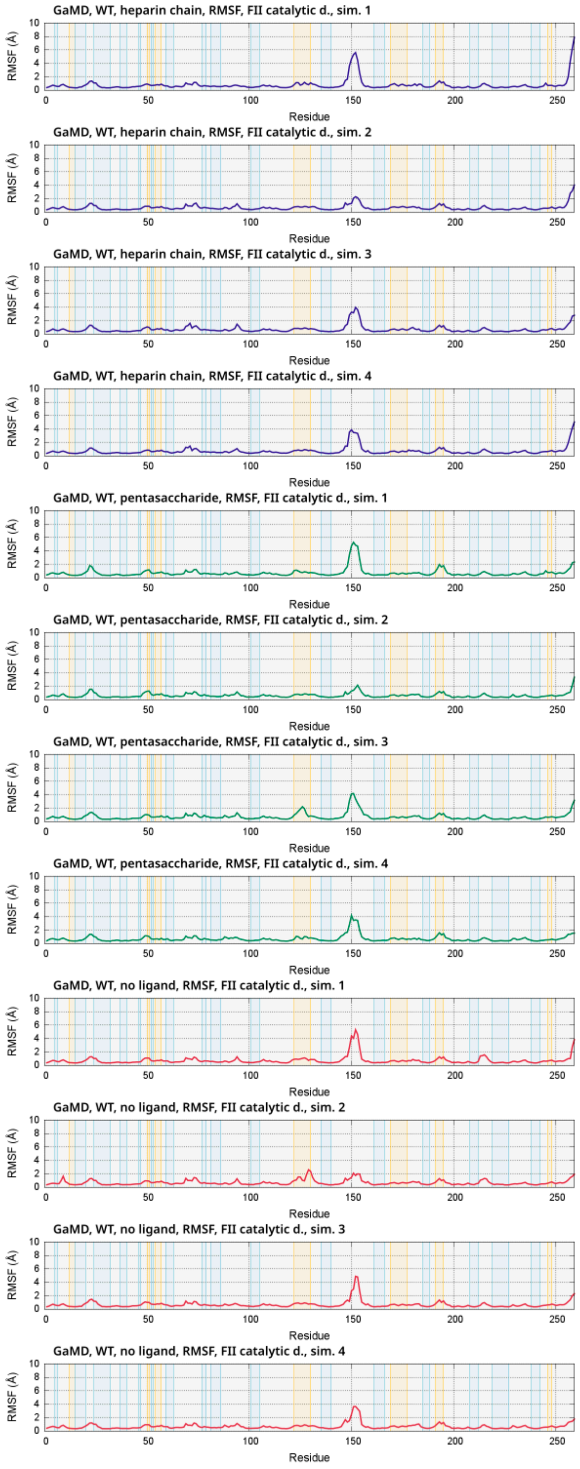

Figure S37. Root mean fluctuations of the alpha-carbon atoms in the catalytic domain of thrombin, calculated from the GaMD simulations of antithrombin complexes with wild type (WT) thrombin.

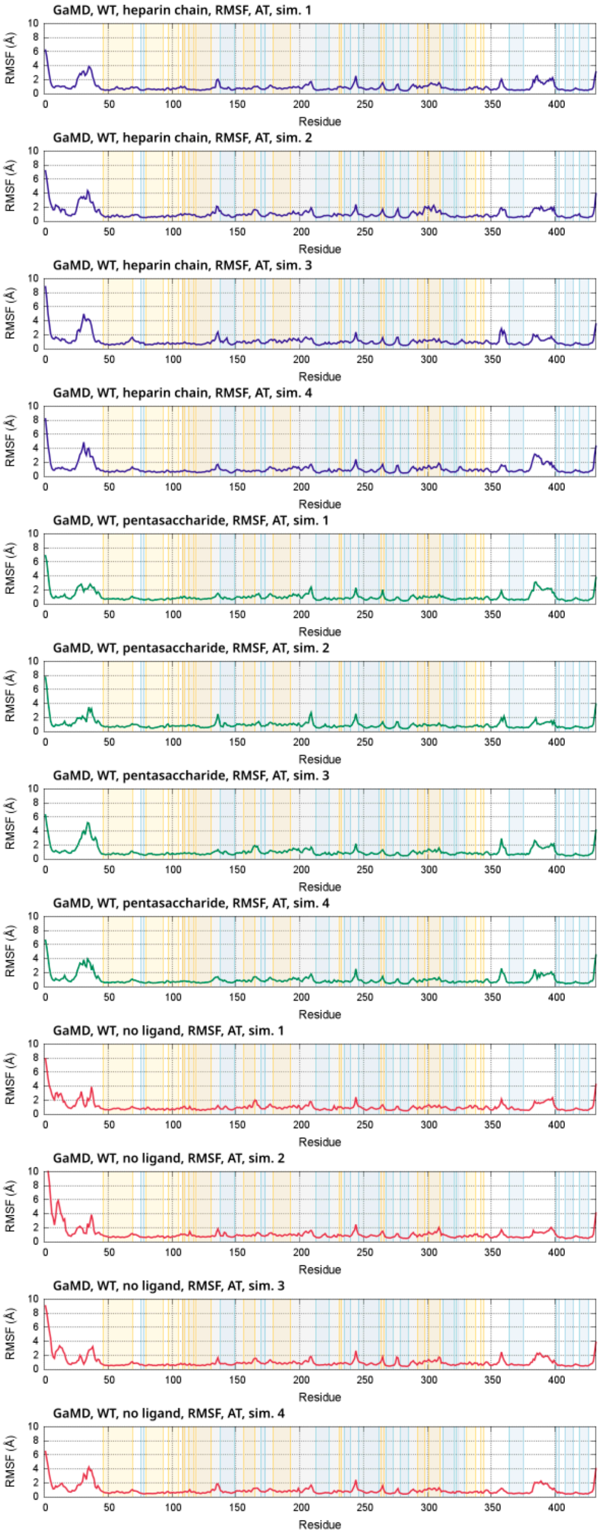

Figure S38. Root mean fluctuations of the alpha-carbon atoms in antithrombin, calculated from the GaMD simulations of antithrombin complexes with wild type (WT) thrombin.

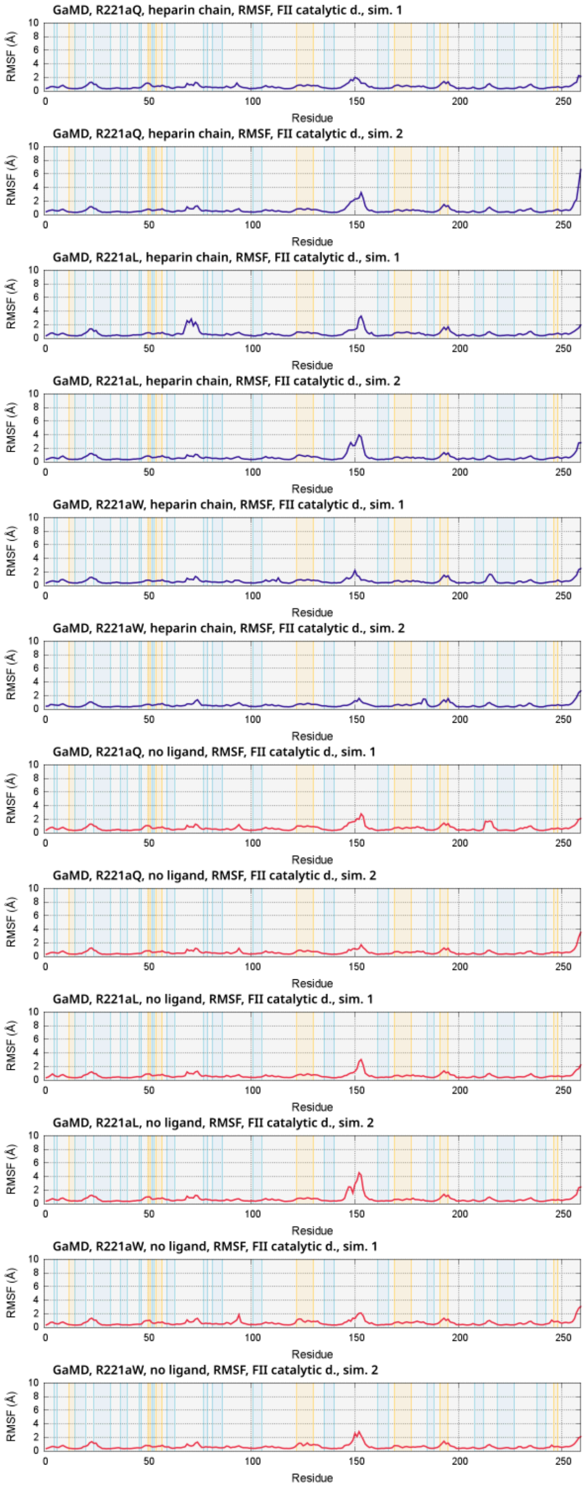

Figure S39. Root mean fluctuations of the alpha-carbon atoms in the catalytic domain of thrombin, calculated from the GaMD simulations of antithrombin complexes with thrombin mutants.

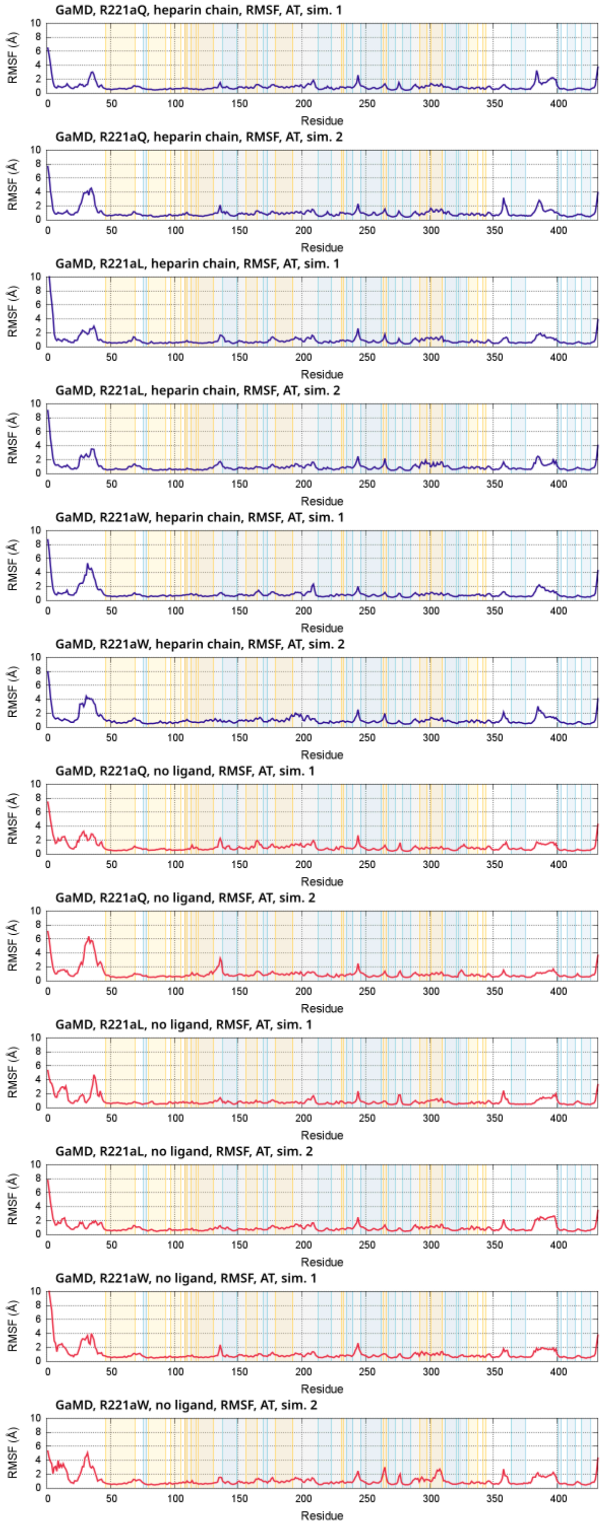

Figure S40. Root mean fluctuations of the alpha-carbon atoms in antithrombin, calculated from the GaMD simulations of antithrombin complexes with thrombin mutants.

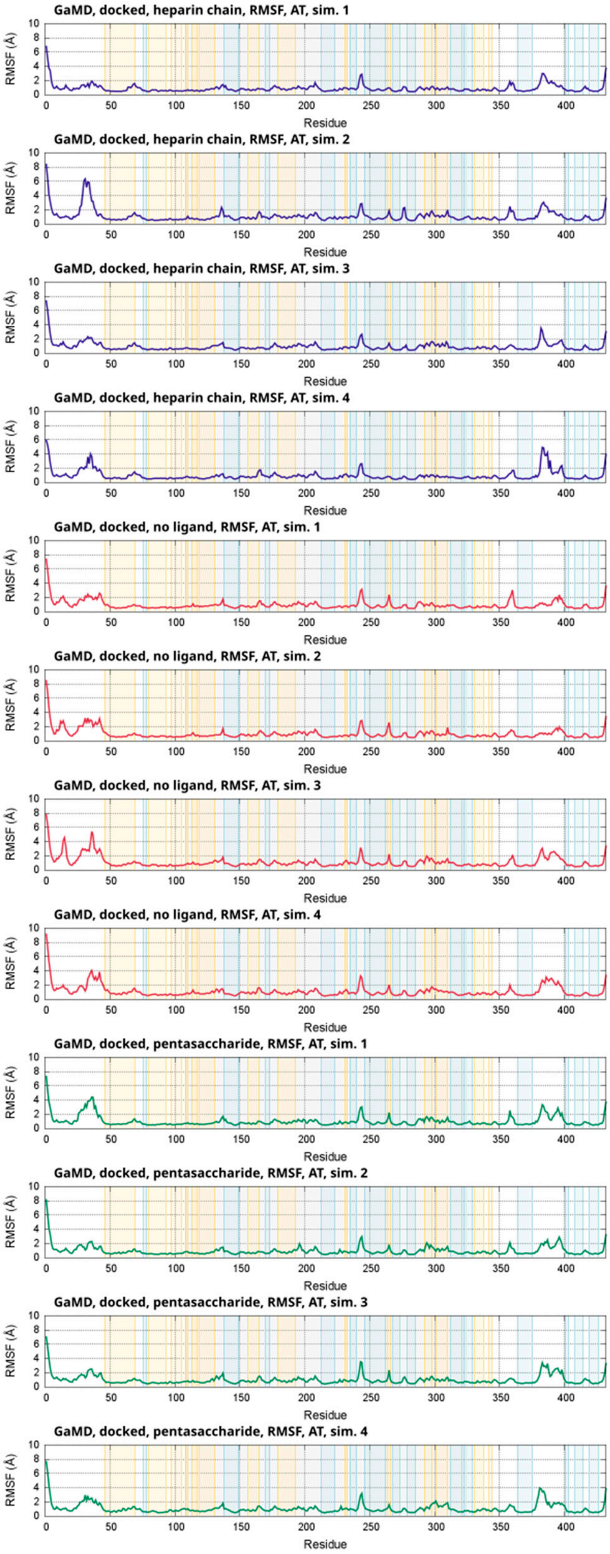

Figure S41. Root mean fluctuations of the alpha-carbon atoms in antithrombin, calculated from the GaMD simulations of the antithrombin-thrombin complexes built using docking.

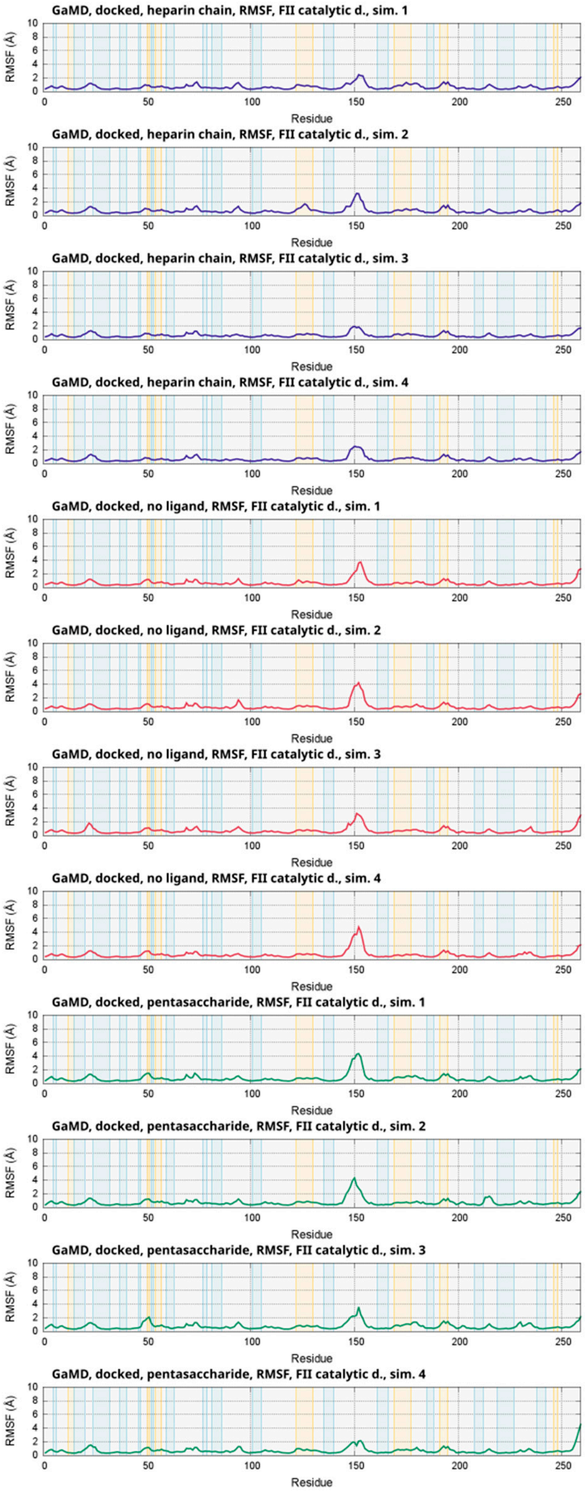

Figure S42. Root mean fluctuations of the alpha-carbon atoms in the catalytic domain of thrombin, calculated from the GaMD simulations of the antithrombin-thrombin complexes built using docking.

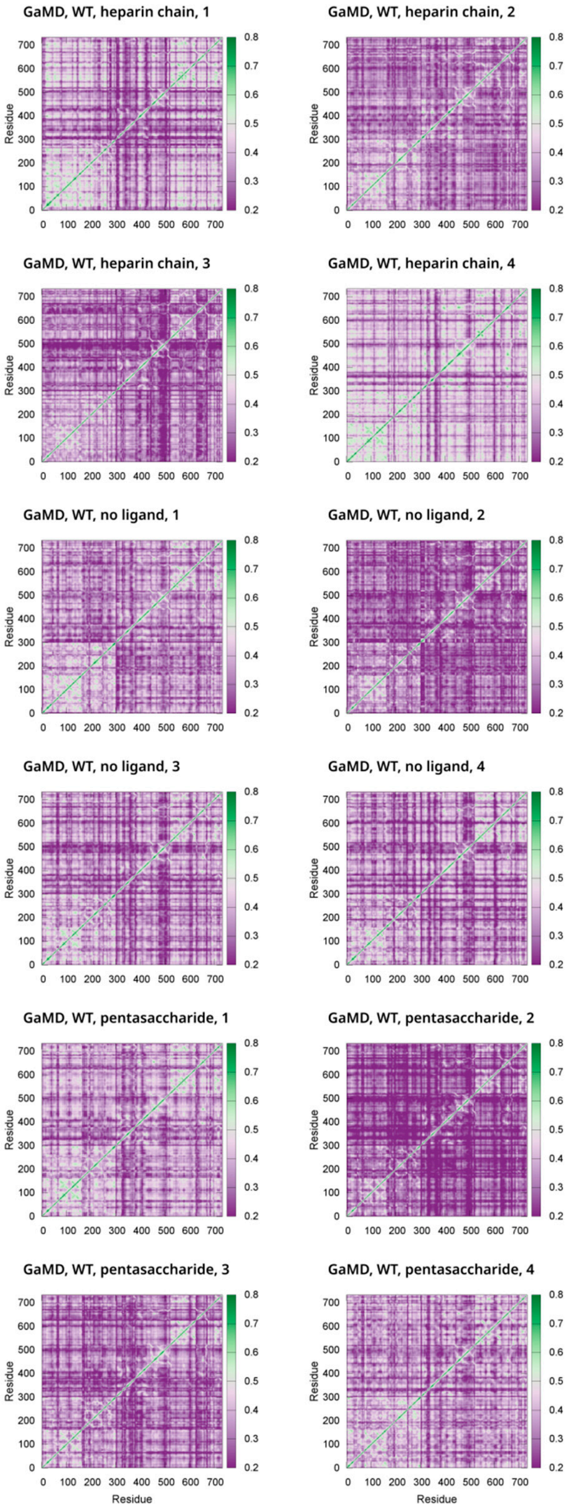

Figure S43. Correlated motions of the alpha-carbon atoms in the GaMD simulations of antithrombin complexes with wild type (WT) thrombin, computed using a method proposed by Lange and Grubmüller.

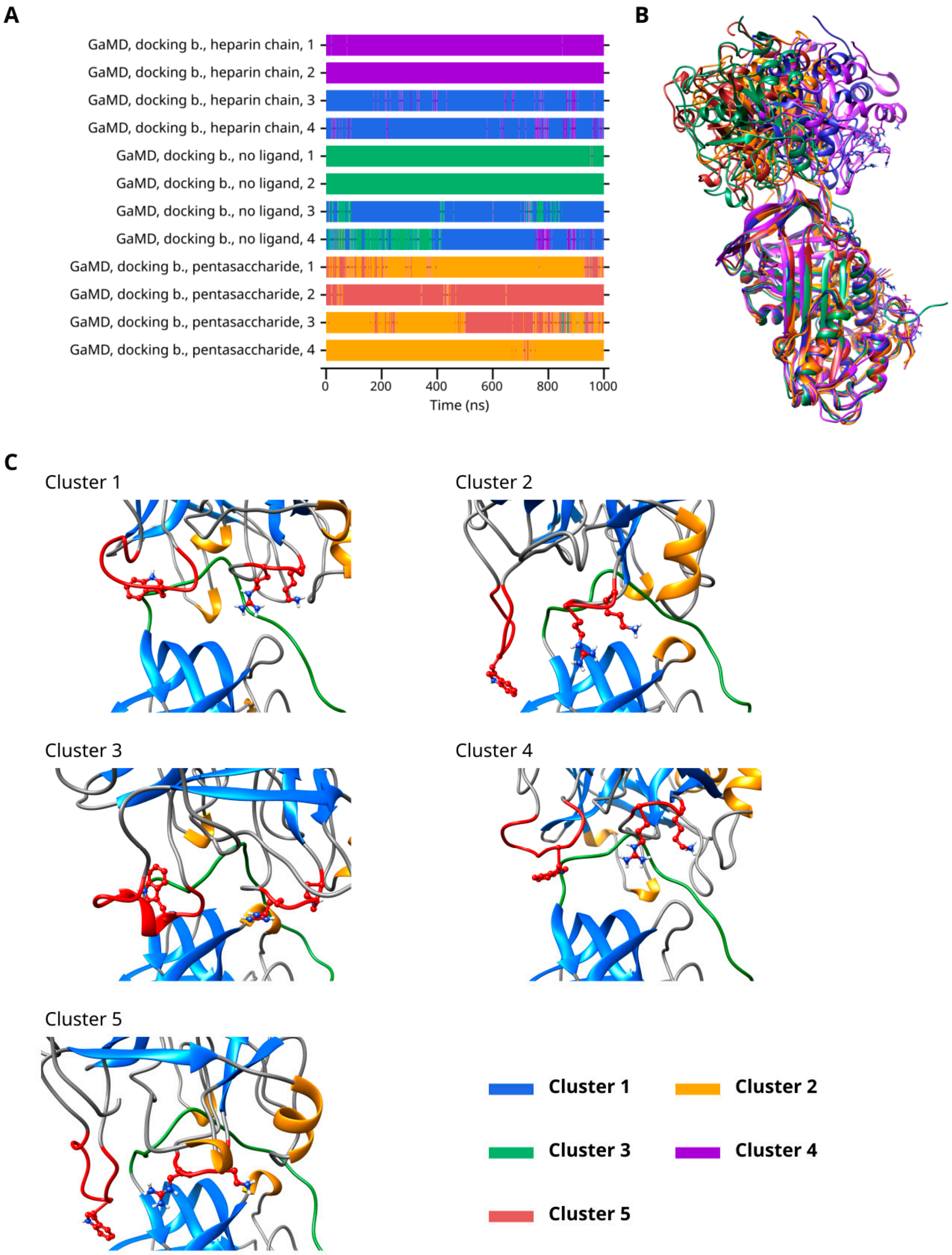

Figure S44. **A**. Cluster number, as a function of time in the GaMD simulations of the AT-thrombin complexes based ont he structure obtained by docking. **B**. Superposition of the representative frames from each cluster. **C**. The interaction between the exosites of thrombin and AT in the 4 clusters.

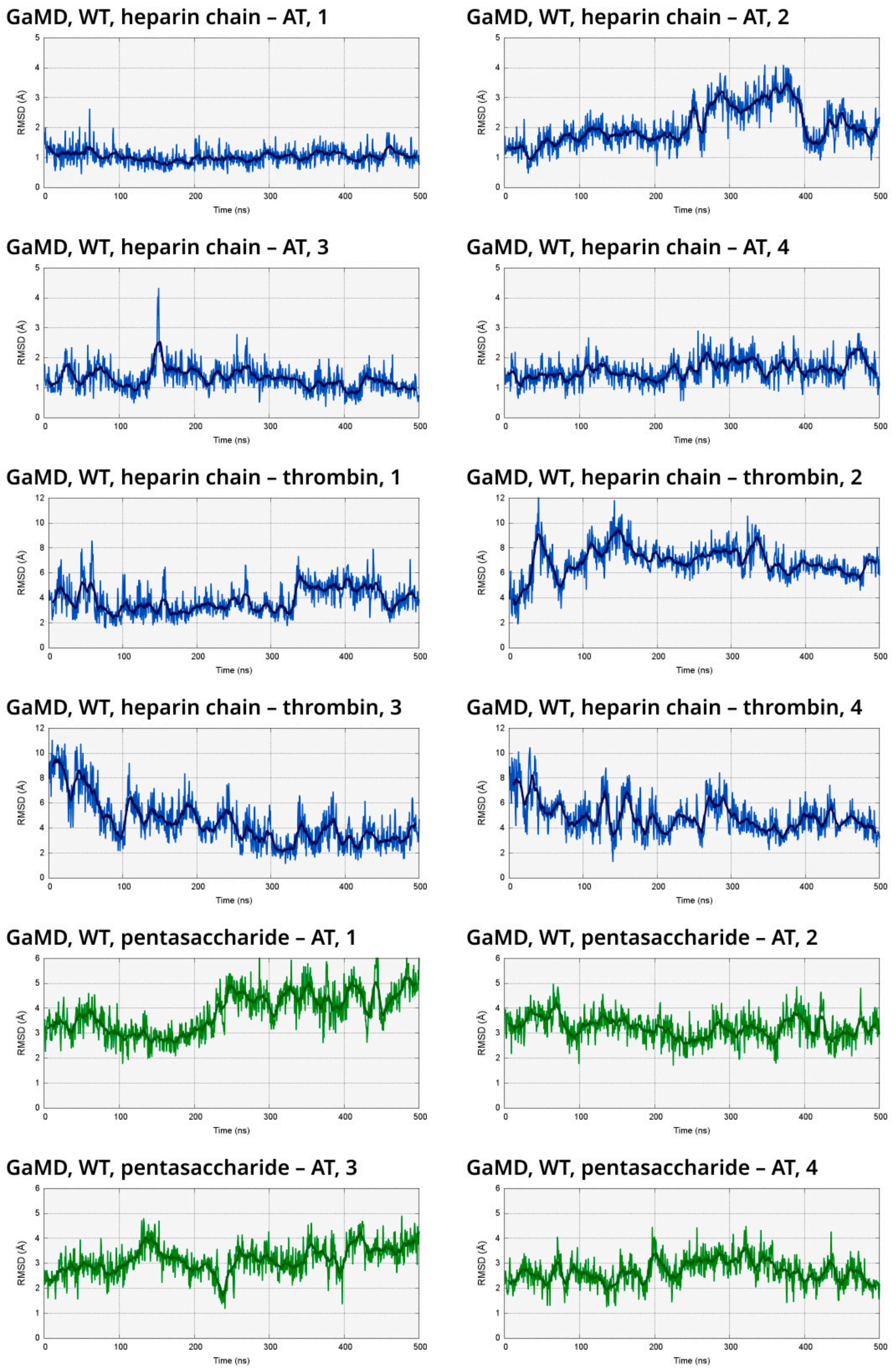

Figure S45. The binding site of the of the heparinoid ligands (either heparin chain or pentasaccharide) on either AT or thrombin to their position in the X-ray diffraction structure, in the simulations of wild type (WT) AT-thrombin complexes. The conformational differences were expressed as RMSD.

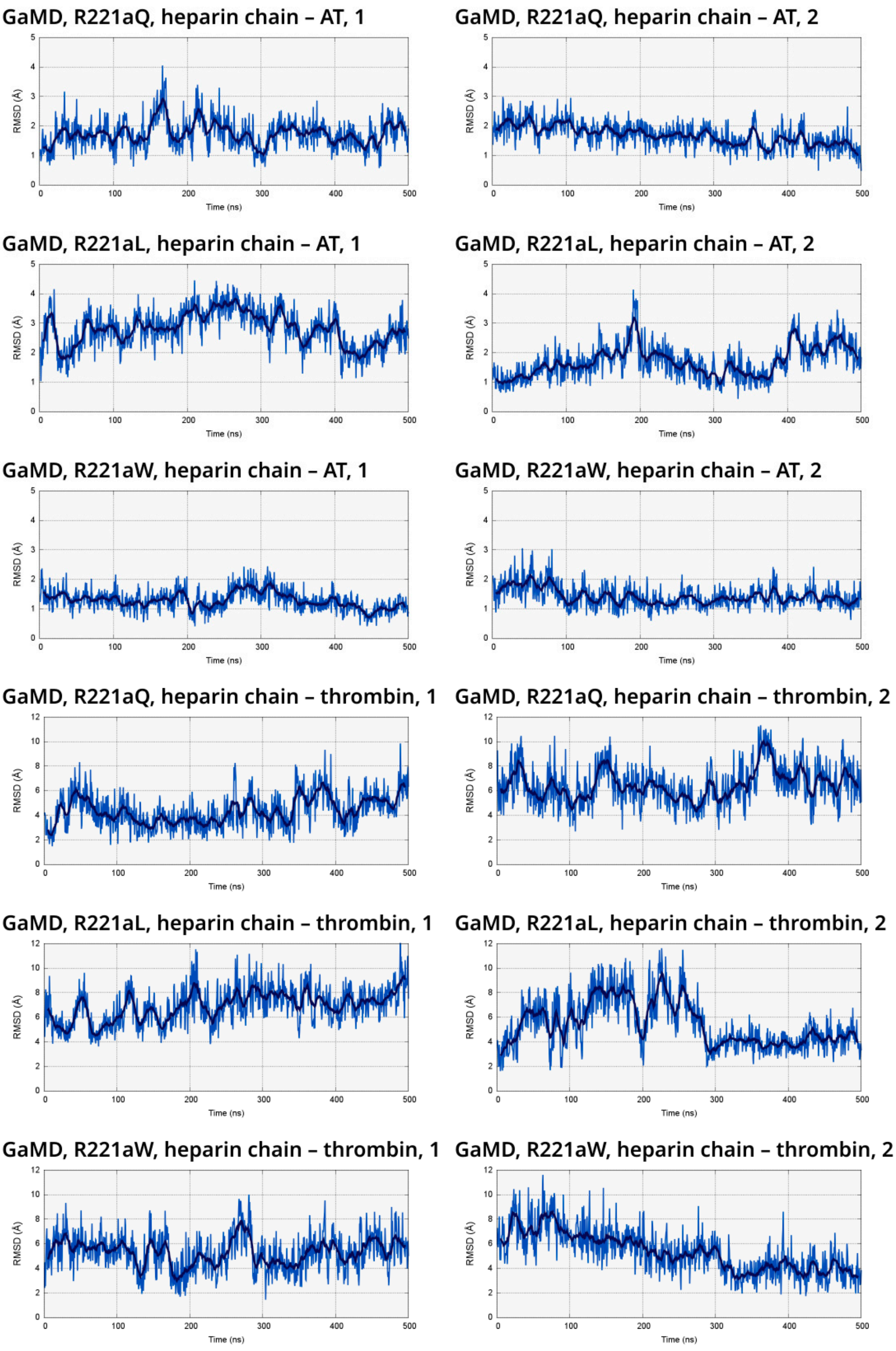

Figure S46. The binding site of the of the heparinoid ligands (heparin chain) on either AT or thrombin to their position in the X-ray diffraction structure, in the simulation of the three thrombin mutants with AT. The conformational differences were expressed as RMSD.

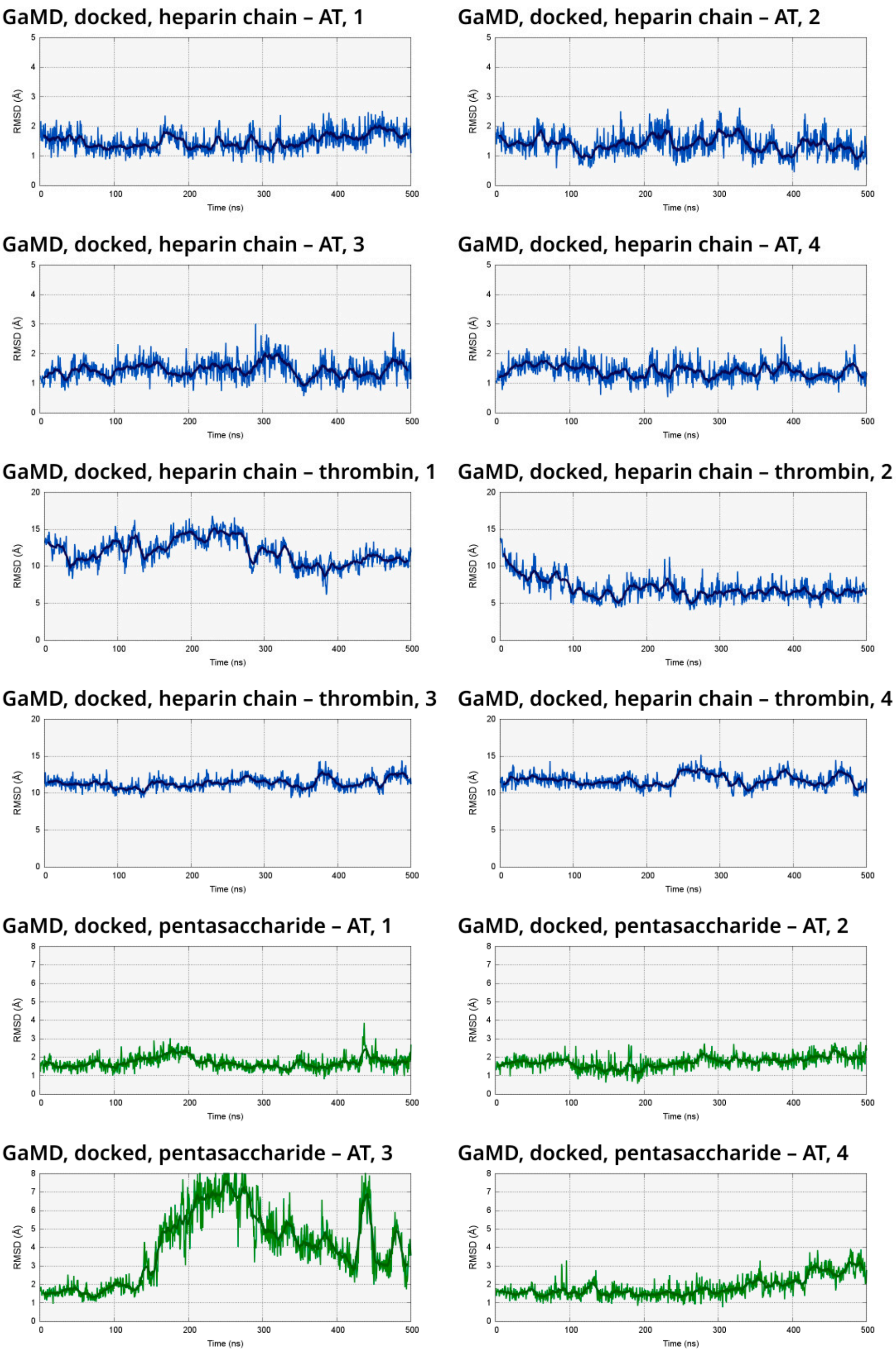

Figure S47. The binding site of the of the heparinoid ligands (either heparin chain or pentasaccharide) on either AT or thrombin to their position in the starting structure, from the simulations based on the structures obtained by docking. The conformational differences were expressed as RMSD.
